# Regular versus as-needed treatments for mild asthma in children, adolescents, and adults: a systematic review and network meta-analysis

**DOI:** 10.1186/s12916-025-03847-z

**Published:** 2025-01-21

**Authors:** Prapaporn Pornsuriyasak, Sunatee Sa-nguansai, Kunlawat Thadanipon, Pawin Numthavaj, Gareth J. McKay, John Attia, Ammarin Thakkinstian

**Affiliations:** 1 Department of Clinical Epidemiology and Biostatistics, Faculty of Medicine, Ramathibodi Hospital, Mahidol University, Bangkok, 10400 Thailand; 2 Oncology Unit, Department of Medicine, Rajavithi Hospital, College of Medicine, Rangsit University, Bangkok, 10400 Thailand; 3 Centre for Public Health, School of Medicine, Dentistry, and Biomedical Sciences, Queen’s University Belfast, Belfast, Northern Ireland; 4 Centre for Clinical Epidemiology and Biostatistics, School of Medicine and Public Health, University of Newcastle, Newcastle, NSW Australia; 5 Division of Pulmonary and Critical Care, Department of Medicine, Faculty of Medicine, Ramathibodi Hospital, Mahidol University, Bangkok, Thailand

**Keywords:** As-needed use, Inhaled treatments, Intermittent asthma, Mild asthma, Network meta-analysis, Regular use

## Abstract

**Background:**

Inhaled corticosteroids (ICS) are recommended treatment for mild asthma. We aimed to update the evidence on the efficacy and safety of ICS-containing regimens, leukotriene receptor antagonists (LTRA), and tiotropium relative to as-needed (AN) short-acting β2-agonists (SABA) in children (aged 6–11 years) and adolescents/adults.

**Methods:**

A systematic review of randomized controlled trials (RCTs) of regular and AN treatment for mild asthma was conducted (CRD42022352384). PubMed, Scopus, and ClinicalTrials.gov were searched up to 31st March 2024. RCTs in children or adolescents/adults with mild asthma were eligible if they compared any of the following treatments: ICS alone or in combination with fast-acting bronchodilators (FABA, i.e., formoterol or SABA) or long-acting β2-agonists (LABA), LTRA, tiotropium, and SABA alone, for the following outcomes: exacerbations, asthma symptoms, forced expiratory volume in 1 s (FEV_1_), asthma-specific quality-of-life (QoL), or severe adverse events (SAEs). The two-stage network meta-analysis (NMA) was used to pool risk ratios (RR) or mean differences for treatment outcomes. The risk of bias was assessed using the Revised Cochrane risk-of-bias tool for randomized trials (RoB2). This review followed the PRISMA reporting guideline and the PRISMA checklist is presented in Additional file 2.

**Results:**

Thirteen RCTs in children and 29 in adolescents/adults were included. Regular ICS ranked best for preventing exacerbations and improving FEV_1_ in children. NMA of RCTs suggested regular ICS were better in preventing exacerbations than LTRA (RR [95% confidence intervals], (0.81 [0.69,0.96]) and AN-SABA (0.61 [0.48,0.78]), and not different from AN-ICS (0.83 [0.62,1.12]). In adolescents/adults, for preventing severe exacerbations, regular ICS outperformed AN-SABA (0.58 [0.46,0.73]), but AN-ICS/FABA (0.73 [0.54,0.97]), and regular ICS/LABA (0.68 [0.48,0.97]) surpassed regular ICS. Symptom relief and improved FEV_1_ were not different among the ICS-containing regimens. Regular ICS ranked best for improved QoL and least likely for SAEs.

**Conclusions:**

Regular ICS use may be the most effective treatment for preventing exacerbation and increasing FEV_1_ in children with mild asthma. In adolescents/adults, ICS-containing regimens outperformed AN-SABA for exacerbation prevention. With varying degrees of heterogeneity, severe exacerbation risk in adolescents/adults might be lower with regular ICS/LABA or AN-ICS/FABA than regular ICS, where AN-ICS/FABA may not be suitable for patients with low FEV_1_. Additionally, regular ICS use may enhance FEV_1_ and QoL more than AN-SABA and LTRA.

**Supplementary Information:**

The online version contains supplementary material available at 10.1186/s12916-025-03847-z.

## Background

Inhaled corticosteroids (ICS) are currently considered the mainstay treatment for asthma. Historically, recommended treatment for mild asthma was as-needed (AN) with inhaled short-acting β2-agonists (SABA) prescribed on symptom presentation [1]. Nevertheless, frequent exacerbations and deaths have still been reported in approximately one-third of patients [2, 3]. Furthermore, overuse of SABA has also been associated with an increased risk of severe exacerbations and mortality [4], which could be reduced by between one-half and two-thirds in patients regularly treated with low-dose ICS [5]. Other treatment options include a combination of ICS and fast-acting β2-agonists (FABA), i.e., formoterol, as well as leukotriene-receptor antagonists (LTRA) [1, 6]. Theoretically, regular use is preferred to AN for better inflammation control, thus reducing exacerbations and mortality [1, 6]; nevertheless, nonadherence remains a barrier to adopting regular use, due to the infrequency of symptoms [7]. Evidence from several large randomized controlled trials (RCTs) has suggested that the AN-use of ICS in combination with FABA (AN-ICS/FABA) for mild asthma treatment could lower exacerbation rates, compared with AN-SABA alone [8–11]. Recent guidelines have classified asthma treatments based on the frequency of symptoms as follows: in children (aged 6–11 years) with asthma step 1 (symptoms < 2 times/month), AN-SABA or low-dose ICS taken whenever SABA is taken (AN-ICS); regular low-dose ICS is preferred and LTRA or AN-ICS as an option for step 2 (symptoms > 2 times/month) [1, 6]. In adolescents/adults, AN-ICS/FABA [1] or AN-ICS [6] is recommended for asthma step 1; regular low-dose ICS or AN-ICS/FABA are preferred [1], or LTRA [6] as an option for asthma step 2. Furthermore, a recent RCT comparing ICS and tiotropium in mild asthmatic adults with low sputum eosinophils showed comparable effects in terms of exacerbation prevention [12].

Four systematic reviews and meta-analyses (SRMAs) have compared the efficacy of AN-ICS/β2-agonists, regular ICS, and AN-SABA as mild asthma treatments [13–16]. In addition, two SRMAs compared AN-ICS and regular ICS for exacerbation prevention [17, 18], and five SRMAs have explored the efficacy of LTRA for exacerbation prevention and symptom improvement [19–23]. While evidence from these previous SRMAs is available, they are restricted to direct comparisons, failing to definitively rank the efficacy of different treatments among SABA, ICS, or ICS/FABA being used AN, and ICS, LTRA, ICS/LABA or tiotropium being used regularly. A recent systematic review and network meta-analysis (SRNMA) compared the efficacy of different inhaler therapies in preventing exacerbations in both mild and moderate adult asthma [24], subject to different treatments. As such, an unmet need exists for an updated SRNMA focusing on mild asthmatic patients stratified by age group (i.e., children and adolescents/adults). The optimal treatments for mild asthma across the various options require clarification to inform clinical practice. This study sought to identify variation across the treatment options and rank these based on short- to intermediate-term and long-term outcomes.

## Methods

This systematic review followed the Preferred Reporting Items for Systematic Reviews and Meta-analyses (PRISMA) extension statement for network meta-analysis (NMA) [25]. The protocol was registered in PROSPERO (CRD42022352384).

### Search strategy and study selection

Relevant studies were electronically searched in MEDLINE (PubMed) and Scopus from inception to March 31st, 2024, and ClinicalTrials.gov for a protocol, ongoing, and completed unpublished studies that met the eligibility criteria (Additional file 1: Table S1). Two reviewers independently screened titles and abstracts and assessed full-text articles for eligibility. RCTs in children (aged 6–11 years) or adolescents/adults with mild or intermittent asthma were included without language restrictions if they: (1) compared SABA, ICS, LTRA, ICS/FABA, ICS/LABA, or tiotropium; (2) had at least one of the following outcomes: exacerbations, asthma symptom scores, forced expiratory volume in 1 s (FEV_1_), asthma-specific quality of life (QoL), or adverse events (AEs). RCTs were excluded if they only included participants with exercise-induced asthma or viral-induced wheezing, baseline FEV_1_ < 80% predicted, compared different dosages or devices of the same treatment, or had insufficient data for pooling after three contact attempts with the authors. When multiple publications from the same study (with identical patients, authors, interventions, and outcomes) were identified, the most recent report was selected.

### Interventions of interest

The interventions of interest included the following drug classes as follows: SABA, ICS, LTRA, ICS/FABA, ICS/LABA, and tiotropium, with AN/prn or regular usage, any dosage, and any treatment duration. As per original individual studies, FABA was a fast-onset long-acting drug including formoterol, while SABA was a fast-onset short-acting drug including salbutamol or terbutaline, each of which if being used with ICS in a single or separate inhaler was considered similar as ICS/FABA class. On the other hand, a slow-onset long-acting drug including salmeterol was in LABA class. The inhaler type was not restricted.

### Outcomes of interest

The primary outcome was exacerbation, which was defined according to each RCT. Generally, exacerbations were classified as non-severe if worsening asthma symptoms led to more SABA use, and severe if systemic corticosteroids were used for ≥ 3 days or hospitalization. If severity was unspecified, a non-severe exacerbation was designated if the criteria of severe exacerbation were not met. The secondary outcomes comprised: (1) asthma symptoms and asthma control status assessed with the Asthma Control Questionnaire (ACQ), Asthma Control Test (ACT), or Pediatric-ACT (p-ACT); (2) FEV_1_, as a percentage of predicted value; (3) asthma-specific QoL assessed with the Asthma Quality of Life Questionnaire (AQLQ) or Pediatric Asthma QoL Questionnaire (PAQLQ); and (4) adverse events (AEs). Severe AEs (SAEs) were based on the definitions for each RCT included, otherwise the AEs were classified as non-severe. All outcomes were evaluated at the end of the study.

### Data extraction

Two reviewers independently extracted the following data: study settings, definition of mild asthma, patients’ characteristics (age, sex, smoking status, asthma step, previous exacerbation in the past year, previous ICS use, and FEV_1_), intervention details (name, dose, frequency, and duration of use), details of the outcome measures, and outcome data. Mild asthma was originally defined as per the individual studies (Additional file 1: Table S2), generally as follows: step 1 included symptoms or SABA use ≤ 2 times/month without nocturnal symptoms, used SABA alone, and no risk factors for exacerbations and no exacerbations in the previous year (1); step 2, symptoms or SABA use in the past month > 2 times but less than daily (1), being treated with low-dose ICS (< 400 mcg/day of budesonide or equivalent) (9). Any disagreements were discussed and a final consensus with an experienced advisor was made.

### Risk of bias assessment

Two reviewers independently assessed the risk of bias for the studies included using the Revised Cochrane risk-of-bias tool for randomized trials (RoB2), (Additional file 1: Table S3). We assessed all five domains of RoB2 and categorized the study as “low risk,” “some concern,” or “high risk” based on the overall assessment [26]. If at least one outcome in the study exhibited a high risk of bias, the entire study was considered a “high risk of bias.” Any disagreements were discussed and resolved within the author team.

### Data analysis and synthesis

The relative treatment effects on dichotomous exacerbations and adverse events were pooled using risk ratios (RR), continuous outcomes with similar scales (i.e., FEV_1_ and AQLQ) were pooled using mean differences (MD), and different scales (i.e., symptom score) were pooled using standardized mean differences (SMD). Formulas of conversion from median into mean and interquartile range into standard deviation (SD) and post-treatment means are shown in Additional file 1: Table S4.

Direct meta-analysis (DMA) was made when at least three studies were available for the same comparison. A random effect DerSimonian-Laird model was applied considering within- and between-studies variation due to methodological and clinical differences across studies [27]. Patient characteristics (i.e., age, sex, asthma step, prior ICS use, FEV_1,_ and previous asthma exacerbation in the past year) were considered as a potential source of heterogeneity by fitting each as a covariable in a meta-regression model; sources were confirmed if the model resulted in ≥ 50% reduction in an estimate of the between-study variance (τ^2)^ [28]. As such, exploratory subgroup analysis was performed if sufficient studies were available, otherwise, sensitivity analysis was conducted by excluding one study. Publication bias was assessed with funnel plots.

In addition, a two-stage NMA was performed as follows: First, binomial and linear regression analyses were performed on dichotomous and continuous outcomes, respectively, to estimate relative treatment effects. Second, multivariate random-effect meta-analysis was applied to pool the relative treatment effects across individual studies. Then, multiple treatment comparisons were made for each pair of interventions [29]. Third, interventions were ranked based on the surface under the cumulative ranking curve (SUCRA) method [30]. The NMA transitivity assumption was visually assessed across intervention comparisons on the covariates that were prespecified as possible modifiers of exacerbation outcome: mean age, percentage of female sex, mean % predicted FEV_1_, percentage of baseline low-dose ICS use, and percentage of previous exacerbation within the past year. Assessment of transitivity for treatment adherence was planned but this was rarely reported in the included studies. The consistency assumption was assessed using a design-by-treatment interaction model [31, 32], and potential publication bias by comparison-adjusted funnel plots and Egger’s test [33]. All analyses were performed using Stata 18, stratified by age group (i.e., children aged 6–11 years and adolescents/adults). A two-sided *p*-value of 0.05 was used as the threshold for statistical significance, except for the heterogeneity assessment where the Cochrane Q test *p*-value of 0.1 was used. The certainty of the evidence generated from the NMA was assessed using the Confidence in Network Meta-Analysis (CINeMA) framework [34].

## Results

**Fig. 1:**
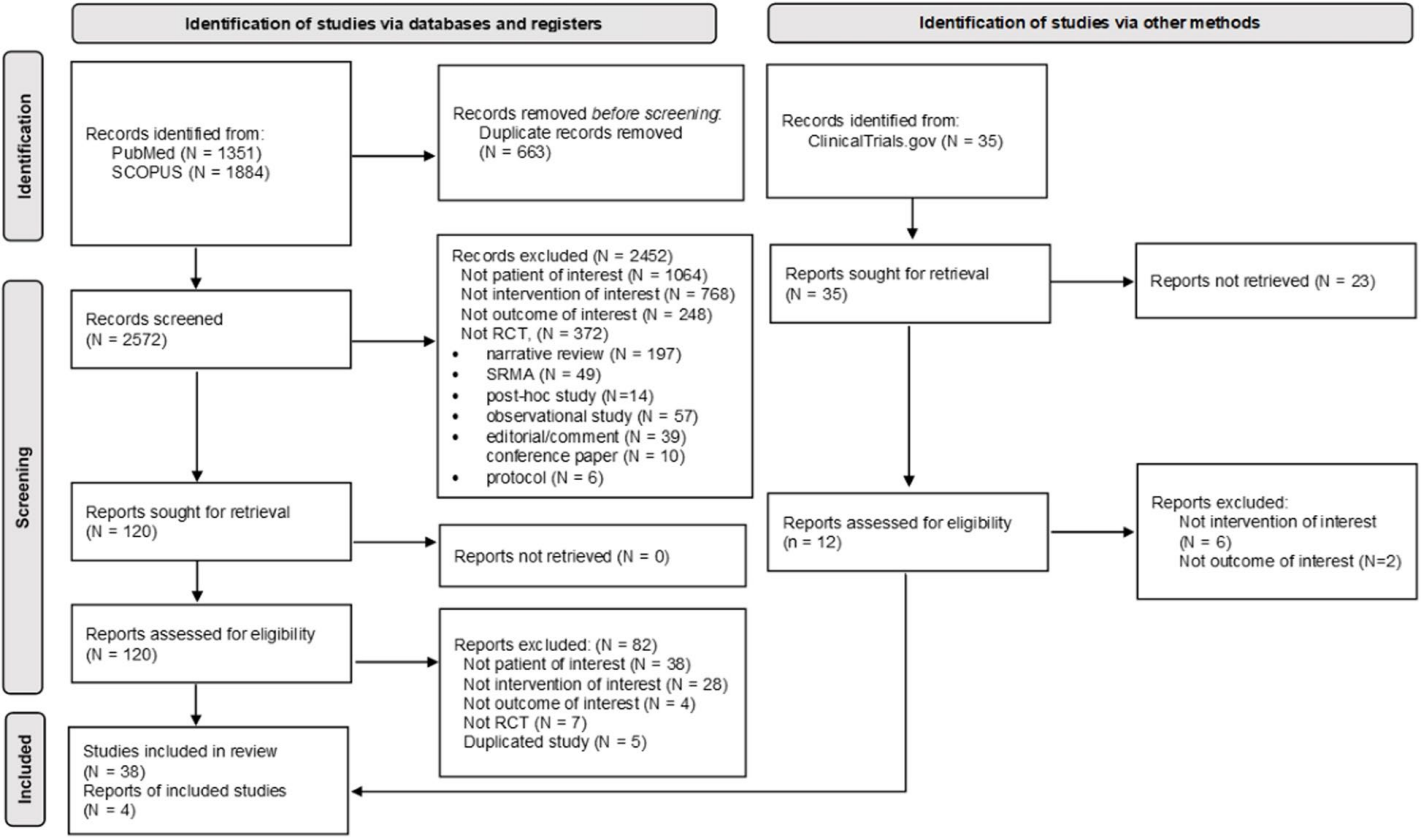
PRISMA 2020 flow diagram

A total of 3270 articles were identified; titles and abstracts of 2572 records were screened after removing duplicates, and 42 were considered eligible for inclusion (Fig. 1). The characteristics of the eligible 42 studies are in Additional file 1: Table S5. Of these, 13 studies were conducted in children, and 29 in adolescents/adults with sample sizes ranging from 27 to 1974 and 27 to 1945 patients, respectively. The years of publication ranged from 1991 to 2022. The mean age ranged from 5.5 to 10.7 in children and 26.6 to 71.0 in adolescents/adults. The mean baseline FEV_1_ of these groups ranged from 83.5 to 102.0% and 84.2 to 101.0%, respectively. A total of five (i.e., AN-SABA, AN-ICS, regular ICS, LTRA, and regular ICS/SABA) and six treatments (i.e., AN-SABA, regular ICS, LTRA, AN-ICS/FABA, regular ICS/LABA, and tiotropium) were studied in children and adolescents/adults, respectively.

### Risk of bias

Of the studies included, 21.4% were classified as low risk of bias, 59.5% as some concern, and 19% as high risk of bias, mainly regarding issues associated with the randomization process, deviation from intended intervention, or selection of results reported (Additional file 1: Fig. S1).

#### Findings in children

##### Non-severe exacerbation

A DMA suggested that regular ICS significantly reduced non-severe exacerbations compared with LTRA, with a RR (95% CI) of 0.63 (0.49, 0.82), but not relative to AN-ICS, with a RR (95% CI) of 0.83 (0.61, 1.12) (Additional file 1: Fig. S2).

**Fig. 2:**
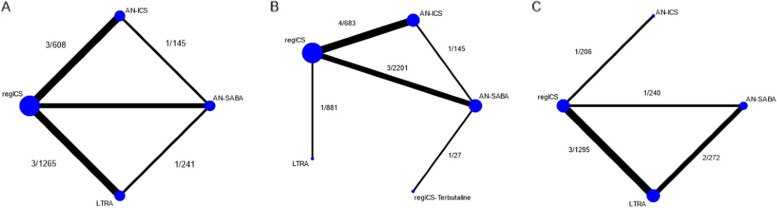
Network map of studies in children. **A** Non-severe exacerbation, **B** FEV_1_ percentage of predicted value, **C** adverse-events. The links between the nodes indicate the direct comparisons between pairs of treatments, and the thickness of the lines is proportional to the number of comparisons between each treatment. The numbers of studies and participants are shown

Six RCTs [35–40] involving 2716 patients were included in a NMA of interventions, including AN-SABA, AN-ICS, regular ICS, and LTRA (Fig. 2A). Compared with AN-SABA, regular ICS, and LTRA were significantly better in preventing non-severe exacerbations, with RRs (95% CI) of 0.61 (0.48, 0.78), and 0.76 (0.58, 0.99), respectively. While AN-ICS showed a similar trend, it did not significantly differ from AN-SABA, with a RR (95% CI) of 0.74 (0.53, 1.02) (Table 1). The ranking by SUCRA indicated that regular ICS had the highest probability of lowering the risk of non-severe exacerbations, followed by AN-ICS, LTRA, and AN-SABA (Additional file: Table S6). There were no concerns on the mean % predicted FEV_1_, percentage of baseline low-dose ICS use, mean baseline ACT score, and percentage of previous exacerbation in the past year across treatment comparisons: however, there were some concerns on the mean age and percentage of the female sex (Additional file 1: Table S7). No evidence of inconsistency existed in the network (chi-square = 0.5, *p*-value = 0.919). The comparison-adjusted funnel plot showed no asymmetry (Egger’s test *p*-value = 0.272).

**Table 1:** Treatment effects of ICS-containing regimens, LTRA, and tiotropium relative to AN-SABA on exacerbation and adverse events

Treatment	Non-severe exacerbation			Severe exacerbation			Non-severe adverse events			Severe adverse events		
	Children	Certainty	Adults	Certainty	Adults	Certainty	Children	Certainty	Adults	Certainty	Adults	Certainty
**AN-ICS**	0.74 (0.53,1.02)	Moderate	NA	NA	NA	NA	0.59 (0.14,2.57)	NA	NA	NA	NA	NA
**Regular ICS**	0.61 (0.48,0.78)*	High	0.50 (0.33,0.76)*	Moderate	0.58 (0.46,0.73)*	Low	0.88 (0.45,1.72)	High	1.01 (0.86,1.18)	High	0.65 (0.45,0.94)*	High
**LTRA**	0.76 (0.58,0.99)*	Moderate	0.64 (0.31,1.35)	Low	NA	NA	1.08 (0.56,2.08)	Low	NA	NA	NA	NA
**AN-ICS/FABA**	NA	NA	0.46 (0.28,0.75)*	Moderate	0.42 (0.30,0.58)*	High	NA	NA	0.97 (0.82,1.15)	High	0.79 (0.52,1.21)	Moderate
**Regular ICS/LABA**	NA	NA	0.41 (0.24,0.71)*	Moderate	0.39 (0.27,0.56)	Moderate	NA	NA	1.01 (0.81,1.27)	Moderate	0.57 (0.31,1.02)	Low
**Tiotropium**	NA	NA	0.44 (0.21,0.94)*	Low	0.51 (0.13,1.91)	Very low	NA	NA	NA	Very low	NA	NA

Treatment effects (relative to AN-SABA) are represented by a RR (95% CI). * indicates statistical significance. Abbreviation: *NA*, no data for analysis

##### Percentage of predicted FEV_1_

A DMA showed that regular ICS increased FEV_1_ to a greater extent than AN-SABA, but was not different from AN-ICS, with MDs (95% CI) of 4.30 (0.35, 8.25), and 1.51 (− 0.34, 3.37), respectively (Additional file 1: Fig. S3).

Eight RCTs [36–38, 41–44] involving 3937 patients were included in a NMA of interventions that included AN-SABA, AN-ICS, regular ICS, LTRA, and regular ICS/SABA (Fig. 2B). Regular ICS resulted in higher FEV_1_ compared to AN-SABA, with an MD (95% CI) of 4.12 (0.74, 7.50), but did not differ significantly from AN-ICS, LTRA, or regular ICS/SABA (Table 2). The ranking by SUCRA indicated that regular ICS had the highest probability of increasing FEV_1_, followed by regular ICS/SABA, AN-ICS, LTRA, and AN-SABA (Additional file 1: Table S8). There was no evidence of inconsistency in the network (chi-square = 0.35, *p*-value = 0.839).

**Table 2:** Treatment effects of ICS-containing regimens, LTRA, and tiotropium relative to AN-SABA on asthma symptoms, asthma-specific quality-of-life, and FEV_1_

Treatment	Asthma symptom score		AQLQ score^b^		% predicted FEV_1_^b^			
	Adults	Certainty	Adults	Certainty	Children	Certainty	Adults	Certainty
**AN-ICS**	NA	NA	NA	NA	1.91 (− 2.23,6.05)	Low	NA	NA
**Regular ICS**	− 0.33 (− 0.90,0.24)	Very low	0.21 (0.07,0.35)*	Moderate	4.12 (0.74,7.50)*	Very low	3.53 (1.68,5.38)*	Low
**LTRA**	− 0.94 (− 1.93,0.04)	Low	− 0.03 (− 0.19,0.13)	Moderate	0.92 (− 5.65,7.49)	Very low	− 0.20 (− 3.64,3.25)	Very low
**AN-ICS/FABA**	− 0.48 (− 1.49,0.54)	Low	NA	NA	NA	NA	4.45 (1.76,7.15)*	Low
**Regular ICS/LABA**	− 0.41 (− 1.17,0.35)	Very low	0.12 (− 0.04,0.28)	Moderate	3.10** (− 8.32,14.52)	Very low	3.45 (0.40,6.49)*	Low
**Tiotropium**	NA	NA	NA	NA	NA	NA	NA	NA

Treatment effects (relative to AN-SABA) are represented by ^a^SMD (95% CI) and ^b^MD (95% CI). * indicates statistical significance. ** based on one RCT on AN-SABA vs. regular ICS/terbutaline. Abbreviation: *NA*, no data for analysis

The comparison-adjusted funnel plot showed asymmetry (Additional file 1: Fig. S4), based on the comparisons between regular ICS vs. AN-SABA [42], and regular ICS vs. AN-ICS [36] for the patients that had a mean baseline FEV_1_ < 90%predicted in the former, and a higher proportion of asthma step 1 and 2 patients with prior ICS use in the latter. A contour-enhanced funnel plot suggested that the asymmetry was attributable to heterogeneity in the baseline FEV_1_ and asthma step in patients included. A sensitivity analysis that excluded one of the two RCTs at a time produced rank orders similar to the main analysis.

##### Non-severe adverse events

Five RCTs [35, 37, 39, 40, 45] involving 1,649 patients were included in a NMA of the following interventions: AN-SABA, AN-ICS, regular ICS, and LTRA (Fig. 2C). No differences between ICS, LTRA, and AN-SABA were noted (Table 1). The probability of the lowest risk of AE was with AN-ICS, followed by regular ICS, AN-SABA, and LTRA (Additional file 1: Table S9). There was no evidence of inconsistency in the network (chi-square = 1.83, *p*-value = 0.401). The comparison-adjusted funnel plot showed no asymmetry (Egger’s test *p*-value = 0.127).

#### Findings in adolescents/adults

##### Non-severe exacerbation

A DMA found that regular ICS significantly reduced non-severe exacerbations, with a RR (95% CI) of 0.44 (0.32, 0.61) relative to AN-SABA, but not relative to LTRA or regular ICS/LABA, with RRs (95% CI) of 1.18 (0.82, 1.70), and 0.81 (0.40, 1.62), respectively (Additional file 1: Fig. S5).

**Fig. 3:**
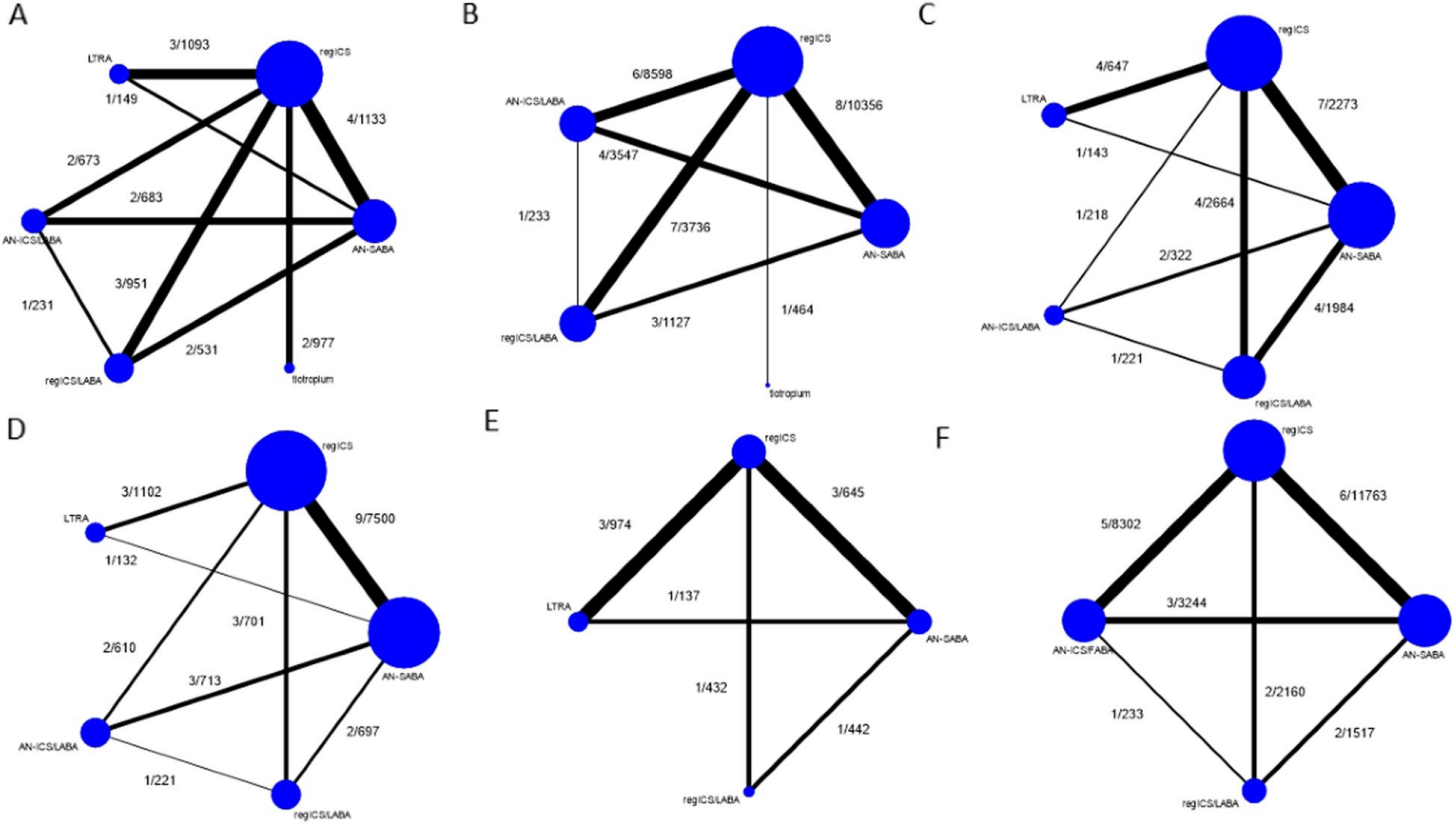
Network map of studies in adolescents/adults. **A** Non-severe exacerbation, **B** severe exacerbation, **C** symptom score, **D** FEV_1_ percentage of predicted value, **E** asthma-specific quality-of-life, and **F** severe adverse events. The links between the nodes indicate the direct comparisons between pairs of treatments, and the thickness of the lines is proportional to the number of comparisons between each treatment. The numbers of studies and participants are shown

Eight RCTs [9, 12, 46–51] and one unpublished RCT (NCT1316380) involving 6,421 patients were included in a NMA of the following interventions: AN-SABA, regular ICS, LTRA, AN-ICS/FABA, regular ICS/LAB, and tiotropium (Fig. 3A). The ICS-containing regimens, including ICS alone, AN-ICS/FABA, and regular ICS/LABA, were superior to AN-SABA, with RRs (95% CI) of 0.50 (0.33, 0.76), 0.46 (0.28, 0.75), and 0.41 (0.24, 0.71), respectively. With varying degrees of precision, tiotropium outperformed AN-SABA in terms of lowering non-severe exacerbation, with a RR (95% CI) of 0.44 (0.21, 0.94), but it was not different from ICS-containing regimens; there was no appreciable between-study network heterogeneity (τ^2^ = 0.05) (Table 1). The ranking by SUCRA indicated that regular ICS/LABA had the highest probability of lowering the risk of non-severe exacerbations, followed by tiotropium, AN-ICS/FABA, regular ICS, LTRA, and AN-SABA (Additional file 1: Table S10). There were no important concerns on patient characteristics regarding mean age, percentage of the female sex, and mean % predicted FEV_1_ across treatment comparisons, except for the percentage of baseline low-dose ICS use, and percentage of previous exacerbation in the past year (Additional file 1: Table S11). There was no evidence of inconsistency in the network (chi-square = 9.82, *p*-value = 0.199). The comparison-adjusted funnel plot showed no asymmetry (Egger’s test *p*-value = 0.690).

##### Severe exacerbation

A DMA suggested that regular ICS significantly reduced severe exacerbations when compared with AN-SABA, with a RR (95% CI) of 0.61 (0.47, 0.80); AN-ICS/FABA and regular ICS/LABA were superior to ICS alone, with RRs (95% CI) of 0.74 (0.56, 0.98), and 0.65 (0.50, 0.84), respectively (Additional file 1: Fig. S6). Exploratory subgroup analyses based on baseline FEV_1_ and ACQ-5 are presented in Additional file 1: Fig. S7–S8.

Thirteen RCTs [8–11, 49, 50, 52–58], and two unpublished RCTs (NCT1316380 and NCT455923) involving 27,828 patients were included in a NMA for the following interventions: AN-SABA, regular ICS, AN-ICS/FABA, regular ICS/LABA, and tiotropium (Fig. 3B). The ICS-containing regimens, including ICS alone, AN-ICS/FABA, and regular ICS/LABA, were less likely to lead to severe exacerbations compared to AN-SABA, with RRs (95% CI) of 0.58 (0.46, 0.73), 0.42 (0.30, 0.58), and 0.39 (0.27, 0.56), respectively. With varying degrees of precision, AN-ICS/FABA and regular ICS/LABA were superior to regular ICS, while AN-ICS/FABA and regular ICS/LABA were not different from each other, with no appreciable between-study network heterogeneity (τ^2^ = 0.06) (Table 1). Of note, tiotropium was not superior to AN-SABA in reducing severe exacerbation risk, with a RR (95% CI) of 0.51 (0.13, 1.91). According to the SUCRA, regular ICS/LABA had the highest probability of reducing severe exacerbation risk, followed by AN-ICS/FABA, tiotropium, regular ICS, and AN-SABA (Additional file 1: Table S12). There were no important concerns on the following patient characteristics regarding mean age, and percentage of female sex across treatment comparisons, except for the mean % predicted FEV_1_, percentage of baseline low-dose ICS use, and percentage of previous exacerbation in the past year (Additional file 1: Table S13). There was no evidence of inconsistency in the network (chi-square = 3.89, *p*-value = 0.793).

The comparison-adjusted funnel plot showed asymmetry associated with two treatment comparisons, AN-ICS/SABA vs. AN-SABA and AN-ICS/SABA vs. regular ICS, from one RCT [49] (Additional file 1: Fig. S9). A contour-enhanced funnel plot suggested the asymmetry was attributable to heterogeneity within the RCT which enrolled patients who had achieved asthma control following low-dose ICS prescription during the run-in period. A sensitivity analysis that excluded this RCT produced a rank order parallel to the main analysis.

##### Asthma symptoms

A DMA indicated that a significant symptom reduction was associated with regular ICS use compared to AN-SABA, with a SMD (95% CI) of − 0.44 (− 0.68, − 0.21), but not relative to LTRA, with a SMD (95% CI) of − 0.83 (− 2.17, 0.51). Treatment with AN-ICS/FABA and regular ICS resulted in no difference in change of ACQ-5 score, with an MD (95% CI) of 0.08 (0, 0.17), (Additional file 1: Fig. S10).

Eleven RCTs [47, 49, 51, 52, 56, 58–63] involving 8,392 patients were included in a NMA of the following interventions: AN-SABA, regular ICS, LTRA, AN-ICS/FABA, and regular ICS/LABA (Fig. 3C). None of the interventions showed a difference in post-treatment asthma symptoms (Table 2). According to the SUCRA, the highest probability for symptom reduction was associated with LTRA, followed by AN-ICS/FABA, regular ICS/LABA, regular ICS, and AN-SABA (Additional file 1: Table S14). There was no evidence of inconsistency in the network (chi-square = 3.17, *p*-value = 0.869).

The comparison-adjusted funnel plot showed an asymmetry associated with the comparison of regular ICS vs. LTRA in a single RCT [63] (Additional file 1: Fig. S11). A contour-enhanced funnel plot suggested that the asymmetry was attributable to the RCT that included patients with a very high mean baseline sputum eosinophil of 13%. A sensitivity analysis that excluded this RCT resulted in a different rank order from the main analysis with AN-ICS/FABA becoming top-ranked, followed by regular ICS/LABA, regular ICS, LTRA, and AN-SABA (Additional file 1: Table S15).

##### Percentage of predicted FEV_1_

A DMA suggested that regular ICS significantly increased %predicted FEV_1_ relative to AN-SABA and LTRA, with MDs (95% CI) of 3.62 (2.10, 5.14), and 3.96 (0.30, 7.61), respectively (Additional file 1: Fig. S12). AN-ICS/FABA significantly increased %predicted FEV_1_ more than AN-SABA, with an MD (95% CI) of 3.70 (2.96, 4.44), albeit this did not differ significantly from regular ICS (Additional file 1: Fig. S12). Results from the exploratory subgroup analyses according to the heterogeneity in asthma step and ICS use at baseline are shown in Additional file 1: Fig. S13–S14.

Thirteen RCTs [9, 47–49, 51, 53, 54, 57, 59–61, 64, 65] involving 11,676 patients were included in a NMA of interventions that included AN-SABA, regular ICS, LTRA, AN-ICS/FABA, and regular ICS/LABA (Fig. 3D). ICS-containing regimens were superior to AN-SABA for increasing FEV_1_ (Table 2). Compared with LTRA, regular ICS and AN-ICS/FABA were better in terms of FEV_1_. Ranking by SUCRA suggested that AN-ICS/FABA had the highest probability of increasing FEV_1,_ followed by regular ICS, regular ICS/LABA, LTRA, and AN-SABA (Additional file 1: Table S16). No evidence of inconsistency existed in the network (chi-square = 4.35, *p*-value = 0.887).

The comparison-adjusted funnel plot showed asymmetry, due to the comparisons between regular ICS vs. AN-SABA [57], and regular ICS vs. regular ICS/LABA [65] (Additional file 1: Fig. S15). Contour-enhanced funnel plots suggested that the asymmetry was attributable to differences in asthma step and baseline ICS use. After excluding each RCT one at a time in a sensitivity analysis, the rank orders remained similar to the main analysis.

##### Asthma-specific quality-of-life

A DMA suggested that LTRA was associated with a significantly worse AQLQ score compared to regular ICS, with an MD (95% CI) of − 0.22 (− 0.34, − 0.11, (Additional file 1: Fig. S16). Although the effect of regular ICS and AN-SABA on AQLQ was not different, with an MD (95% CI) of − 0.13 (− 0.30, 0.03), variation in baseline age and %predicted FEV_1_ was present. Use of regular ICS in patients aged < 35 years or had baseline FEV_1_ ≥ 90% predicted did not lead to improved QoL compared to AN-SABA in an exploratory subgroup analysis (Additional file 1: Fig. S17–S18).

Five RCTs [47, 48, 51, 56, 64] involving 2630 patients were included in a NMA of the following interventions: AN-SABA, regular ICS, LTRA, and regular ICS/LABA (Fig. 3E). Treatment with regular ICS was associated with significantly better QoL than AN-SABA and LTRA. Regular ICS ranked best for QoL, followed by regular ICS/LABA, AN-SABA, and LTRA (Additional file 1: Table S17). There was no evidence of inconsistency in the network (chi-square = 3.92, *p*-value = 0.270).

The comparison-adjusted funnel plot showed an asymmetry, due to the comparison of regular ICS vs. AN-SABA from a single RCT [64] that included only asthma step 1 patients (Additional file 1: Fig. S19). A contour-enhanced funnel plot suggested missing studies in the non-significant area contributing to potential publication bias. A significant Egger’s test suggested a small study effect existed (*p*-value = 0.023). Excluding this study in a sensitivity analysis produced a similar rank order to the main analysis.

##### Severe adverse events

Eight RCTs [8–11, 49, 52, 54, 61] involving 27,219 patients were included in a NMA of the following interventions: AN-SABA, regular ICS, AN-ICS/FABA, and regular ICS/LABA (Fig. 3F). Regular ICS was associated with a significantly lower risk of SAE than AN-SABA, with a RR (95% CI) of 0.65 (0.45, 0.94). The lowest risk of SAE was with regular ICS/LABA, followed by regular ICS, AN-ICS/FABA, and AN-SABA (Additional file 1: Table S18). There was no evidence of inconsistency in the network (chi-square = 3.06, *p*-value = 0.801). The comparison-adjusted funnel plot showed no asymmetry (Egger’s test *p*-value = 0.209). A result of DMA is presented in Additional file 1: Fig. S20.

The ratings of confidences in NMA between each treatment comparison are presented in Additional file 1: Table S19–S20. The extracted data for analysis and the PRISMA Checklist are presented in Additional file 2 and Additional file 3.

#### Transitivity assumption

The transitivity assumption was assessed for exacerbation and severe exacerbation outcomes in children and adolescent/adult studies. All children had asthma step 2. The patient characteristics, including mean age, percentage of the female sex, mean % predicted FEV_1_, percentage of baseline low-dose ICS use, mean baseline ACT score, and percentage of previous exacerbation in the past year were described for each treatment comparison: regular ICS vs. SABA, regular ICS vs. AN-ICS, LTRA vs. SABA, and LTRA vs. regular ICS (Additional file 1: Table S7). While the mean % predicted FEV_1_, percentage of baseline low-dose ICS use, mean baseline ACT score, and percentage of previous exacerbation in the past year were comparable across individual comparisons, the mean age and percentage of the female sex were different. Therefore, the transitivity assumption may not hold for comparing LTRA vs. SABA and LTRA vs. regular ICS on exacerbation outcome.

In adolescents/adults, the patient characteristics for exacerbation outcome including mean age, percentage of the female sex, mean % predicted FEV_1_, and percentage of previous exacerbation in the past year appeared comparable across treatment comparisons, except the percentage of baseline low-dose ICS use (Additional file 1: Table S11). Likewise, for severe exacerbation outcome, the mean % predicted FEV_1_, percentage of baseline ICS use, and percentage of previous exacerbation in the past year were different among the comparisons of regular ICS/LABA vs. regular ICS, and tiotropium vs. regular ICS (Additional file 1: Table S13). This may violate the transitivity assumption and invalidate treatment effects on exacerbation outcomes; hence this result should be viewed with caution.

## Discussion

This SRNMA showed that treating mild pediatric asthma with regular ICS was superior to AN-SABA in preventing exacerbations while treating with AN-ICS and LTRA was marginally better than AN-SABA. Likewise, in adolescents/adults, ICS-containing regimens are superior to AN-SABA for lowering exacerbations of all severities, and to a greater extent in combination with LABA as regular ICS/LABA or as-needed ICS/FABA than in regular ICS alone. Moreover, regular ICS use increased FEV_1_ more than AN-SABA in children, and even better than AN-SABA and LTRA in adolescents/adults.

In children, we confirmed the superiority of ICS over LTRA in lowering the risk of exacerbations; this was consistent with a previous SRMA on ICS vs. LTRA [21], and similar to another previous SRMA, where LTRA were reported to be non-inferior to ICS in terms of FEV_1_ and asthma symptoms [66]. Nevertheless, the latter SRMA recommended ICS over LTRA in patients with low FEV_1_ at baseline. In our SRNMA focusing on mild asthma, AN-ICS were marginally, albeit non-significantly, better than AN-SABA in preventing non-severe exacerbations, but were still ranked lower than regular ICS as a preferred treatment.

In adolescents/adults, the highest- to lowest-ranked interventions for preventing non-severe exacerbations were regular ICS/LABA, tiotropium, AN-ICS/FABA, regular ICS, and LTRA. Although regular ICS/LABA were ranked highest, the upper limits of the RR 95% CI for the three ICS-containing regimens were not different. Therefore, regular ICS monotherapy could be considered non-inferior to ICS/FABA or ICS/LABA in preventing non-severe exacerbations with all providing at least a 25% lowered risk relative to AN-SABA. Regarding our findings related to severe exacerbations, AN-ICS/FABA ranked higher than regular ICS, and attenuated severe exacerbation risk by 27% (RR 0.73 [0.54, 0.97]), consistent with previous SRMAs [14, 15]. However, from our exploratory subgroup DMA, the superiority of AN-ICS/FABA over regular ICS in preventing severe exacerbation was greater in patients with FEV_1_ > 88%predicted (RR 0.39 [0.23, 0.67]) and lower ACQ-5 scores (RR 0.56 [0.37, 0.85]). Therefore, AN-ICS/FABA may be more suitable for patients without impairment of FEV_1_ or poor asthma control. Of note, although this SRNMA showed a non-significant difference between tiotropium and ICS-containing regimens on reducing exacerbation in mild asthma, this result in mild asthma may not be generalizable due to several reasons: (1) sputum eosinophil count is not routinely performed in mild asthma as in Lazarus’s study, (2) LAMA without ICS may not be safely used in mild asthmatic patients in whom eosinophilic inflammation is unknown, and (3) the percentage of baseline low-dose ICS use was high in the comparison between tiotropium and regular ICS, as in NCT1316360, in which tiotropium was used on-top of low-dose ICS. This may challenge the reliability of the transitivity assumption and make indirect comparisons on exacerbation outcomes among tiotropium vs. other treatment comparisons in the network (LTRA vs. regular ICS, and AN-ICS/FABA vs. regular ICS) unreliable.

Regarding our findings on asthma symptoms, we speculate that symptom-driven use of AN-ICS/FABA provides the highest probability for symptom reduction. However, similar to previous SRMA [67], asthma treatments did not show clear differences, suggesting any of these may be adopted for symptom relief.

The greater effects of the combination of ICS and FABA or LABA on increasing FEV_1_ may be attributed to the long-acting bronchodilator effects exerted by the regimens. Although AN-ICS/FABA ranked best in terms of improved FEV_1_, followed by regular ICS/LABA, the overlapping 95% CI upper limits for the MDs of the ICS-containing regimens, suggested comparable FEV_1_ improvements for ICS-containing regimens. LTRA consistently ranked lowest and was not different from AN-SABA, and may not be a good option for mild asthma patients with low FEV_1_.

Given exacerbations are less likely in mild asthma patients, QoL indicators may represent an alternative measure as a greater number and severity of exacerbations were previously reported to be associated with impaired QoL in a large cohort study [68]. In our SRNMA, regular ICS was top-ranked for optimizing QoL. However, due to a lack of QoL assessment as an outcome measure particularly in studies of mild pediatric asthma and for some interventions, such as ICS/FABA and ICS/LABA combination therapies, an updated review focused on QoL outcomes is warranted, when more data become available.

Our findings were not always consistent with previous SRNMAs [69, 70]. Overall, we included patients with less severe disease, therefore the treatment effects reported are specific to those with “mild asthma.” The exploratory subgroup analyses using DMA approaches to resolve issues around the use of AN-ICS/FABA or regular ICS in mild asthma is a particular strength of our SRNMA given its lack of sufficient consideration in the current asthma treatment guidelines.

Nevertheless, potential limitations exist within our study. First, the classification of asthma steps was not consistent between older and more recent studies where terms such as “intermittent” and “mild persistent” asthma used in the older studies do not directly correspond to asthma “step 1” and “step 2” from more recent studies. To overcome this issue, the definition of asthma step from the recent studies was strictly applied to the older studies. Second, there is a lack of RCTs in children with asthma step 1 and treatments with AN-ICS/FABA or regular ICS/LABA, undermining treatment recommendations with AN-SABA or AN-ICS or regular ICS in mild pediatric asthma (step 1), or for treatment with AN-ICS/FABA in mild pediatric asthma (steps 1 and 2). Furthermore, given the lack of RCTs for AN-ICS treatment in adolescents/adults, further evaluation of these options is warranted. Third, the transitivity assumption may be violated potentially leading to invalid treatment effect estimates, particularly in the comparisons of LTRA vs. AN-SABA and LTRA vs. regular ICS in children; regular ICS LABA vs. regular ICS and tiotropium vs. regular ICS in adolescents/adults. Therefore, the evidence synthesis from our NMA for reducing exacerbations should be interpreted cautiously among such treatment comparisons. Instead, our results may provide additional evidence for the treatments stated in the current asthma guidelines for mild asthma: AN-SABA, AN-ICS/FABA, or regular ICS, and rank as the most effective treatment among them. Lastly, treatment adherence was not considered in our SRNMA given the scarcity of reported data. Therefore, an updated review is warranted when additional data becomes available.

## Conclusions

The evidence from this systematic review in mild asthma suggests that in children (aged 6–11 years), regular ICS might be the optimal treatment for exacerbation prevention and increasing FEV_1_. In adolescents/adults, ICS-containing regimens are superior to AN-SABA for lowering exacerbations of all severities, with a greater extent in combination with FABA (AN-ICS/FABA) than in regular ICS alone for attenuating severe exacerbations. However, AN-ICS/FABA may not be suitable for patients with low FEV_1_. Additionally, regular use of ICS may enhance FEV_1_ and QoL more than AN-SABA and LTRA.

## Supplementary Information

Additional file 1. FigS1 − [risk of bias]. FigS2 − [children non-severe exacerbation]. FigS3 − [children %predicted FEV1]. FigS4– [children publication bias FEV1]. FigS5 − [adult non-severe exacerbation]. FigS6– [adult severe exacerbation]. FigS7– [adult subgroup severe exacerbation by FEV1]. FigS8– [adult subgroup severe exacerbation by ACQ]. FigS9– [adult publication bias severe exacerbation]. FigS10– [adult asthma symptom]. FigS11– [adult publication bias asthma symptom]. FigS12– [adult FEV1]. FigS13– [adult subgroup FEV1 by asthma step]. FigS14– [adult subgroup FEV1 by baseline ICS use]. FigS15– [adult publication bias FEV1]. FigS16– [adult asthma-specific quality-of-life]. FigS17– [adult subgroup asthma-specific quality-of-life by age]. FigS18– [adult subgroup asthma-specific quality-of-life by FEV1]. FigS19– [adult publication bias asthma-specific quality-of-life]. FigS20– [adult severe adverse events].

Additional file 2. The extracted data for analysis.

Additional file 3. PRISMA Checklist.

## Data Availability

Extracted data is available as a supplementary file.

## Abbreviations

ACQ: Asthma Control Questionnaire
ACT: Asthma Control Test
AQLQ: Asthma Quality of Life Questionnaire
AEs: Adverse events
AN: As needed, prn
CINeMA: Confidence in Network Meta-Analysis
DMA: Direct meta-analysis
FABA: Fast-acting β2-agonists
FEV_1_: Forced expiratory volume in 1 s
ICS: Inhaled corticosteroids
ICS/FABA: Inhaled corticosteroids combined with fast-acting β2-agonists
ICS/LABA: Inhaled corticosteroids combined with long-acting β2-agonists
LABA: Long-acting β2-agonists
LTRA: Leukotriene-receptor antagonists
MD: Mean difference
NMA: Network meta-analysis
QoL: Quality of life
RCT: Randomized control trial
ROB: Risk of bias
RR: Risk ratio
SABA: Short-acting β2-agonists
SAEs: Severe adverse events
SMD: Standardized mean difference
SRMA: Systematic review and meta-analysis
SRNMA: Systematic review and network meta-analysis
SUCRA: Surface under the cumulative ranking curve
