# Supplementary material for: Regular versus as-needed treatments for mild asthma in children, adolescents, and adults: a systematic review and network meta-analysis
Source: BMC Med. 2025 Jan 21;23:21. doi: 10.1186/s12916-025-03847-z (PMC11752773; doi:10.1186/s12916-025-03847-z)
Supplement: Supplementary file 1 — Additional file 1. FigS1 − [risk of bias]. FigS2 − [children non-severe exacerbation]. FigS3 − [children %predicted FEV1]. FigS4– [children publication bias FEV1]. FigS5 − [adult non-severe exacerbation]. FigS6– [adult severe exacerbation]. FigS7– [adult subgroup severe exacerbation by FEV1]. FigS8– [adult subgroup severe exacerbation by ACQ]. FigS9– [adult publication bias severe exacerbation]. FigS10– [adult asthma symptom]. FigS11– [adult publication bias asthma symptom]. FigS12– [adult FEV1]. FigS13– [adult subgroup FEV1 by asthma step]. FigS14– [adult subgroup FEV1 by baseline ICS use]. FigS15– [adult publication bias FEV1]. FigS16– [adult asthma-specific quality-of-life]. FigS17– [adult subgroup asthma-specific quality-of-life by age]. FigS18– [adult subgroup asthma-specific quality-of-life by FEV1]. FigS19– [adult publication bias asthma-specific quality-of-life]. FigS20– [adult severe adverse events]. [file 12916_2025_3847_MOESM1_ESM.docx]

**Additional file 1**

**Tables**

**Table S1.** Search terms and search strategies

**Table S2.** Definition of mild asthma in the included RCTs

**Table S3**. Risk of bias of eligible studies

**Table S4.** Data synthesis: Formulas for data conversion

**Table S5.** Study characteristics of included RCTs

**Network meta-analysis in children (aged 6−11 years)**

**Table S6.** Treatment effects for non-severe exacerbation

**Table S7.** Transitivity table for non-severe exacerbation outcome

**Table S8**. Treatment effects for %predicted FEV_1_

**Table S9.** Adverse events

**Network meta-analysis in adolescents/adults**

**Table S10.** Treatment effects for non-severe exacerbation

**Table S11.** Transitivity table for non-severe exacerbation outcome

**Table S12.** Treatment effects for severe exacerbation

**Table S13**. Transitivity table for severe exacerbation outcome

**Table S14.** Treatment effects for asthma symptom outcome

**Table S15.** Sensitivity analysis on treatment effects for asthma symptom outcome

**Table S16.** Treatment effects for %predicted FEV_1_

**Table S17.** Treatment effects for asthma-specific quality-of-life

**Table S18.** Severe adverse events

**Grading the evidence of the network meta-analysis using CINeMA**

**Table S19.** Rating of confidence in NMA in children

**Table S20.** Rating of confidence in NMA in adolescents/adults

**Figures**

**Fig. S1.** Risk of bias assessment according to the Revised Cochrane risk-of-bias tool for randomized trials (RoB2) algorithm

**Direct meta-analysis in children (aged 6−11 years)**

**Fig. S2.** Forest plots of non-severe exacerbation outcome

**Fig. S3.** Forest plots of % predicted FEV_1_ outcome

**Fig. S4.** Publication bias assessments for all relative treatment comparisons on FEV_1_ outcome

**Direct meta-analysis in adolescents/adults**

**Fig. S5.** Forest plots of non-severe exacerbation outcome

**Fig. S6.** Forest plots of severe exacerbation outcome

**Fig. S7.** Subgroup analysis by baseline % predicted FEV_1_ on severe exacerbation outcome

**Fig. S8.** Subgroup analysis by baseline ACQ-5 and proportion of previous exacerbation in the past year on severe exacerbation outcome

**Fig. S9.** Publication bias assessments for all relative treatment comparisons on severe exacerbation outcome

**Fig. S10.** Forest plots of asthma symptom outcome (A-C) symptom scale, (D) ACQ-5

**Fig. S11.** Publication bias assessments for all relative treatment comparisons on asthma symptom outcome

**Fig. S12.** Forest plots of FEV_1_ outcome

**Fig. S13.** Subgroup analysis by asthma step on % predicted FEV_1_ outcome

**Fig. S14.** Subgroup analysis by baseline ICS use on FEV_1_ in liter outcome

**Fig. S15.** Publication bias assessments for all relative treatment comparisons on % predicted FEV_1_ outcome

**Fig. S16.** Forest plots of AQLQ outcome

**Fig. S17.** Subgroup analysis by age group on AQLQ outcome

**Fig. S18.** Subgroup analysis by baseline % predicted FEV_1_ on AQLQ outcome

**Fig. S19.** Publication bias assessments for all relative treatment comparisons on AQLQ

**Fig. S20.** Forest plots of severe adverse events outcome

**Table S1.** Search terms and search strategies

1. Medline via PubMed

| **Domain(s)** | **Search number** | **Query** | |
| --- | --- | --- | --- |
| **Patients** |  |  | |
| P1 (Disease) | #1 | "Asthma"[Mesh] | |
|  | #2 | "wheez*" | |
|  | #3 | "bronchial spasm" | |
|  | #4 | "bronchial constriction" | |
|  | #5 | "bronchial hyperreactivity" | |
|  | #6 | "bronchoconstric*" | |
|  | #7 | "respiratory hypersensitivity" | |
|  | #8 | #1 OR #2 OR #3 OR #4OR #5 OR #6 OR #7 | |
| P2 (Severity) | #9 | "mild" | |
|  | #10 | "intermittent" | |
|  | #11 | #9 OR #10 | |
| P in summary | #12 | #8 AND #11 | |
| **Interventions & comparators** |  |  | |
| ICS/LABA | #13 | "Budesonide, Formoterol Fumarate Drug Combination"[Mesh] | |
|  | #14 | "Symbicort" | |
|  | #15 | "Flutiform" | |
|  | #16 | "Foster" | |
|  | #17 | "Zenhale" | |
|  | #18 | "Relvar" | |
|  | #19 | fluticasone furoate-vilanterol trifenatate | |
|  | #20 | "Mometasone Furoate, Formoterol Fumarate Drug Combination"[Mesh] | |
|  | #21 | #13 OR #14 OR #15 OR #16 OR #17 OR#18 OR #19 OR #20 | |
| ICS | #22 | "Glucocorticoids"[Mesh] | |
|  | #23 | "budesonide*" | |
| **Domain(s)** | **Search number** | **Query** | |
|  | #24 | "beclometasone*" | |
|  | #25 | "fluticasone*" | |
|  | #26 | "ciclesonide*" | |
|  | #27 | "betamethasone*" | |
|  | #28 | "mometasone*" | |
|  | #29 | #22 OR #23 OR #24 OR #25 OR #26 OR#27 OR #28 | |
|  | #30 | "inhal*" | |
|  | #31 | #30 AND #31 | |
| SABA or FABA | #32 | "Albuterol"[Mesh] | |
|  | #33 | "bitolterol" | |
|  | #34 | "carbuterol" | |
|  | #35 | "fenoterol" |  |
|  | #36 | "isoetharine" |  |
|  | #37 | "bronkometer" |  |
|  | #38 | "pirbuterol" |  |
|  | #39 | "maxair" |  |
| **Domain(s)** | **Search number** | **Query** |  |
|  | #40 | "reproterol" |  |
|  | #41 | "rimiterol" |  |
|  | #42 | "salbutamol" |  |
|  | #43 | "Ventolin" |  |
|  | #44 | "levosalbutamol" |  |
|  | #45 | "terbutaline" |  |
|  | #46 | "brethine" |  |
|  | #47 | "bricanyl" |  |
|  | #48 | "brethaire" |  |
|  | #49 | "tulobuterol" |  |
|  | #50 | "metaproterenol" |  |
|  | #51 | "saba" |  |
|  | #52 | "Formoterol Fumarate"[Mesh] |  |
|  | #53 | "eformoterol" |  |
|  | #54 | "atimos" |  |
|  | #55 | "foradil" |  |
|  | #56 | "oxis" |  |
|  | #57 | #32 OR #33 OR #34 OR #35 OR #36 OR #37 OR #38 OR #39 OR #40 OR #41 OR #42 OR #43 OR #44 OR #45 OR #46 OR #47 OR #48 OR #49 OR #50 OR #51 OR #52 OR #53 OR #54 OR #55 OR #56 |  |
| LTRA | #58 | "Leukotriene Antagonists"[Mesh] |  |
|  | #59 | "montelukast" |  |
|  | #60 | "Singulair" |  |
|  | #61 | "zafirlukast" |  |
|  | #62 | "Accolate" |  |
|  | #63 | "pranlukast" |  |
|  | #64 | "antileukotriene*" |  |
|  | #65 | #58 OR #59 OR #60 OR #61 OR #62 OR #63 OR #64 |  |
| I and C in summary | #66 | #21 OR #31 OR #57 OR #65 |  |
| **RCTs** | #67 | "randomized controlled trial" |  |
|  | #68 | "randomized trial" |  |
| **Domain(s)** | **Search number** | **Query** |  |
|  | #69 | "clinical trial" |  |
|  | #70 | "controlled trial" |  |
|  | #71 | "randomized clinical study" |  |
|  | #72 | "randomised controlled trial" |  |
|  | #73 | "randomised trial" |  |
|  | #74 | "randomised clinical study" |  |
| S in summary | #75 | #67 OR #68 OR #69 OR #70 OR #71 OR #72 OR #73 OR #74 |  |
| **Outcomes** | #76 | "exacerbation" |  |
|  | #77 | "asthma attack" |  |
|  | #78 | "asthmatic attack" |  |
|  | #79 | "hospitalization" |  |
|  | #80 | "admission" |  |
|  | #81 | "emergency visit" |  |
| **Domain(s)** | **Search number** | **Query** |  |
|  | #82 | "urgent visit" |  |
|  | #83 | "unscheduled visit" |  |
|  | #84 | "unplanned visit" |  |
|  | #85 | "asthma control" |  |
|  | #86 | "asthma symptom" |  |
|  | #87 | "quality of life" |  |
|  | #88 | "rescue medication" |  |
|  | #89 | "rescue inhaler" |  |
|  | #90 | "systemic steroid*" |  |
|  | #91 | "peak expiratory flow*" |  |
|  | #92 | "exhaled nitric oxide" |  |
|  | #93 | "eosinophil*" |  |
|  | #94 | "adverse events" |  |
|  | #95 | "adverse effects" |  |
| O in summary | #96 | #76 OR #77 OR #78 OR #79 OR #80 OR #81 OR #82 OR #83 OR #84 OR #85 OR #86 OR #87 OR #88 OR #89 OR #90 OR #91 OR #91 OR #93 OR #94 OR #95 |  |
| P, I/C, S, and O | #97 | #12 AND #66 AND #75 AND #96 |  |

**b) Scopus**

| **Domain(s)** | **Search number** | **Query** | |
| --- | --- | --- | --- |
| **Patients** | #1 | TITLE-ABS-KEY (asthma) | |
| P1 Disease | #2 | TITLE-ABS-KEY (wheezing) | |
|  | #3 | TITLE-ABS-KEY (bronchospasm) | |
|  | #4 | TITLE-ABS-KEY (bronchoconstriction) | |
|  | #5 | TITLE-ABS-KEY ("bronchial hyperreactivity") | |
|  | #6 | TITLE-ABS-KEY ("respiratory hypersensitivity") | |
|  | #7 | #1 OR #2 OR #3 OR #4OR #5 OR #6 | |
| P2 (Severity) | #8 | TITLE-ABS-KEY (mild) | |
|  | #9 | TITLE-ABS-KEY (intermittent) | |
|  | #10 | #8 OR #9 | |
| P in summary | #11 | #7 AND #10 | |
| **Domain(s)** | **Search number** | **Query** | |
| **Interventions & comparators** |  |  | |
| ICS/LABA | #10 | TITLE-ABS-KEY (Symbicort) | |
|  | #11 | TITLE-ABS-KEY (flutiform) | |
|  | #12 | TITLE-ABS-KEY (foster) | |
|  | #13 | TITLE-ABS-KEY (zenhale) | |
|  | #14 | TITLE-ABS-KEY ("fluticasone plus vilanterol") | |
|  | #15 | TITLE-ABS-KEY (relvar) | |
|  | #16 | TITLE-ABS-KEY ("budesonide and formoterol") | |
|  | #17 | TITLE-ABS-KEY ("mometasone plus formoterol") | |
|  | #18 | #10 OR #11 OR #12 OR #13 OR #14 OR #15 OR #16 OR #17 | |
| ICS | #19 | TITLE-ABS-KEY (glucocorticoids) | |
|  | #20 | TITLE-ABS-KEY (budesonide) | |
| **Domain(s)** | **Search number** | **Query** | |
|  | #21 | TITLE-ABS-KEY (beclometasone) | |
|  | #22 | TITLE-ABS-KEY (fluticasone) | |
|  | #23 | TITLE-ABS-KEY (ciclesonide) | |
|  | #24 | TITLE-ABS-KEY (betamethasone) | |
|  | #25 | TITLE-ABS-KEY (mometasone) | |
|  | #26 | #19 OR #20 OR #21 OR #22 OR #23 OR #24 OR #25 | |
|  | #27 | TITLE-ABS-KEY (inhaled) | |
|  | #28 | #26 AND #27 | |
| SABA or FABA | #29 | TITLE-ABS-KEY (albuterol) | |
|  | #30 | TITLE-ABS-KEY (bitolterol) | |
|  | #31 | TITLE-ABS-KEY (carbuterol) | |
|  | #32 | TITLE-ABS-KEY (fenoterol) | |
|  | #33 | TITLE-ABS-KEY (bronkometer) |  |
|  | #34 | TITLE-ABS-KEY (pirbuterol) |  |
|  | #35 | TITLE-ABS-KEY (maxair) |  |
|  | #36 | TITLE-ABS-KEY (reproterol) |  |
|  | #37 | TITLE-ABS-KEY (rimiterol) |  |
|  | #38 | TITLE-ABS-KEY (salbutamol) |  |
|  | #39 | TITLE-ABS-KEY (Ventolin) |  |
|  | #40 | TITLE-ABS-KEY (levosalbutamol) |  |
|  | #41 | TITLE-ABS-KEY (terbutaline) |  |
|  | #42 | TITLE-ABS-KEY (brethine) |  |
|  | #43 | TITLE-ABS-KEY (bricanyl) |  |
|  | #44 | TITLE-ABS-KEY (brethaire) |  |
|  | #45 | TITLE-ABS-KEY (tulobuterol) |  |
|  | #46 | TITLE-ABS-KEY (metaproterenol) |  |
|  | #47 | TITLE-ABS-KEY (saba) |  |
|  | #48 | TITLE-ABS-KEY ("formoterol”) |  |
|  | #49 | TITLE-ABS-KEY (eformoterol) |  |
|  | #50 | TITLE-ABS-KEY (atimos) |  |
|  | #51 | TITLE-ABS-KEY (foradil) |  |
|  | #52 | TITLE-ABS-KEY (oxis) |  |
|  | #53 | #29 OR #30 OR #31 OR #32 OR #33 OR #34 OR #35 OR #36 OR #37 OR #38 OR #39 OR #40 OR #41 OR #42 OR #43 OR #44 OR #45 OR #46 OR #47 OR #48 OR #49 OR #50 OR #51 OR #52 |  |
| LTRA | #54 | TITLE-ABS-KEY (“leukotriene antagonists”) |  |
|  | #55 | TITLE-ABS-KEY (montelukast) |  |
| **Domain(s)** | **Search number** | **Query** |  |
|  | #56 | TITLE-ABS-KEY (Singulair) |  |
|  | #57 | TITLE-ABS-KEY (zafirlukast) |  |
|  | #58 | TITLE-ABS-KEY (Accolate) |  |
|  | #59 | TITLE-ABS-KEY (pranlukast) |  |
|  | #60 | #54 OR #55 OR #56 OR #57 OR #58 OR #59 |  |
| I/C in summary | #61 | #18 OR #28 OR #53 OR #60 |  |
| **RCTs** | #62 | TITLE-ABS-KEY (“randomized controlled trial”) |  |
|  | #63 | TITLE-ABS-KEY (“randomized trial”) |  |
|  | #64 | TITLE-ABS-KEY (clinical trial”) |  |
|  | #65 | TITLE-ABS-KEY (“controlled trial”) |  |
|  | #66 | TITLE-ABS-KEY (“randomized clinical study”) |  |
| S in summary | #67 | #62 OR #63 OR #64 OR #65 OR #66 |  |
| **Outcomes** | #68 | TITLE-ABS-KEY (exacerbation) |  |
|  | #69 | TITLE-ABS-KEY (“asthma attack”) |  |
|  | #70 | TITLE-ABS-KEY (“asthmatic attack”) |  |
|  | #71 | TITLE-ABS-KEY (“hospitalization”) |  |
|  | #72 | TITLE-ABS-KEY (“admission”) |  |
|  | #73 | TITLE-ABS-KEY (“emergency visit”) |  |
|  | #74 | TITLE-ABS-KEY (“asthma control”) |  |
|  | #75 | TITLE-ABS-KEY (“asthma symptom”) |  |
|  | #76 | TITLE-ABS-KEY (“quality of life”) |  |
|  | #77 | TITLE-ABS-KEY (rescue medication) |  |
|  | #78 | TITLE-ABS-KEY (“peak expiratory flow*”) |  |
|  | #79 | TITLE-ABS-KEY (“adverse events”) |  |
|  | #80 | TITLE-ABS-KEY (“adverse effects”) |  |
| O in summary | #81 | #68 OR #69 OR #70 OR #71 OR #72 OR #73 OR #74 OR #75 OR #76 OR #77 OR #78 OR #79 OR #80 |  |
| P, I/C, S, and O | #82 | #11 AND #61 AND #67 AND #81 |  |

**Table S2.** Definitions of mild asthma in the included studies

| **Author, Year** | **Asthma Step** | **Terms in title** | **Definition** | **Comments** |
| --- | --- | --- | --- | --- |
| **Children** | | | | |
| Sumino K, 2020 | 2 | Mild asthma | Symptom or SABA use no more than 3-4 days a week, nocturnal symptom no more than 1 night a week | Asthma Evaluation Questionnaire score |
| Zhang Zen Hua, 2020 | 2 | Mild persistent asthma | Symptoms or SABA use >2 times a week but not daily or nocturnal symptoms > 2 nights/month but not more than 1/week, no exacerbation in the past 12 months | GINA 2018 |
| Camargos P, 2018 | 1 & 2 | Mild asthma | 1) symptomatic on SABA alone, 2) controlled with low ICS | Author’s definition |
| Shah MB, 2014 | 2 | Mild persistent asthma | ≥ 3 episodes of wheezing/fast breathing/coughing in the past year that lasted more than 1 d and affected sleep | Revised CDC guidelines mentioned in Nelson Textbook of Pediatrics |
| Visitsunthorn N, 2011 | 2 | Mild persistent asthma | Symptom or SABA use > 1 time a week but < 1 time a day or nocturnal symptoms > 2 times a month but not more than once a week and FEV_1_ >80% predicted | GINA 2002 |
| Martinez FD, 2011 | 2 | Mild persistent asthma | Symptom or SABA use >2 days a week or nocturnal symptom > 2 times a month or controlled with low dose ICS | NAEPP 2007 |
| Chen YZ, 2006 | 2 | Mild persistent asthma | No details | GINA 2002 |
| Becker A, 2006 | 2 | Mild persistent asthma | Symptom or SABA use > 1 time a week but < 1 time a day or nocturnal symptom > 2 times a month but not more than 1/week and FEV_1_ >80% predicted | GINA 2002 |
| Garcia ML, 2005 | 2 | Mild persistent asthma | Symptoms several times a week, no more than 1 attack per day waking at night ≥ 2 times a month | GINA 1998 |
| Arets HGM, 2002 | 1 & 2 | Mild asthma | No details | Author’s definition |
| Waalkens HJ, 1991 | 1 & 2 | Mild asthma | Mild symptoms that were controlled by SABA ‘as needed’ | Author’s definition |
| NCT163293, 2017 | 2 | Mild persistent asthma | No details |  |
| NCT442559, 2022 | 2 | Mild persistent asthma | No details | GINA |
| **Adolescents/ Adults** | | | | |
| Pavord ID, 2020 | 2 | Mild asthma | SABA use <2 occasions per day in the past 4 weeks | Author’s definition |
| Hardy J, 2019 | 2 | Mild asthma (70%) | Symptoms or SABA use at least 2 in the past 4 weeks or nocturnal symptoms at least 1 in the past 4 weeks or well-controlled on low-dose ICS  Remark this study included moderate asthma (30%): uncontrolled on low-dose ICS | GINA 2014 |
| Beasley R, 2019 | 2 | Mild asthma | SABA use <2 occasions per day in the past 4 weeks | Author’s definition |
| **Author, Year** | **Asthma Step** | **Terms in title** | **Definition** | **Comments** |
| Lazarus SC, 2019 | 2 | Mild asthma | Symptoms or SABA use >2 days a week but not daily or nocturnal symptoms > 2 nights a month but not more than once a week | NAEPP2007, GINA2008 |
| O'Byrne PM, 2018 | 2 | Mild asthma | Uncontrolled symptoms on SABA ‘as needed’ or controlled with low-dose ICS or LTRA | GINA 2012 |
| Bateman ED, 2018 | 2 | Mild asthma | Uncontrolled symptoms on SABA ‘as needed’ or controlled with low-dose ICS or LTRA | GINA 2012 |
| Postma DS, 2011 | 2 | Mild persistent asthma | Limited use of rescue medication (not every day), no nocturnal symptom in the past 2 weeks | Author’s definition |
| Renzi PM, 2010 | 1 & 2 | Mild asthma | Symptoms or SABA use at least 3 days in the past 2 weeks | Author’s definition |
| Per-Olof Ehrs, 2010 | 1 | Mild asthma | Symptoms 0−30 mm on VAS scale (no or minor symptoms) | Author’s definition |
| Hoshino M, 2009 | 2 | Mild asthma | Symptoms > 1 time a week but < 1 time a day or night-time symptom > 2 times a month but not more than 1/week and FEV_1_ >80% predicted | GINA 2005 |
| Boulet LP, 2009 | 1 | Mild asthma | Symptoms < 2 times in the past 3 months | Author’s definition |
| Tamaoki J, 2008 | 1 | Mild intermittent asthma | Symptoms < 1 time a week, nocturnal symptoms < 2 times a month and FEV_1_ >80% predicted | GINA 2006 |
| Reddel H, 2008 | 2 | Mild asthma | Symptoms or SABA use <2 days/week and FEV_1_ >80% predicted | GINA 2006, NHLBI 2007 |
| Chuchalin A, 2008 | 2 | Mild asthma | Daytime symptoms at least 3-6 days but not daily in the past 2 weeks and PEF >80% | Author’s definition |
| Boonsawat W, 2008 | 2 | Mild asthma | Daytime symptoms at least 3-6 days but not daily in the past 2 weeks and PEF >80% predicted | Author’s definition |
| Stankovic I, 2007 | 1 | Mild intermittent asthma | Symptoms < 1 time a week, nocturnal symptoms < 2 times a month | GINA 2006 |
| Papi A, 2007 | 2 | Mild persistent asthma | Symptoms or SABA use >2 days/week but not daily or nocturnal symptoms not more than 1/week and FEV_1_ >80% predicted | NHLBI EPR2 1997 |
| Horiguchi T, 2007 | 1 & 2 | Mild intermittent (23%) and  Mild persistent asthma (77%) | Symptoms < 1/week, nocturnal symptoms < 2/month  Symptoms ≥ 1/week, daily life and sleep disturbed >1/month, nocturnal symptoms ≥ 2/month | Japanese Asthma Guideline 2003 |
| Haahtela T, 2006 | 1 | Intermittent asthma | Symptoms or SABA use a maximum of 2 times a week in the past 4 weeks and FEV_1_ >80% predicted | GINA 2005 |
| Zeiger RS, 2005 | 2 | Mild persistent asthma | Daytime symptoms and SABA use ≥2 days per week but not daily in the past 2 weeks | GINA 2002 |
| **Author, Year** | **Asthma Step** | **Terms in title** | **Definition** | **Comments** |
| Bousquet J, 2005 | 2 | Mild persistent asthma | Daytime symptoms and SABA use ≥2 days per week but not daily in the past 2 weeks | GINA 1998 |
| Boushey HA, 2005 | 2 | Mild persistent asthma | Daytime symptoms and SABA use ≥2 days per week but not daily, nighttime awakenings related to asthma > 2 days per month but not more than once a week | NHLBI EPR2 1997 |
| Strand AM, 2004 | 2 | Mild persistent asthma | SABA use ≥2 days per week but not daily in the past 2 months | GINA 1998 |
| Pauwels RA, 2003 | 2 | Mild persistent asthma | Symptoms at least once per week, but not daily in the past 3 months | Author’s definition |
| Yamauchi K, 2001 | 1 & 2 | Mild intermittent (23%) and  Mild persistent asthma (77%) | Symptoms of < 1 time a week, nocturnal symptoms < 2 times a month and FEV_1_ >80% predicted  Symptoms > 1 time a week but < 1time/day or nocturnal symptoms < 2 times a month and FEV_1_ >80% predicted | GINA 1995 |
| O'Byrne PM, 2001 | 2 | Mild persistent asthma | SABA use ≥2 times per week in the past 2 weeks and FEV_1_ >80% predicted | Author’s definition |
| Osterman K, 1997 | 2 | Mild asthma | No details |  |
| NCT1316380, 2015 | 2 | Mild persistent asthma | Symptomatic despite low-dose ICS | Author’s definition |
| NCT455923, 2018 | 2 | Mild persistent asthma | Symptoms > 1 time a week but not every day or nocturnal symptoms no more than once a week and FEV_1_ >80% predicted | GINA |

The classification of mild asthma severity was based on the frequency of symptoms under no controller treatment, otherwise, the symptoms were controlled with low-dose ICS. Abbreviations GINA: Global Initiative for Asthma, NAEPP: National Asthma Education Prevention Program Coordinating Committee Expert Panel, NHLBI: The National Heart Lung and Blood Institute

**Table S3.** Risk of bias of the included studies

| **First author** | **Year** | | **Randomisation process** | | | **Deviation from intended intervention** | | **Missing outcome data** | | **Measuremnt of the outcome** | | **Selection of the reported results** | | **Overall** |
| --- | --- | --- | --- | --- | --- | --- | --- | --- | --- | --- | --- | --- | --- | --- |
| Sumino K | 2020 | |  | | |  | |  | |  | |  | |  |
| Hardy J | 2019 | |  | | |  | |  | |  | |  | |  |
| Beasley R | 2019 | |  | | |  | |  | |  | |  | |  |
| Bateman ED | 2018 | |  | | |  | |  | |  | |  | |  |
| O'Byrne PM | 2018 | |  | | |  | |  | |  | |  | |  |
| Camargos P | 2018 | |  | | |  | |  | |  | |  | |  |
| Shah MB | 2014 | |  | | |  | |  | |  | |  | |  |
| Visitsunthorn N | 2011 | |  | | |  | |  | |  | |  | |  |
| Postma DS | 2011 | |  | | |  | |  | |  | |  | |  |
| Renzi PM | 2010 | |  | | |  | |  | |  | |  | |  |
| Per-Olof Ehrs | 2010 | |  | | |  | |  | |  | |  | |  |
| Hoshino M | 2009 | |  | | |  | |  | |  | |  | |  |
| Boulet LP | 2009 | |  | | |  | |  | |  | |  | |  |
| Tamaoki J | 2008 | |  | | |  | |  | |  | |  | |  |
| Reddel H | 2008 | |  | | |  | |  | |  | |  | |  |
| Chuchalin A | 2008 | |  | | |  | |  | |  | |  | |  |
| Boonsawat W | 2008 | |  | | |  | |  | |  | |  | |  |
| Stankovic I | 2007 | |  | | |  | |  | |  | |  | |  |
| Papi A | 2007 | |  | | |  | |  | |  | |  | |  |
| Haahtela T | 2006 | |  | | |  | |  | |  | |  | |  |
| Zeiger RS | 2005 | |  | | |  | |  | |  | |  | |  |
| **First author** | | **Year** | | **Randomisation process** | **Deviation from intended intervention** | | **Missing outcome data** | | **Measuremnt of the outcome** | | **Selection of the reported results** | | **Overall** | |
| Garcia ML | 2005 | |  | | |  | |  | |  | |  | |  |
| Boushey HA | 2005 | |  | | |  | |  | |  | |  | |  |
| Strand AM | 2004 | |  | | |  | |  | |  | |  | |  |
| Pauwels RA | 2003 | |  | | |  | |  | |  | |  | |  |
| Arets HGM | 2002 | |  | | |  | |  | |  | |  | |  |
| Yamauchi K | 2001 | |  | | |  | |  | |  | |  | |  |
| O'Byrne PM | 2001 | |  | | |  | |  | |  | |  | |  |
| Osterman K | 1997 | |  | | |  | |  | |  | |  | |  |
| Waalkens HJ | 1991 | |  | | |  | |  | |  | |  | |  |
| Zhang Zen Hua | 2020 | |  | | |  | |  | |  | |  | |  |
| Chen YZ | 2006 | |  | | |  | |  | |  | |  | |  |
| Pavord ID | 2020 | |  | | |  | |  | |  | |  | |  |
| Horiguchi T | 2007 | |  | | |  | |  | |  | |  | |  |
| Becker A | 2006 | |  | | |  | |  | |  | |  | |  |
| Lazarus SC | 2019 | |  | | |  | |  | |  | |  | |  |
| Martinez FD | 2011 | |  | | |  | |  | |  | |  | |  |
| Bousquet J | 2005 | |  | | |  | |  | |  | |  | |  |
| NCT1316380 | 2015 | |  | | |  | |  | |  | |  | |  |
| NCT455923 | 2018 | |  | | |  | |  | |  | |  | |  |
| NCT4442559 | 2022 | |  | | |  | |  | |  | |  | |  |
| NCT163293 | 2017 | |  | | |  | |  | |  | |  | |  |

**Table S4. Data synthesis: Formulas for data conversion**

If the original studies reported outcomes as median after treatment or median change from baseline, they were converted to mean or mean change from baseline, respectively. By converting the median into the mean and the first and third quartiles into the SD, we used the following formula:

| Reported outcomes | Formula |
| --- | --- |
| Median after treatment/ Median change from baseline |  |
| Mean | (Q1 + median + Q3) / 3 |
| SD | (Q3-Q1)/ 2ɸ^-1^ $(\frac{0.75n-0.125}{n+0.25})$ ~ $\frac{Q3-Q1}{1.35}$ |
|  |  |
|  |  |
|  |  |

ɸ = the cumulative distribution function of the standard normal distribution which used the formula in Excel spreadsheet as SD = (Q3-Q1)/(2*NORM.INV((0.75*n-0.125)/(n+0.25),0,1)). The post-treatment mean was calculated by summing the pre-treatment mean with the mean change given that the original studies provided a pre-treatment mean and a mean change.

**Table S5.** Study characteristics of included RCTs

| **First author** | **Year** | **Country** | **n** | **Interventions** | **Drug name** | | **Dose (mcg/day)** | | **Treat weeks** | | **Asthma step** | | **Prior**  **ICS** | **Baseline**  **% pred FEV_1_** | | **Adherence**  **(%)** | |
| --- | --- | --- | --- | --- | --- | --- | --- | --- | --- | --- | --- | --- | --- | --- | --- | --- | --- |
| **Children** | | | | | | | | | | | | | | | | | |
| **Martinez FD** | 2011 | USA | 288 | R-ICS, AN-ICS  AN-SABA | BDP | | 80 | | 44 | | 2 | | yes | 100.8 | | ND | |
| **Garcia ML** | 2005 | Spain | 1266 | LTRA  R-ICS | MTK  FP | | 5*  200 | | 48 | | 2 | | yes | 87.1 | | ND | |
| **Waalkens HJ** | 1991 | Nether  lands | 27 | R-ICS/SABA  AN-SABA | BUD/T | | 400/1000 | | 8 | | 1+2 | | no | 92.8 | | ND | |
| **Zhang Zen Hua** | 2020 | China | 120 | R-ICS, AN-ICS | BUD | 200 | | 52 | | 2 | | no | | 83.5 | ND | |  |
| **Becker A** | 2006 | Canada | 587 | LTRA  R-ICS  AN-SABA | MTK  BDP | | 5*  400 | | 56 | | 2 | | no | 92.0 | | ND | |
| **NCT163293** | 2017 | Canada | 239 | R-ICS  AN-SABA | CIC | | 200/400 | | 52 | | 2 | | ND | ND | | ND | |
| **Arets HGM** | 2002 | Nether  lands | 68 | R-ICS  AN-SABA | FP | | 250 | | 12 | | 1+2 | | yes | 102.0 | | ND | |
| **NCT4442559** | 2022 | Korea | 53 | LTRA  R-ICS | MTK  No specified | | 10*  No specified | | 12 | | 2 | | ND | ND | | ND | |
| **Sumino K** | 2020 | USA | 206 | R-ICS, AN-ICS | BDP | | 80 | | 52 | | 2 | | yes | 99.2 | | 71 | |
| **Chen YZ** | 2006 | China | 1974 | R-ICS  AN-SABA | BUD | | 200 | | 156 | | 2 | | yes | 85.5 | | ND | |
| **First author** | **Year** | **Country** | **n** | **Interventions** | **Drug name** | | **Dose (mcg/day)** | | **Treat weeks** | | **Asthma step** | | **Prior**  **ICS** | **Baseline**  **% pred FEV_1_** | | **Adherence**  **(%)** | |
| **Children** |  |  |  |  |  | |  | |  | |  | |  |  | |  | |
| **Visitsunthorn N** | 2011 | Thailand | 29 | LTRA  AN-SABA | MTK | | 5* | | 6 | | 2 | | yes | 87.6 ^a^ | | ND | |
| **Camargos P** | 2018 | Brazil | 1548 | R-ICS, AN-ICS | BDP | | 500 | | 16 | | 1+2 | | yes | 89.0 | | ND | |
| **Shah MB** | 2014 | India | 60 | LTRA  R-ICS | MTK  BUD | | 5*  400 | | 12 | | 2 | | no | 68.8^a^ | | ND | |
| **Adolescents/adults** | | | | | | | | | | | | | | | | | |
| **Pavord ID** | 2020 | NZ | 656 | AN-ICS/FABA  R-ICS  AN-SABA | BUD/F  BUD | | 200/6  400 | | 52 | | 2 | | no | 89.8 | | ND | |
| **Yamauchi K** | 2001 | Japan | 30 | LTRA  R-ICS  AN-SABA | PRN  BDP | | 450*  400 | | 4 | | 1+2 | | no | 95.7 | | ND | |
| **Horiguchi T** | 2007 | Japan | 40 | LTRA  R-ICS | PRN  FP | | 450*  200 | | 8 | | 1+2 | | no | 1.38^b^ | | 87.4 | |
| **Haahtela T** | 2006 | Finland | 92 | AN-ICS/FABA  AN-SABA | BUD/F | | 200/6 | | 24 | | 1 | | no | 101.0 | | ND | |
| **Chuchalin A** | 2008 | Russia | 175 | R-ICS/LABA  R-ICS  AN-SABA | FP/S  FP | | 100/50  200 | | 52 | | 2 | | no | 96.6 | | ND | |
|  |  |  |  |  |  | |  | |  | |  | |  |  | |  | |
| **First author** | **Year** | **Country** | **n** | **Interventions** | **Drug name** | | **Dose (mcg/day)** | | **Treat weeks** | | **Asthma step** | | **Prior**  **ICS** | **Baseline**  **% pred FEV_1_** | | **Adherence**  **(%)** | |
| **Zeiger RS** | 2005 | USA | 679 | LTRA  R-ICS | MTK  FP | | 10*  88 | | 36 | | 2 | | no | 94.1 | | 96.5 | |
| **Pauwels RA** | 2003 | Belgium | 375 | R-ICS  AN-SABA | BUD | | 400 | | 156 | | 2 | | yes | 86.4 | | ND | |
| **Papi A** | 2007 | Italy | 455 | R-ICS/SABA  AN-ICS/SABA  R-ICS  AN-SABA | BDP/A  BDP | | 500/200  500 | | 24 | | 2 | | yes | 88.6 | | ND | |
| **Renzi PM** | 2010 | Canada | 1925 | R-ICS/LABA  R-ICS | FP/S  FP | | 200/100  200 | | 24 | | 1+2 | | no | 92.7 | | ND | |
| **Bateman ED** | 2018 | South Afr | 516 | AN-ICS/FABA  R-ICS | BUD/F  BUD | | 200/6  400 | | 52 | | 2 | | no | 84.2 | | 63.6 | |
| **Per-Olof Ehrs** | 2010 | Sweden | 70 | R-ICS  AN-SABA | FP | | 500 | | 12 | | 1 | | no | 89.9 | | ND | |
| **Osterman K** | 1997 | Sweden | 75 | R-ICS  AN-SABA | BUD | | 400 | | 52 | | 1+2 | | no | 90.9 | | ND | |
| **Bousquet J** | 2005 | Europe | 1945 | LTRA  R-ICS | MTK  FP | | 10*  200 | | 12 | | 2 | | no | 89.4 | | ND | |
| **Boushey HA** | 2005 | USA | 225 | LTRA  R-ICS  AN-SABA | ZAF  BUD | | 40*  400 | | 52 | | 2 | | ND | 88.7 | | ND | |
| **NCT1316380** | 2015 | Argentina | 464 | Tiotropium  AN-SABA | TIO-R | | 2.5, 5 | | 12 | | 2 | | yes | ND | | ND | |
| **First author** | **Year** | **Country** | **n** | **Interventions** | **Drug name** | | **Dose (mcg/day)** | | **Treat weeks** | | **Asthma step** | | **Prior**  **ICS** | **Baseline**  **% pred FEV_1_** | | **Adherence**  **(%)** | |
| **Lazarus SC** | 2019 | USA | 221 | Tiotropium  R-ICS | TIO-R  MF | | 5  440 | | 12 | | 2 | | yes | 92.7 | | ND | |
| **Tamaoki J** | 2008 | Japan | 74 | LTRA  R-ICS | PRN  BUD | | 450  200 | | 8 | | 1 | | no | 84.8 | | 83.5 | |
| **Hoshino M** | 2009 | Japan | 27 | R-ICS/LABA  R-ICS | FP/S  FP | | 200/100  200 | | 8 | | 2 | | yes | 87.2 | | ND | |

BDP: beclomethasone diproprionate, BDP/A: beclomethasone diproprionate/albuterol, BUD: budesonide, BUD/F: budesonide/formoterol, BUD-T: budesonide/terbutaline, CIC: ciclesonide, FP: fluticasone proprionate, FP/S: fluticasone proprionate/salmeterol, MF: mometasone furoate, MTK: montelukast, PRN: pranlukast, TIO-R: tiotropium Respimat, ZAF: zafirlukast. * dose in mg/day. ND: no data, ^a^baseline FEV_1_ as FEV_1_/FVC ratio, ^b^baseline FEV_1_ in liter.

**Treatment effects in children**

**A) Non-severe exacerbation**

*Network meta-analysis*

**Table S6.** Multiple treatment comparisons on non-severe exacerbation outcome

| **Reference treatment** | **Risk ratio (95%CIs)** | | | |
| --- | --- | --- | --- | --- |
|  | **AN-SABA** | **AN-ICS** | **regICS** | **LTRA** |
| **AN-SABA** | 1.9 | 0.74 (0.53,1.02) | 0.61 (0.48,0.78)* | 0.76 (0.58,0.99)* |
| **AN-ICS** |  | 54.5 | 0.83 (0.62,1.12) | 1.03 (0.73,1.43) |
| **regICS** |  |  | 96.2 | 1.23 (1.04,1.45) |
| **LTRA** |  |  |  | 47.3 |

Relative treatment effects are presented as risk ratios (RR) and 95% CI. The effects of the column interventions were compared to those of the row interventions. * indicates statistical significance. The values in the shaded area are SUCRA (relative to AN-SABA). Abbreviations: AN: as-needed, reg: regular

*Transitivity table*

**Table S7.** Comparison of characteristics of children (aged 6-11 years) with mild asthma including in the exacerbation outcomes

| Baseline characteristics | Treatment comparison | | | | | |
| --- | --- | --- | --- | --- | --- | --- |
|  | B-A | C-A | C-B | D-A | D-B | D-C |
|  | AN-ICS vs  SABA | regICS vs  SABA | regICS vs  AN-ICS | LTRA vs  SABA | LTRA vs  AN-ICS | LTRA vs  regICS |
| Age, mean |  |  |  |  |  |  |
| Sumino K, 2020 | - | - | 10.2 | - | - | - |
| Camargos P, 2018 | - | - | 10.3 | - | - | - |
| Shah MB, 2014 | - | - | - | - | - | 5.5 |
| Martinez FD, 2011 | - | 10.9 | 10.9 | - | - | - |
| Becker A, 2006 | - | - | - | 7.6 | - | 7.5 |
| Garcia ML, 2005 | ~~-~~ | - | - | - | - | 9 |
| In total | - | 10.9 | 10.4 | 7.6 | - | 7.3 |
| Female sex, % |  |  |  |  |  |  |
| Sumino K, 2020 | - | - | 46.0 | - | - | - |
| Camargos P, 2018 | - | - | 44.1 | - | - | - |
| Shah MB, 2014 | - | - | - | - | - | 30.0 |
| Martinez FD, 2011 | - | 44.0 | 45.0 | - | - | - |
| Becker A, 2006 | - | - | - | 37.0 | - | 36.0 |
| Garcia ML, 2005 | - | - | - | - | - | 38.4 |
| In total | - | 44.0 | 45.0 | 37.0 | - | 34.8 |
| %predicted FEV_1_, mean |  |  |  |  |  |  |
| Sumino K, 2020 | - | - | 99.2 | - | - | - |
| Camargos P, 2018 | - | - | 89.0 | - | - | - |
| Shah MB, 2014 | - | - | - | - | - | NA |
| Martinez FD, 2011 | - | 100.7 | 101.0 | - | - | - |
| Becker A, 2006 | - | - | - | 92.5 | - | 92.1 |
| Garcia ML, 2005 | - | - | - | - | - | 87.3 |
| In total | - | 100.7 | 96.4 | 92.5 | - | 90.0 |
| Baseline low dose ICS, % |  |  |  |  |  |  |
| Sumino K, 2020 | - | - | 85.5 | - | - | - |
| Camargos P, 2018 | - | - | 89.0 | - | - | - |
| Shah MB, 2014 | - | - | - | - | - | NA |
| Martinez FD, 2011 | - | 78.3 | 76.7 | - | - | - |
| Becker A, 2006 | - | - | - | NA | - | NA |
| Garcia ML, 2005 | - | - | - | - | - | - |
| In total | - | 78.3 | 83.7 | NA | - | NA |
| ACT score, mean |  |  |  |  |  |  |
| Sumino K, 2020 | - | - | 21.5 | - | - | - |
| Camargos P, 2018 | - | - | 22 | - | - | - |
| Shah MB, 2014 | - | - | - | - | - | NA |
| Martinez FD, 2011 | - | 23.7 | 23.5 | - | - | - |
| Becker A, 2006 | - | - | - | NA | - | NA |
| Garcia ML, 2005 | - | - | - | - | - | - |
| In total | - | 23.7 | 22.3 | NA | - | NA |
| Previous exacerbation in the past year, % |  |  |  |  |  |  |
| Sumino K, 2020 | - | - | 1.1 | - | - | - |
| Camargos P, 2018 | - | - | 0 | - | - | - |
| Shah MB, 2014 | - | - | - | - | - | 0 |
| Martinez FD, 2011 | - | 0.6 | 0.6 | - | - | - |
| Becker A, 2006 | - | - | - | 2.0 | - | 2.7 |
| Garcia ML, 2005 | - | - | - | - | - | - |
| In total | NA | 0.6 | 0.6 | 2.0 | NA | 1.4 |

**B) % predicted FEV_1_**

*Network meta-analysis*

**Table S8.** Multiple treatment comparisons of % predicted FEV_1_ outcome

| **Reference Treatment** | **Mean difference (95%CIs)** | | | | |
| --- | --- | --- | --- | --- | --- |
|  | **AN-SABA** | **AN-ICS** | **regICS** | **LTRA** | **reg ICS/SABA** |
| **AN-SABA** | 21.7 | 1.91 (-2.23,6.05) | 4.12 (0.74,7.50)* | 0.92 (-5.65,7.49) | 3.10 (-8.32,14.52) |
| **AN-ICS** |  | 49.9 | 2.21 (-1.07,5.49) | -0.99 (-7.51,5.53) | 1.19 (-10.96,13.34) |
| **regICS** |  |  | 83.5 | -3.20 (-8.83,2.43) | -1.02 (12.93,10.89) |
| **LTRA** |  |  |  | 38.2 | 2.18 (-10.99,15.36) |
| **Reg ICS/SABA** |  |  |  |  | 56.7 |

Relative treatment effects are presented as mean difference (MD) and 95% CI. The effects of the column interventions were compared to those of the row interventions. * indicates statistical significance. The values in the shaded area are SUCRA (relative to AN-SABA). Abbreviations: AN: as-needed, reg: regular

**C) Adverse events**

*Network meta-analysis*

**Table S9.** Multiple treatment comparisons of non-severe adverse events outcome

| **Reference treatment** | **Risk ratio (95%CI)** | | | |
| --- | --- | --- | --- | --- |
|  | **AN-SABA** | **LTRA** | **regICS** | **AN-ICS** |
| **AN-SABA** | 40.9 | 1.08 (0.56,2.08) | 0.88 (0.45,1.72) | 0.59 (0.14,2.57) |
| **LTRA** |  | 29.4 | 0.82 (0.39,1.73) | 0.55 (0.12,2.48) |
| **regICS** |  |  | 56.3 | 0.67 (0.18,2.48) |
| **AN-ICS** |  |  |  | 76.1 |

Relative treatment effects are presented as risk ratios (RR) and 95% CI. The effects of the column interventions were compared to those of the row interventions. The values in the shaded area are SUCRA (relative to AN-SABA). Abbreviations: AN: as-needed, reg: regular

**Treatment effects in adolescents/adults**

**A) Non-severe exacerbation**

*Network meta-analysis*

**Table S10.** Multiple treatment comparisons on non-severe exacerbation outcome

| **Reference treatment** | **Risk ratio (95%CIs)** | | | | | |  |
| --- | --- | --- | --- | --- | --- | --- | --- |
|  | **AN-SABA** | **regICS** | **LTRA** | **AN-ICS/FABA** | **reg**  **ICS/LABA** | **Tiotropium** | |
| **AN-SABA** | 3.0 | 0.50 (0.33,0.76)* | 0.64 (0.31,1.35) | 0.46 (0.28,0.75)* | 0.41 (0.24,0.71)* | 0.44 (0.21,0.94)* | |
| **regICS** |  | 54.8 | 1.29 (0.71,2.32) | 0.92 (0.53,1.59) | 0.82 (0.52,1.30) | 0.88 (0.47,1.63) | |
| **LTRA** |  |  | 32.0 | 0.71 (0.30,1.70) | 0.64 (0.32,1.29) | 0.68 (0.30,1.53) | |
| **AN-ICS/FABA** |  |  |  | 65.7 | 0.90 (0.45,1.79) | 0.96 (0.40,2.29) | |
| **regICS/LABA** |  |  |  |  | 77.0 | 1.06 (0.51,2.24) | |
| **Tiotropium** |  |  |  |  |  | 67.4 | |

Relative treatment effects are presented as risk ratios (RR) and 95% CI. The effects of the column interventions were compared to those of the row interventions. * indicates statistical significance. The values in the shaded area are SUCRA (relative to AN-SABA). Abbreviations: AN: as-needed, reg: regular

*Transitivity table*

**Table S11.** Comparison of characteristics of adolescents/adults with mild asthma including in the exacerbation outcomes

| Baseline characteristics | Step | Treatment comparison | | | | | | | |
| --- | --- | --- | --- | --- | --- | --- | --- | --- | --- |
|  |  | B-A | C-A | C-B | D-A | D-B | E-A | E-B | F-B |
|  |  | regICS vs SABA | LTRA vs SABA | LTRA vs regICS | AN-ICS/FABA vs SABA | AN-ICS/FABA vs regICS | regICS/LABA vs SABA | regICS/LABA vs regICS | Tiotropium vs regICS |
| Age, mean |  |  |  |  |  |  |  |  |  |
| Beasley R, 2019 | 2 | 35.3 | - | - | 35.9 | 35.4 | - | - | - |
| Lazarus SC, 2019 | 2 | - | - | - | - | - | - | - | 31.2 |
| Renzi PM, 2010 | 1&2 | - | - | - | - | - | - | 34.5 | - |
| Boonsawat W, 2008 | 2 | 33.7 | - | - | - | - | 34.0 | 34.3 | - |
| Papi A, 2007 | 2 | 39.3 | - | - | 38.6 | 37.3 | 40.3 | 38.9 | - |
| Zeiger RS, 2005 | 2 | ~~-~~ | - | 35.2 | - | - | - | - | - |
| Bousquet J, 2005 | 2 | - | - | 36.2 | - | - | - | - | - |
| Boushey HA, 2005 | 2 | 32.6 | 32.8 | 33.4 | - | - | - | - | - |
| NCT 1316380, 2015 | 2 | - | - | - | - | - | - | - | 42.8 |
| In total |  | 35.2 | 32.8 | 34.9 | 37.3 | 36.4 | 37.1 | 35.9 | 37 |
| Female sex, % |  |  |  |  |  |  |  |  |  |
| Beasley R, 2019 |  | 54.0 | - | - | 53.1 | 56.4 | - | - | - |
| Lazarus SC, 2019 |  | - | - | - | - | - | - | - | 66 |
| Renzi PM, 2010 |  | - | - | - | - | - | - | 64.0 | - |
| Boonsawat W, 2008 |  | 51.0 | - | - | - | - | 50.0 | 55.0 | - |
| Papi A, 2007 |  | 58.0 | - | - | 58.7 | 58.3 | 59.5 | 59.1 | - |
| Zeiger RS, 2005 |  | ~~-~~ | - | 69.5 | - | - | - | - | - |
| Bousquet J, 2005 |  | - | - | 62.9 | - | - | - | - | - |
| Boushey HA, 2005 |  | 61.5 | 59.5 | 64.0 | - | - | - | - | - |
| NCT 1316380, 2015 |  | - | - | - | - | - | - | - | 60.5 |
| In total |  | 56.1 | 59.5 | 65.5 | 55.9 | 57.3 | 54.7 | 59.4 | 63.2 |
| %predicted FEV_1_, mean |  |  |  |  |  |  |  |  |  |
| Beasley R, 2019 |  | 89.7 | - | - | 89.5 | 90.0 | - | - | - |
| Lazarus SC, 2019 |  | - | - | - | - | - | - | - | 92.7 |
| Renzi PM, 2010 |  | - | - | - | - | - | - | 92.7 | - |
| Boonsawat W, 2008 |  | 96.0 | - | - | - | - | 95.2 | 95.2 | - |
| Papi A, 2007 |  | 88.8 | - | - | 88.7 | 88.6 | 88.1 | 88.0 | - |
| Zeiger RS, 2005 |  | ~~-~~ | - | 93.9 | - | - | - | - | - |
| Bousquet J, 2005 |  | - | - | 89.4 | - | - | - | - | - |
| Boushey HA, 2005 |  | 89.2 | 88.0 | 89.4 | - | - | - | - | - |
| NCT 1316380, 2015 |  | - | - | - | - | - | - | - | 78 |
| In total |  | 91.0 | 88.0 | 90.9 | 89.1 | 89.3 | 91.6 | 92.0 | 85.4 |
| Baseline low-dose ICS, % |  |  |  |  |  |  |  |  |  |
| Beasley R, 2019 |  | 0 | - | - | 0 | 0 | - | - | - |
| Lazarus SC, 2019 |  | - | - | - | - | - | - | - | 20.5 |
| Renzi PM, 2010 |  | - | - | - | - | - | - | NA | - |
| Boonsawat W, 2008 |  | NA | - | - | - | - | NA | NA | - |
| Papi A, 2007 |  | 30.8 | - | - | 30.0 | 30.2 | 33.0 | 33.5 | - |
| Zeiger RS, 2005 |  | ~~-~~ | - | 0 | - | - | - | - | - |
| Bousquet J, 2005 |  | - | - | 0 | - | - | - | - | - |
| Boushey HA, 2005 |  | 0 | 0 | 0 | - | - | - | - | - |
| NCT 1316380, 2015 |  | - | - | - | - | - | - | - | 100 |
| In total |  | 10.3 | 0 | 0 | 14.9 | 15.1 | 33.0 | 33.5 | 60.0 |
| Previous exacerbation in the past year, % |  |  |  |  |  |  |  |  |  |
| Beasley R, 2019 |  | 8.3 | - | - | 7.3 | 6.6 | - | - | - |
| Lazarus SC, 2019 |  | - | - | - | - | - | - | - | 18.6 |
| Renzi PM, 2010 |  | - | - | - | - | - | - | 0 |  |
| Boonsawat W, 2008 |  | 0 | - | - | - | - | 0 | 0 | - |
| Papi A, 2007 |  | NA | - | - | NA | NA | NA | NA | - |
| Zeiger RS, 2005 |  | ~~-~~ | - | 0 | - | - | - | - | - |
| Bousquet J, 2005 |  | - | - | 0 | - | - | - | - | - |
| Boushey HA, 2005 |  | 0 | 0 | 0 | - | - | - | - | - |
| NCT 1316380, 2015 |  | - | - | - | - | - | - | - | 0 |
| In total |  | 2.7 | 0 | 0 | 7.3 | 6.6 | 0 | 0 | 9.3 |

**B) Severe exacerbation**

*Network meta-analysis*

**Table S12.** Multiple treatment comparisons of severe exacerbation outcome

| **Reference treatment** | **Risk ratio (95%CIs)** | | | | | | |
| --- | --- | --- | --- | --- | --- | --- | --- |
|  | **AN-SABA** | **AN-ICS** | **regICS** | **LTRA** | **AN-ICS/FABA** | **reg**  **ICS/LABA** | **Tiotropium** |
| **AN-SABA** | 4.0 | ND | 0.58 (0.46,0.73)* | ND | 0.42 (0.30,0.58)* | 0.39 (0.27,0.56)* | 0.51 (0.13,1.91) |
| **RegICS** |  |  | 36.8 |  | 0.73 (0.54,0.97)* | 0.68 (0.48,0.97)* | 0.88 (0.24,3.24) |
| **AN-ICS/FABA** |  |  |  |  | 75.5 | 0.94 (0.61,1.46) | 1.21 (0.32,4.62) |
| **RegICS/LABA** |  |  |  |  |  | 81.8 | 1.29 (0.33,4.98) |
| **Tiotropium** |  |  |  |  |  |  | 52.0 |

Relative treatment effects are presented as risk ratios (RR) and 95% CI. The effects of the column interventions were compared to those of the row interventions. * indicates statistical significance. The values in shaded area are SUCRA (relative to AN-SABA). Abbreviations: ND: no data, AN: as-needed, reg: regular

*Transitivity table*

**Table S13**. Comparison of characteristics of adolescents/adults with mild asthma including in the severe exacerbation outcomes

| Characteristics | Step | Comparison | | | | | | |
| --- | --- | --- | --- | --- | --- | --- | --- | --- |
|  |  | B-A | D-A | D-B | E-A | E-B | ED | F-B |
|  |  | regICS vs SABA | AN-ICS/FABA vs SABA | AN-ICS/FABA vs regICS | regICS/LABA vs SABA | regICS/LABA vs regICS | regICS/LABA vs AN-ICS/FABA | Tiotropium vs regICS |
| Age, mean |  |  |  |  |  |  |  |  |
| Pavord ID, 2020 | 2 | 35.3 | 35.8 | 35.4 | - | - | - | - |
| Beasley R, 2019 | 2 | 35.3 | 35.8 | 35.4 | - | - | - | - |
| O'Byrne PM, 2018 | 2 | 39.5 | 39.9 | 39.4 | - | - | - | - |
| Postma DS, 2011 | 2 | 31.5 | - | - | 31.0 | 30.5 | - | - |
| Reddel H, 2008 | 2 | 39.2 | - | - | - | - | - | - |
| Papi A, 2007 | 2 | 39.3 | 38.7 | 37.3 | 40.3 | 38.9 | 38.3 | - |
| Pauwels RA, 2003 | 2 | NA | - | - | - | - | - | - |
| O'Byrne PM, 2001 | 2 | 33.7 | 33.2 | 34.6 | - | - | - | - |
| Hardy J, 2019 | 2 | - | - | 43.1 | - | - | - | - |
| Bateman ED, 2018 | 2 | - | - | 41.0 | - | - | - | - |
| Renzi PM, 2010 | 2 | - | - | - | - | 34.6 | - | - |
| Chuchalin A, 2008 | 2 | 34.1 | - | - | 34.1 | 33.8 | - | - |
| Strand AM, 2004 | 2 | - | - | - | - | 38.5 | - | - |
| NCT 455923 | 2 | ~~-~~ | ~~-~~ | ~~-~~ | ~~-~~ | NA | ~~-~~ | ~~-~~ |
| NCT 1316380 | 2 | - | - | - | - | - | - | 42.8 |
| In total |  | 36.0 | 36.7 | 38.0 | 35.1 | 35.2 | 38.3 | 42.8 |
| Female sex, % |  |  |  |  |  |  |  |  |
| Pavord ID, 2020 |  | 54.0 | 53.1 | 56.4 | - | - | - | - |
| Beasley R, 2019 |  | 54.0 | 53.1 | 56.4 | - | - | - | - |
| O'Byrne PM, 2018 |  | 61.3 | 60.6 | 61.5 | - | - | - | - |
| Postma DS, 2011 |  | 59.0 | - | - | 57.5 | 57.5 | - | - |
| Reddel H, 2008 |  | 63.7 | - | - | - | - | - | - |
| Papi A, 2007 |  | 58.0 | 58.8 | 58.3 | 59.5 | 59.1 | 59.7 | - |
| Pauwels RA, 2003 |  | 54.1 | - | - | - | - | - | - |
| O'Byrne PM, 2001 |  | 57.4 | 58.4 | 58.0 | - | - | - | - |
| Hardy J, 2019 |  | - | - | 54.0 | - | - | - | - |
| Bateman ED, 2018 |  | - | - | 61.8 | - | - | - | - |
| Renzi PM, 2010 |  | - | - | - | - | 64.0 | - | - |
| Chuchalin A, 2008 |  | 58.7 | - | - | 57.2 | 57.0 | - | - |
| Strand AM, 2004 |  | - | - | - | - | 56.8 | - | - |
| NCT 455923 |  | ~~-~~ | ~~-~~ | ~~-~~ | ~~-~~ | 63.0 | - | - |
| NCT 1316380 |  | - | - | - | - | - | - | 60.5 |
| In total |  | 57.8 | 56.8 | 58.3 | 58.1 | 59.5 | 59.7 | 60.5 |
| %predicted FEV_1_, mean |  |  |  |  |  |  |  |  |
| Pavord ID, 2020 |  | 89.7 | 89.2 | 90.0 | - | - | - | - |
| Beasley R, 2019 |  | 89.7 | 89.2 | 90.0 | - | - | - | - |
| O'Byrne PM, 2018 |  | 84.2 | 84.1 | 84.2 | - | - | - | - |
| Postma DS, 2011 |  | 95.5 | - | - | 96.6 | 95.6 | - | - |
| Reddel H, 2008 |  | 87.1 | - | - | - | - | - | - |
| Papi A, 2007 |  | 88.8 | 88.9 | 88.8 | 88.1 | 88.0 | 87.9 | - |
| Pauwels RA, 2003 |  | 86.4 | - | - | - | - | - | - |
| O'Byrne PM, 2001 |  | 88.5 | 89.9 | 87.9 | - | - | - | - |
| Hardy J, 2019 |  | - | - | 87.6 | - | - | - | - |
| Bateman ED, 2018 |  | - | - | 84.2 | - | - | - | - |
| Renzi PM, 2010 |  | - | - | - | - | 92.7 | - | - |
| Chuchalin A, 2008 |  | 96.5 | - | - | 97.0 | 96.4 | - | - |
| Strand AM, 2004 |  | - | - | - | - | 80 | - | - |
| NCT 455923 |  | ~~-~~ | ~~-~~ | ~~-~~ | ~~-~~ | NA | - | - |
| NCT 1316380 |  | - | - | - | - | - | - | 78 |
| In total |  | 89.9 | 88.0 | 87.5 | 93.9 | 90.5 | 87.9 | 78 |
| Baseline low-dose ICS, % |  |  |  |  |  |  |  |  |
| Pavord ID, 2020 |  | 0 | 0 | 0 | - | - | - | - |
| Beasley R, 2019 |  | 0 | 0 | 0 | - | - | - | - |
| O'Byrne PM, 2018 |  | 55.4 | 55.4 | 55.8 | - | - | - | - |
| Postma DS, 2011 |  | NA | - | - | NA | NA | - | ~~-~~ |
| Reddel H, 2008 |  | 33.6 | - | - | - | - | - | - |
| Papi A, 2007 |  | 30.8 | 30.0 | 30.2 | 33.0 | 33.5 | 32.5 | - |
| Pauwels RA, 2003 |  | 0 | - | - | - | - | - | - |
| O'Byrne PM, 2001 |  | 64.5 | 64.5 | 64.5 | - | - | - | - |
| Hardy J, 2019 |  | - | - | 70.5 | - | - | - | - |
| Bateman ED, 2018 |  | - | - | 53.7 | - | - | - | - |
| Renzi PM, 2010 |  | - | - | - | - | NA | - | - |
| Chuchalin A, 2008 |  | ND | - | - | NA | NA | - | - |
| Strand AM, 2004 |  | - | - | - | - | 0 | - | - |
| NCT 455923 |  | ~~-~~ | ~~-~~ | ~~-~~ | ~~-~~ | NA | - | - |
| NCT 1316380 |  | - | - | - | - | - | - | 100 |
| In total |  | 26.3 | 29.9 | 39.2 | 33.0 | 16.7 | 32.5 | 100 |
| Previous exacerbation in the past year, % |  |  |  |  |  |  |  |  |
| Pavord ID, 2020 |  | 8.3 | 7.3 | 6.6 | - | - | - | - |
| Beasley R, 2019 |  | 8.3 | 7.3 | 6.6 | - | - | - | - |
| O'Byrne PM, 2018 |  | 19.4 | 20.0 | 19.4 | - | - | - | - |
| Postma DS, 2011 |  | 0 | - | - | 0 | 0 | - | ~~-~~ |
| Reddel H, 2008 |  | 0 | - | - | - | - | - | - |
| Papi A, 2007 |  | 0 | 0 | 0 | 0 | 0 | 0 | - |
| Pauwels RA, 2003 |  | 0 | - | - | - | - | - | - |
| O'Byrne PM, 2001 |  | NA | NA | NA | - | - | - | - |
| Hardy J, 2019 |  | - | - | 12.0 | - | - | - | - |
| Bateman ED, 2018 |  | - | - | 22.5 | - | - | - | - |
| Renzi PM, 2010 |  | - | - | - | - | 0 | - | - |
| Chuchalin A, 2008 |  | NA | - | - | NA | NA | - | - |
| Strand AM, 2004 |  | - | - | - | - | 0 | - | - |
| NCT 455923 |  | ~~-~~ | ~~-~~ | ~~-~~ | ~~-~~ | NA | - | - |
| NCT 1316380 |  | - | - | - | - | - | - | 0 |
| In total |  | 5.1 | 8.6 | 11.1 | 0 | 0 | 0 | 0 |

**C) Asthma symptoms**

*Network meta-analysis*

**Table S14.** Multiple treatment comparisons of asthma symptom scale outcome

| **Reference treatment** | **SMD (95%CI)** | | | | |
| --- | --- | --- | --- | --- | --- |
|  | **AN-SABA** | **R-ICS** | **LTRA** | **AN-ICS/FABA** | **R-ICS/LABA** |
| **AN-SABA** | 12.0 | -0.33 (-0.90,0.24) | -0.94 (-1.93,0.04) | -0.48 (-1.49,0.54) | -0.41 (-1.17,0.35) |
| **R-ICS** |  | 44.5 | -0.61 (-1.49,0.27) | -0.14 (-1.21,0.93) | -0.08 (-0.81,0.65) |
| **LTRA** |  |  | 85.1 | 0.47 (-0.88,1.82) | 0.53 (-0.58,1.65) |
| **AN-ICS/FABA** |  |  |  | 56.0 | 0.07 (-1.07,1.20) |
| **R-ICS/LABA** |  |  |  |  | 52.5 |

Relative treatment effects are presented as standardized mean difference (SMD) and 95% CI. The effects of the column interventions were compared to those of the row interventions. The values in the shaded column are SUCRA (relative to AN-SABA). Abbreviations: AN: as-needed, R: regular

**Table S15.** Multiple treatment comparisons of asthma symptom scale outcome (sensitivity analysis)

| **Reference treatment** | **SMD (95%CI)** | | | | |
| --- | --- | --- | --- | --- | --- |
|  | **AN-SABA** | **R-ICS** | **LTRA** | **AN-ICS/FABA** | **R-ICS/LABA** |
| **AN-SABA** | 7.0 | -0.40  (-0.59, -0.21)* | -0.10  (-0.45,0.24) | -0.52  (-0.85, -0.19)* | -0.43  (-0.66, -0.20)* |
| **R-ICS** |  | 65.2 | 0.29  (-0.03,0.62) | -0.12  (-0.47,0.23) | -0.04  (-0.26,0.19) |
| **LTRA** |  |  | 21.9 | -0.41  (-0.87,0.05) | -0.33  (-0.71,0.05) |
| **AN-ICS/FABA** |  |  |  | 83.9 | 0.08  (-0.28,0.45) |
| **R-ICS/LABA** |  |  |  |  | 71.9 |

Sensitivity analysis excluded the study comparing LTRA vs regular ICS in which higher baseline eosinophil level was a source of heterogeneity. Relative treatment effects are presented as standardized mean difference (SMD) and 95% CI. The effects of the column interventions were compared to those of the row interventions. * indicates statistical significance. The values in the shaded area are SUCRA (relative to AN-SABA). Abbreviations: AN: as-needed, R: regular

**D) % predicted FEV_1_**

*Network meta-analysis*

**Table S16.** Multiple treatment comparisons of % predicted FEV_1_ outcome

| **Reference Treatment** | **MD (95%CIs)** | | | | |
| --- | --- | --- | --- | --- | --- |
|  | **AN-SABA** | **R-ICS** | **LTRA** | **AN-ICS/FABA** | **R-ICS/LABA** |
| **AN-SABA** | 13.1 | 3.53 (1.68,5.38)* | -0.20 (-3.64,3.25) | 4.45 (1.76,7.15)* | 3.45 (0.40,6.49)* |
| **R-ICS** |  | 69.8 | -3.73 (-6.81,-0.65)* | 0.92 (-1.92,3.76) | -0.08 (-3.06,2.89) |
| **LTRA** |  |  | 14.3 | 4.65 (0.54,8.76)* | 3.65 (-0.62,7.91) |
| **AN-ICS/FABA** |  |  |  | 86.1 | -1.00 (-4.57,2.56) |
| **R-ICS/LABA** |  |  |  |  | 66.7 |

Relative treatment effects are presented as mean difference (MD) and 95% CI. The effects of the column interventions were compared to those of the row interventions. * indicates statistical significance. The values in the shaded area are SUCRA (relative to AN-SABA). Abbreviations: AN: as-needed, R: regular

**E) Asthma-specific quality-of-life**

*Network meta-analysis*

**Table S17***.* Multiple treatment comparisons of AQLQ

| **Reference treatment** | **MD (95%CIs)** | | | |
| --- | --- | --- | --- | --- |
|  | **AN-SABA** | **R-ICS** | **LTRA** | **R-ICS/LABA** |
| **AN-SABA** | 23.6 | 0.21 (0.07,0.35)* | -0.03 (-0.19,0.13) | 0.12 (-0.04,0.28) |
| **R-ICS** |  | 95.6 | -0.24 (-0.36,-0.13)* | -0.09 (-0.25,0.07) |
| **LTRA** |  |  | 14.1 | 0.15 (-0.04,0.35) |
| **R-ICS/LABA** |  |  |  | 66.7 |

Relative treatment effects are presented as mean difference (MD) and 95% CI. The effects of the column interventions were compared to those of the row interventions. * indicates statistical significance. The values in the shaded area are SUCRA (relative to AN-SABA). Abbreviations: AN: as-needed, R: regular

**F) Severe adverse events**

*Network meta-analysis*

**Table S18.** Multiple treatment comparisons of severe adverse event outcome

| **Reference treatment** | **RR (95%CIs)** | | | |
| --- | --- | --- | --- | --- |
|  | **AN-SABA** | **R-ICS** | **AN-ICS/FABA** | **R-ICS/LABA** |
| **AN-SABA** | 6.1 | 0.65 (0.45,0.94)* | 0.79 (0.52,1.21) | 0.57 (0.31,1.02) |
| **R-ICS** |  | 71.6 | 0.83 (0.58,1.18) | 0.87 (0.49,1.54) |
| **AN-ICS/FABA** |  |  | 38.7 | 0.71 (0.37,1.39) |
| **R-ICS/LABA** |  |  |  | 83.6 |

Relative treatment effects are presented as risk ratios (RR) and 95% CI. The effects of the column interventions were compared to those of the row interventions. * indicates statistical significance. The values in the shaded area are SUCRA (relative to AN-SABA). Abbreviations: AN: as-needed, R: regular

**Grading the evidence of the network meta-analysis using CINeMA**

We evaluated the certainty of evidence for all the outcomes by using the Confidence in Network Meta-Analysis Software CINeMA, which covers 6 domains: (1) within-study bias, (2) reporting bias, (3) indirectness, (4) imprecision, (5) heterogeneity, and (6) incoherence and was considered to be a transparent, rigorous, and comprehensive system.

Based on the available evidence, for comparisons of two treatment interventions the clinically meaningful threshold was set at a risk ratio of 0.8 and 1.25 for dichotomous outcomes (i.e., exacerbation, adverse events) and at mean differences of -0.5 and 0.5 for continuous outcome (i.e., symptom score and AQLQ) and mean differences of -10 and 10 for % predicted FEV_1_.

**Table S19.** Rating of confidence in NMA in children

A) Rating of confidence in NMA on exacerbation

| **Comparison** | **N of**  **studies** | **Within-study**  **bias** | **Reporting**  **bias** | **Indirect**  **ness** | **Imprecision** | **Heteroge**  **neity** | **Incoherence** | **Confidence**  **rating** |
| --- | --- | --- | --- | --- | --- | --- | --- | --- |
| an-ics:an-saba | 1 | No concerns | Low risk | No concerns | Some concerns | No concerns | No concerns | Moderate |
| an-ics:r-ics | 3 | No concerns | Low risk | No concerns | Some concerns | Some concerns | No concerns | Low |
| an-saba:ltra | 1 | Some concerns | Low risk | No concerns | No concerns | Some concerns | No concerns | Low |
| an-saba:r-ics | 2 | No concerns | Low risk | No concerns | No concerns | No concerns | No concerns | High |
| ltra:r-ics | 3 | Some concerns | Low risk | No concerns | No concerns | Some concerns | No concerns | Low |
| an-ics:ltra | 0 | Some concerns | Low risk | No concerns | Major concerns | No concerns | No concerns | Very low |

B) Rating of confidence in NMA on % predicted FEV_1_

| **Comparison** | **N of**  **studies** | **Within-study**  **bias** | **Reporting**  **bias** | **Indirect**  **ness** | **Imprecision** | **Heteroge**  **neity** | **Incoherence** | **Confidence**  **rating** |
| --- | --- | --- | --- | --- | --- | --- | --- | --- |
| an-ics:an-saba | 1 | No concerns | Low risk | No concerns | Some concerns | Some concerns | No concerns | Low |
| an-ics:r-ics | 3 | No concerns | Low risk | No concerns | Some concerns | Some concerns | No concerns | Low |
| an-saba:r-ics | 3 | Some concerns | Low risk | No concerns | No concerns | Major concerns | No concerns | Very low |
| ltra:r-ics | 1 | Some concerns | Low risk | No concerns | Some concerns | Some concerns | No concerns | Low |
| an-ics:ltra | 0 | Some concerns | Low risk | No concerns | Major concerns | No concerns | No concerns | Very low |
| an-saba:ltra | 0 | Some concerns | Low risk | No concerns | Major concerns | No concerns | No concerns | Very low |

C) Rating of confidence in NMA on adverse events

| **Comparison** | **N of**  **studies** | **Within-study**  **bias** | **Reporting**  **bias** | **Indirect**  **ness** | **Imprecision** | **Heterogeneity** | **Incoherence** | **Confidence**  **rating** |
| --- | --- | --- | --- | --- | --- | --- | --- | --- |
| an-ics:r-ics | 1 | No concerns | Low risk | No concerns | Major concerns | No concerns | No concerns | Low |
| an-saba:ltra | 2 | Some concerns | Low risk | No concerns | Major concerns | No concerns | No concerns | Very low |
| an-saba:r-ics | 1 | Some concerns | Low risk | No concerns | Major concerns | No concerns | No concerns | Very low |
| ltra:r-ics | 3 | Some concerns | Low risk | No concerns | Major concerns | No concerns | No concerns | Very low |
| an-ics:an-saba | 0 | Some concerns | Low risk | No concerns | Major concerns | No concerns | No concerns | Very low |
| an-ics:ltra | 0 | Some concerns | Low risk | No concerns | Major concerns | No concerns | No concerns | Very low |

**Table S20.** Rating of confidence in NMA in adolescents/adults

A) Rating of confidence in NMA on exacerbation

| **Comparison** | **N of**  **studies** | **Within-study**  **bias** | **Reporting**  **bias** | **Indirect**  **ness** | **Imprecision** | **Heterogeneity** | **Incoherence** | **Confidence**  **rating** |
| --- | --- | --- | --- | --- | --- | --- | --- | --- |
| an-ics/faba:  an-saba | 2 | Some concerns | Low risk | No concerns | No concerns | No concerns | No concerns | Moderate |
| an-ics/faba:r-ics | 2 | Some concerns | Low risk | No concerns | Major concerns | No concerns | Some concerns | Very low |
| an-ics/faba:  r-ics/laba | 1 | Some concerns | Low risk | No concerns | Major concerns | No concerns | Some concerns | Very low |
| an-saba:ltra | 1 | Some concerns | Low risk | No concerns | Some concerns | Some concerns | No concerns | Low |
| an-saba:r-ics | 4 | Some concerns | Low risk | No concerns | No concerns | No concerns | No concerns | Moderate |
| an-saba:  r-ics/laba | 2 | Some concerns | Low risk | No concerns | No concerns | No concerns | No concerns | Moderate |
| ltra:r-ics | 3 | Some concerns | Low risk | No concerns | Major concerns | No concerns | No concerns | Very low |
| r-ics:r-ics/laba | 3 | Some concerns | Low risk | No concerns | Some concerns | Some concerns | No concerns | Low |
| r-ics:tio | 2 | Some concerns | Low risk | No concerns | Major concerns | No concerns | No concerns | Very low |
| an-ics/faba:ltra | 0 | Some concerns | Low risk | No concerns | Major concerns | No concerns | No concerns | Very low |
| an-ics/faba:tio | 0 | Some concerns | Low risk | No concerns | Major concerns | No concerns | No concerns | Very low |
| an-saba:tio | 0 | Some concerns | Low risk | No concerns | No concerns | Some concerns | No concerns | Low |
| ltra:r-ics/laba | 0 | Some concerns | Low risk | No concerns | Some concerns | Some concerns | No concerns | Low |
| ltra:tio | 0 | Some concerns | Low risk | No concerns | Major concerns | No concerns | No concerns | Very low |
| r-ics/laba:tio | 0 | Some concerns | Low risk | No concerns | Major concerns | No concerns | No concerns | Very low |

B) Rating of confidence in NMA on severe exacerbation

| **Comparison** | **N of**  **studies** | **Within-study**  **bias** | **Reporting**  **bias** | **Indirect**  **ness** | **Imprecision** | **Heterogeneity** | **Incoherence** | **Confidence**  **rating** |
| --- | --- | --- | --- | --- | --- | --- | --- | --- |
| an-ics/faba:  an-saba | 4 | No concerns | Low risk | No concerns | No concerns | No concerns | No concerns | High |
| an-ics/faba:r-ics | 5 | No concerns | Low risk | No concerns | No concerns | Some concerns | No concerns | Moderate |
| an-ics/faba:  r-ics/laba | 1 | Some concerns | Low risk | No concerns | Major concerns | No concerns | No concerns | Very low |
| an-saba:r-ics | 9 | Some concerns | Low risk | No concerns | No concerns | Some concerns | No concerns | Low |
| an-saba:  r-ics/laba | 4 | Some concerns | Low risk | No concerns | No concerns | No concerns | No concerns | Moderate |
| ltra:r-ics | 1 | No concerns | Low risk | No concerns | Major concerns | No concerns | No concerns | Low |
| r-ics:r-ics/laba | 7 | Some concerns | Low risk | No concerns | No concerns | Some concerns | No concerns | Low |
| r-ics:r-ics/laba | 1 | Some concerns | Low risk | No concerns | Major concerns | No concerns | No concerns | Very low |
| r-ics:tio | 1 | Some concerns | Low risk | No concerns | Major concerns | No concerns | No concerns | Very low |
| an-ics/  faba:ltra | 0 | No concerns | Low risk | No concerns | Major concerns | No concerns | No concerns | Low |
| an-ics/faba:  r-ics/laba | 0 | Some concerns | Low risk | No concerns | Major concerns | No concerns | No concerns | Very low |
| an-ics/faba:tio | 0 | Some concerns | Low risk | No concerns | Major concerns | No concerns | No concerns | Very low |
| an-saba:ltra | 0 | No concerns | Low risk | No concerns | Major concerns | No concerns | No concerns | Low |
| an-saba:  r-ics/laba | 0 | Some concerns | Low risk | No concerns | No concerns | Some concerns | No concerns | Low |
| an-saba:tio | 0 | Some concerns | Low risk | No concerns | Major concerns | No concerns | No concerns | Very low |
| ltra:r-ics/laba | 0 | No concerns | Low risk | No concerns | Major concerns | No concerns | No concerns | Low |
| ltra:tio | 0 | Some concerns | Low risk | No concerns | Major concerns | No concerns | No concerns | Very low |
| r-ics/laba:tio | 0 | Some concerns | Low risk | No concerns | Major concerns | No concerns | No concerns | Very low |

C) Rating of confidence in NMA on asthma symptom score

| **Comparison** | **N of**  **studies** | **Within-study**  **bias** | **Reporting**  **bias** | **Indirect**  **ness** | **Imprecision** | **Heterogeneity** | **Incoherence** | **Confidence**  **rating** |
| --- | --- | --- | --- | --- | --- | --- | --- | --- |
| an-ics/faba:  an-saba | 2 | Some concerns | Low risk | No concerns | Some concerns | Some concerns | No concerns | Low |
| an-ics/faba:r-ics | 1 | Some concerns | Low risk | No concerns | Some concerns | Some concerns | No concerns | Low |
| an-ics/faba:  r-ics/laba | 1 | Some concerns | Low risk | No concerns | Major concerns | No concerns | No concerns | Very low |
| an-saba:ltra | 1 | Some concerns | Low risk | No concerns | No concerns | Some concerns | Some concerns | Low |
| an-saba:r-ics | 7 | Some concerns | Low risk | No concerns | No concerns | Major concerns | Some concerns | Very low |
| an-saba:  r-ics/laba | 3 | Some concerns | Low risk | No concerns | No concerns | Major concerns | No concerns | Very low |
| ltra:r-ics | 3 | Some concerns | Low risk | No concerns | Some concerns | Some concerns | Some concerns | Low |
| r-ics:r-ics/laba | 4 | Some concerns | Low risk | No concerns | No concerns | Major concerns | No concerns | Very low |
| an-ics/faba:ltra | 0 | Some concerns | Low risk | No concerns | Some concerns | Some concerns | No concerns | Low |
| ltra:r-ics/laba | 0 | Some concerns | Low risk | No concerns | Some concerns | Some concerns | No concerns | Low |

D) Rating of confidence in NMA on % predicted FEV_1_

| **Comparison** | **N of**  **studies** | **Within-study**  **bias** | **Reporting**  **bias** | **Indirect**  **ness** | **Imprecision** | **Heterogeneity** | **Incoherence** | **Confidence**  **rating** |
| --- | --- | --- | --- | --- | --- | --- | --- | --- |
| an-ics/faba:an-saba | 3 | Some concerns | Low risk | No concerns | No concerns | No concerns | No concerns | Moderate |
| an-ics/faba:r-ics | 2 | Some concerns | Low risk | No concerns | No concerns | No concerns | No concerns | Moderate |
| an-ics/faba:r-ics/laba | 1 | Some concerns | Low risk | No concerns | No concerns | No concerns | No concerns | Moderate |
| an-saba:ltra | 1 | Some concerns | Low risk | No concerns | No concerns | No concerns | No concerns | Moderate |
| an-saba:r-ics | 9 | Some concerns | Low risk | No concerns | No concerns | No concerns | No concerns | Moderate |
| an-saba:r-ics/laba | 2 | Some concerns | Low risk | No concerns | No concerns | No concerns | Some concerns | Low |
| ltra:r-ics | 3 | Some concerns | Low risk | No concerns | No concerns | No concerns | No concerns | Moderate |
| r-ics:r-ics/laba | 3 | Some concerns | Low risk | No concerns | No concerns | No concerns | Some concerns | Low |
| an-ics/faba:ltra | 0 | Some concerns | Low risk | No concerns | No concerns | Some concerns | No concerns | Low |
| ltra:r-ics/laba | 0 | Some concerns | Low risk | No concerns | No concerns | Some concerns | No concerns | Low |

E) Rating of confidence in NMA on asthma-specific quality-of-life

| **Comparison** | **N of**  **studies** | **Within-study**  **bias** | **Reporting**  **bias** | **Indirect**  **ness** | **Imprecision** | **Heterogeneity** | **Incoherence** | **Confidence**  **rating** |
| --- | --- | --- | --- | --- | --- | --- | --- | --- |
| an-ics/faba:  an-saba | 1 | No concerns | Low risk | No concerns | No concerns | No concerns | No concerns | High |
| an-ics/faba:r-ics | 2 | No concerns | Low risk | No concerns | No concerns | No concerns | No concerns | High |
| an-saba:ltra | 1 | Some concerns | Low risk | No concerns | No concerns | No concerns | No concerns | Moderate |
| an-saba:r-ics | 4 | Some concerns | Low risk | No concerns | No concerns | No concerns | No concerns | Moderate |
| an-saba:  r-ics/laba | 1 | Some concerns | Low risk | No concerns | No concerns | No concerns | No concerns | Moderate |
| ltra:r-ics | 3 | Some concerns | Low risk | No concerns | No concerns | Some concerns | No concerns | Low |
| r-ics:r-ics/laba | 1 | Some concerns | Low risk | No concerns | No concerns | No concerns | No concerns | Moderate |
| an-ics/faba:ltra | 0 | No concerns | Low risk | No concerns | No concerns | Some concerns | Major concerns | Very low |
| an-ics/faba:  r-ics/laba | 0 | Some concerns | Low risk | No concerns | No concerns | No concerns | Major concerns | Very low |
| ltra:r-ics/laba | 0 | Some concerns | Low risk | No concerns | No concerns | Some concerns | Major concerns | Very low |

F) Rating of confidence in NMA on severe adverse events

| **Comparison** | **N of**  **studies** | **Within-study**  **bias** | **Reporting**  **bias** | **Indirect**  **ness** | **Imprecision** | **Heterogeneity** | **Incoherence** | **Confidence**  **rating** |
| --- | --- | --- | --- | --- | --- | --- | --- | --- |
| an-ics/faba:  an-saba | 3 | No concerns | Low risk | No concerns | Some concerns | No concerns | No concerns | Moderate |
| an-ics/faba:r-ics | 5 | No concerns | Low risk | No concerns | Some concerns | No concerns | No concerns | Moderate |
| an-ics/faba:  r-ics/laba | 1 | Some concerns | Low risk | No concerns | Major concerns | No concerns | No concerns | Very low |
| an-saba:r-ics | 6 | No concerns | Low risk | No concerns | No concerns | No concerns | No concerns | High |
| an-saba:  r-ics/laba | 2 | Some concerns | Low risk | No concerns | No concerns | Some concerns | No concerns | Low |
| r-ics:r-ics/laba | 2 | Some concerns | Low risk | No concerns | Major concerns | No concerns | No concerns | Very low |

**Fig. S1.** Risk of bias assessment according to the Revised Cochrane risk-of-bias tool for randomized trials (RoB2) algorithm


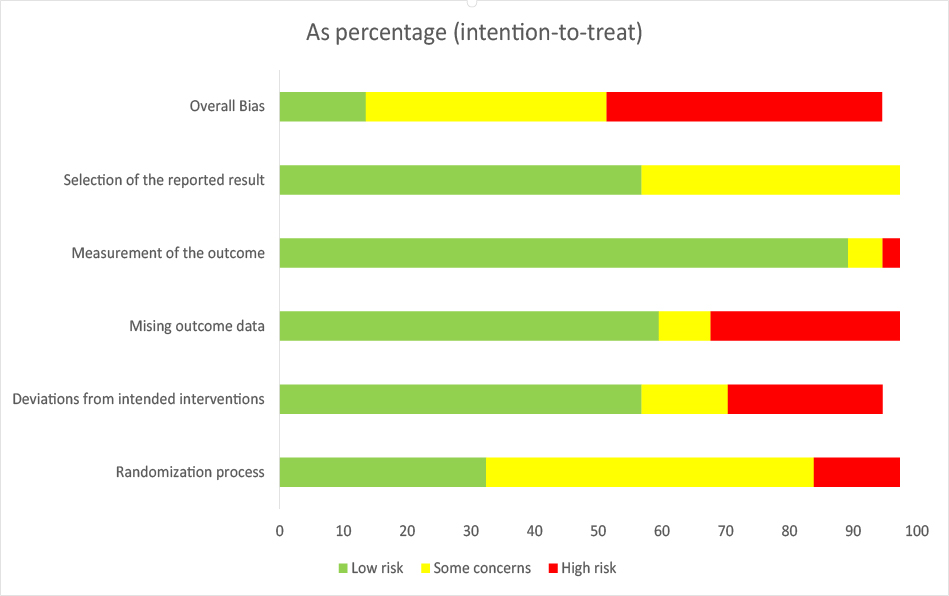


**Treatment effects in children**

**A) Non-severe exacerbation**

*Direct meta-analysis*

**Fig. S2**. Forest plots of non-severe exacerbation outcome


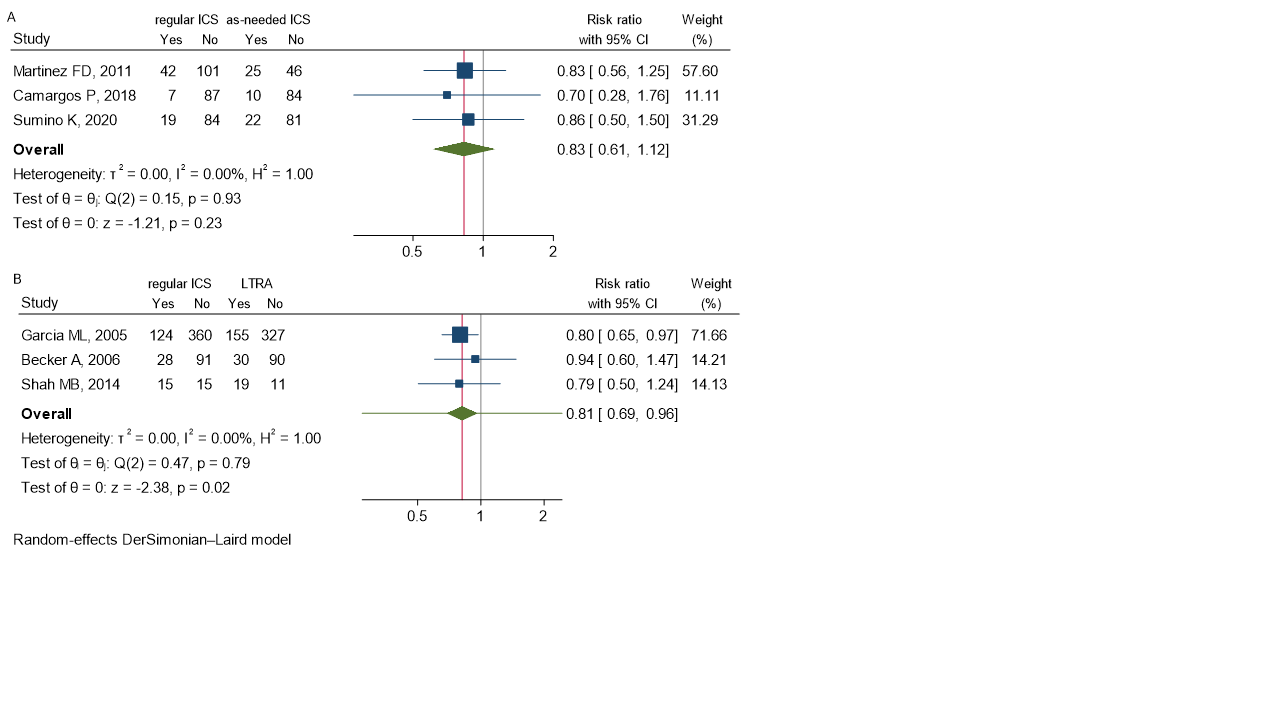


A) regular ICS vs. AN-ICS, and B) regular ICS vs. LTRA. No pooling for the comparison between regular ICS and AN-SABA (2 RCTs).

**B) % predicted FEV_1_**

*Direct meta-analysis*

**Fig. S3.** Forest plots of %predicted FEV_1_ outcome

**
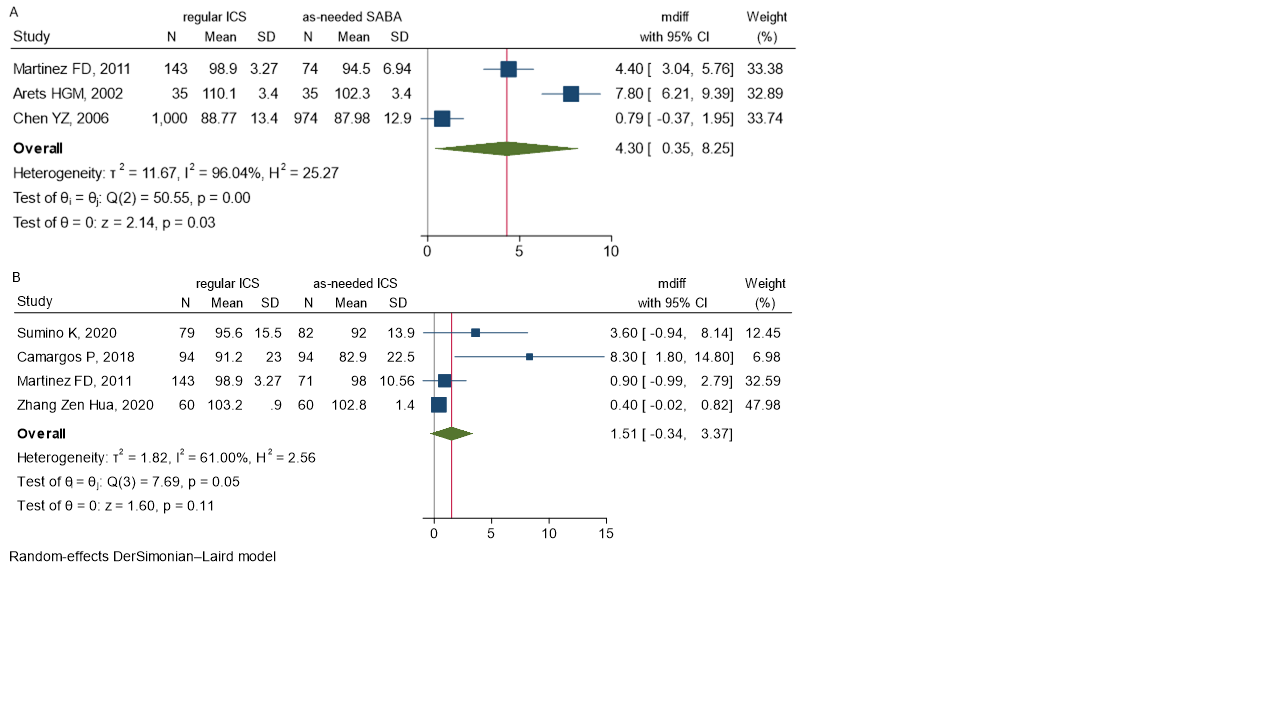
**

A) regular ICS vs. AN-SABA, and B: regular ICS vs. AN-ICS. No pooling for the comparison between LTRA and regular ICS (1 RCT).

A heterogeneity was attributable to the two treatment comparisons: 1) regular ICS vs. AN-SABA, and 2) regular ICS vs. AN-ICS, and sensitivity analyses were performed by excluding one study at a time. First, the study conducted by Chen et al. [42 in main text] for the former comparison, in which participants had mean baseline % predicted FEV_1_ <90 was excluded. The result suggested an increment of %predicted FEV_1_ from regular ICS was significantly higher than AN-SABA (MD [95%CI], 6.1% [2.74, 9.40]). Second, the study conducted by Camargos et al. [36 in main text] for the latter comparison, in which asthma step 2 was combined with asthma step 1, and previous ICS was used in more than 90% of the participants was excluded. The result suggested treatment with regular ICS and AN-ICS was similar (MD [95%CI], 0.5% [-0.1, 1.1]).

**Fig. S4.** Publication bias assessments for all relative treatment comparisons on FEV_1_ outcome


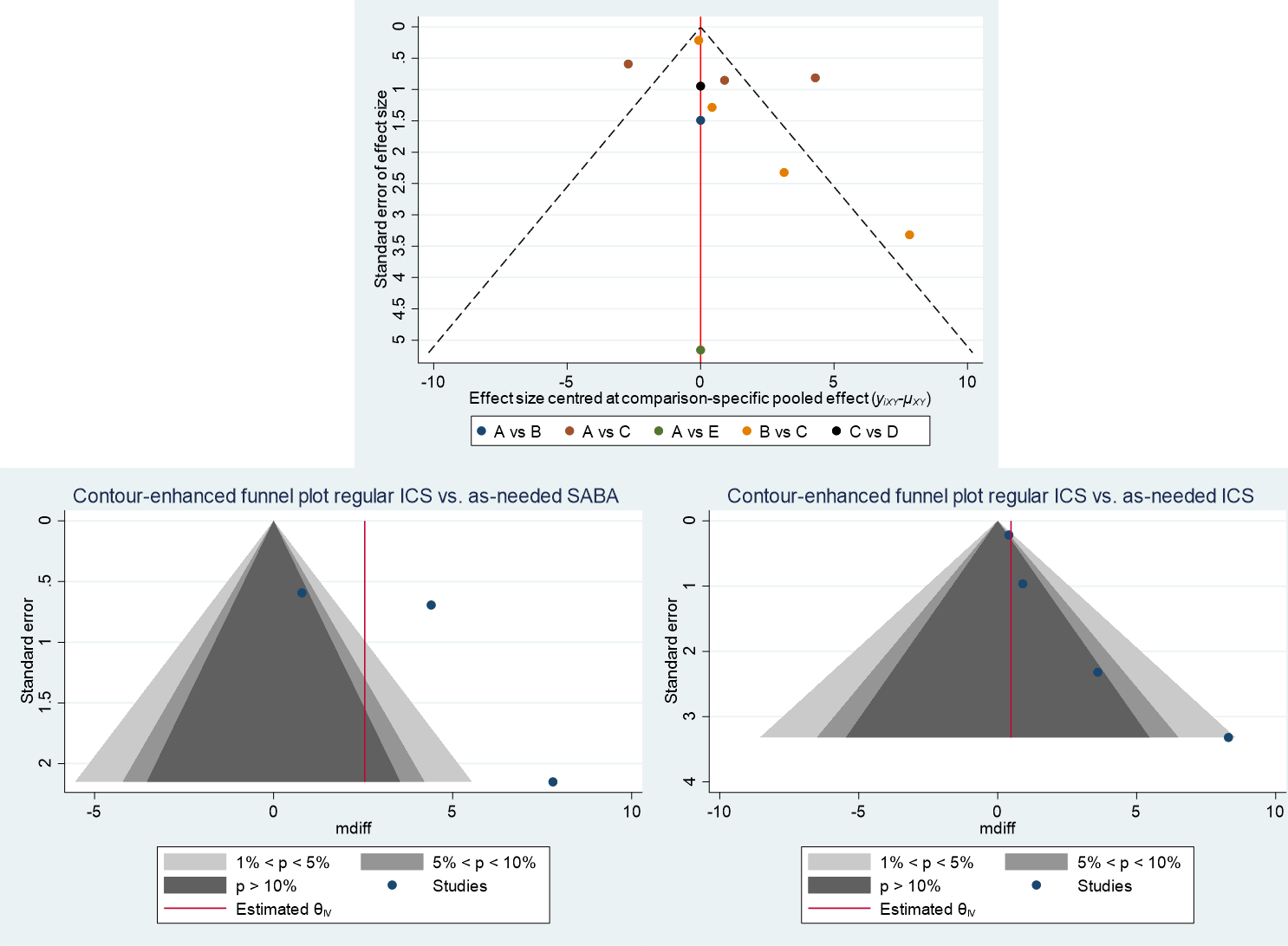


C

B

A

A) Comparison-adjusted funnel plot, A: AN-SABA, B: AN-ICS, C: regular ICS, D: LTRA, E: regular ICS/terbutaline, B) Contour-enhanced funnel plot of regular ICS vs. AN-SABA, C) Contour-enhanced funnel plot of regular ICS vs. AN-ICS

**Treatment effects in adolescents/adults**

**A) Non-severe exacerbation**

*Direct meta-analysis*

**Fig. S5.** Forest plots of non-severe exacerbation outcome

**
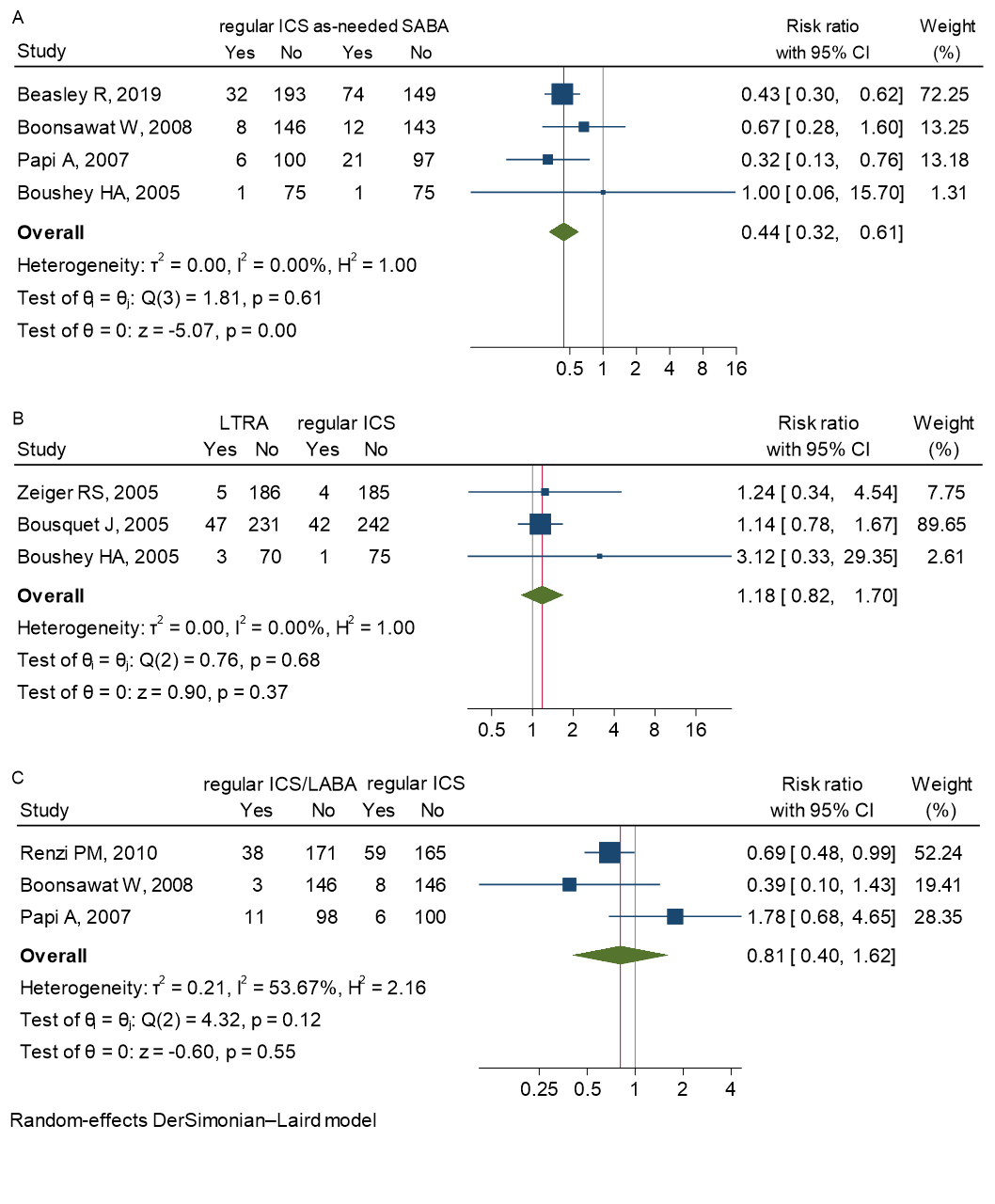
**

A) regular ICS vs. AN-SABA, B) regular ICS vs. LTRA, and C) regular ICS/LABA vs. regular ICS. No pooling for the comparison between AN-ICS/FABA and regular ICS (2 RCTs).

**B) Severe exacerbation**

*Direct meta-analysis*

**Fig. S6.** Forest plots of severe exacerbation outcome

**
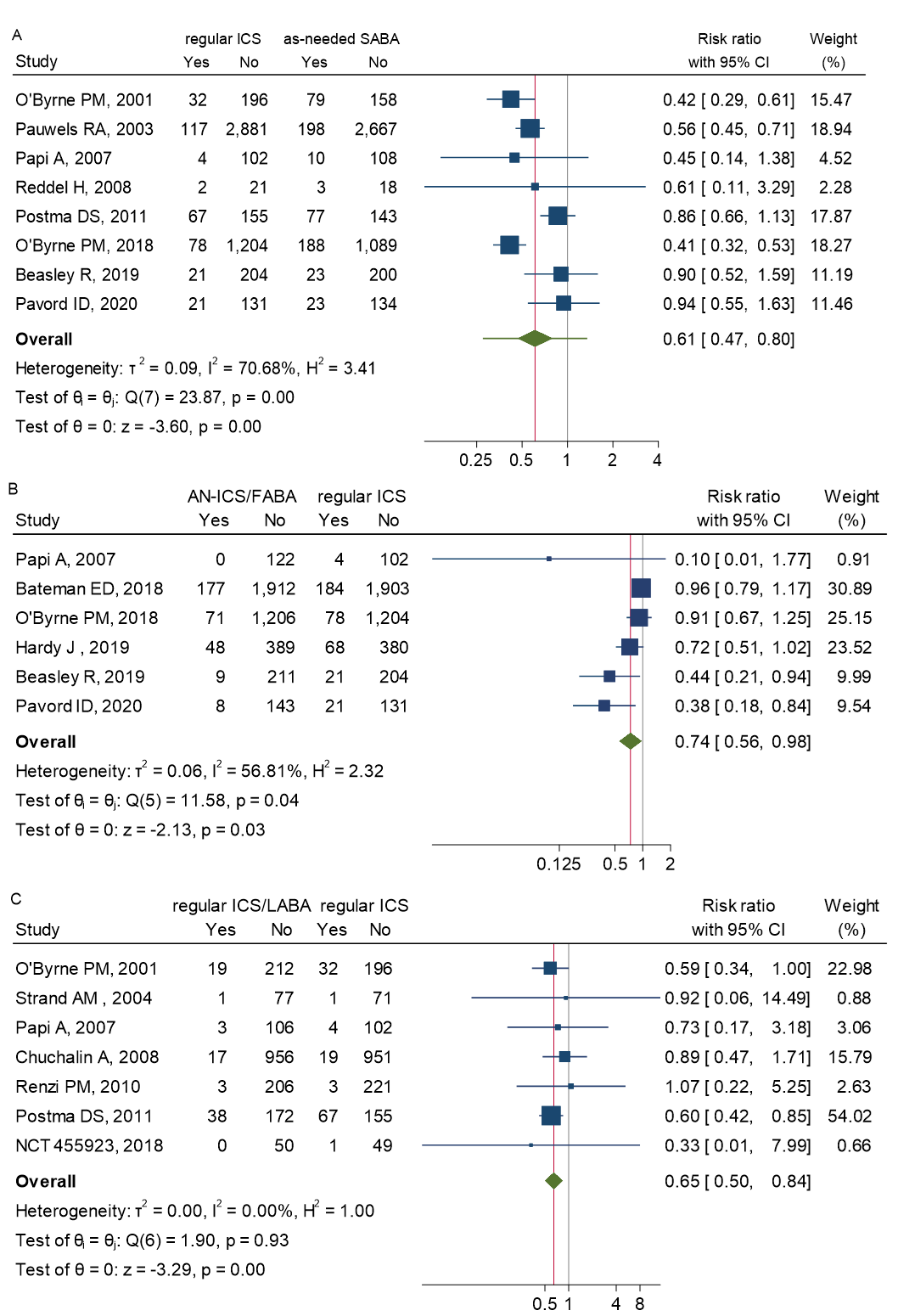
**

A) regular ICS vs. AN-SABA, B) AN-ICS/FABA vs. regular ICS, and C) regular ICS/LABA vs. regular ICS

A heterogeneity for severe exacerbation outcome in the treatment comparisons between regular ICS vs. AN-SABA, and regular ICS vs. AN-ICS/FABA was due to the differences in baseline % predicted FEV_1_, ACQ-5, and proportion of exacerbation in the past year. The results of subgroup analyses suggested a greater reduction of severe exacerbation risk with regular ICS than AN-SABA in patients with baseline % predicted FEV_1_ of <90 (Fig. S7: A), and greater with AN-ICS/FABA compared to regular ICS in patients with baseline % predicted FEV_1_ of >88 (Fig. S7: B). In addition, compared with regular ICS, treatment with AN-ICS/FABA significantly reduced severe exacerbation risk in patients with baseline ACQ-5 <1.15 Fig. S8: A) and with baseline proportion of exacerbation in the past year <10% (Fig. S8: B).

**Fig. S7.** Subgroup analysis by baseline % predicted FEV_1_ on severe exacerbation outcome

A) regular ICS vs. AN-SABA and B) AN-ICS/FABA vs. regular ICS

**Fig. S8.** Subgroup analysis by baseline ACQ-5 and proportion of previous exacerbation in the past year on severe exacerbation outcome


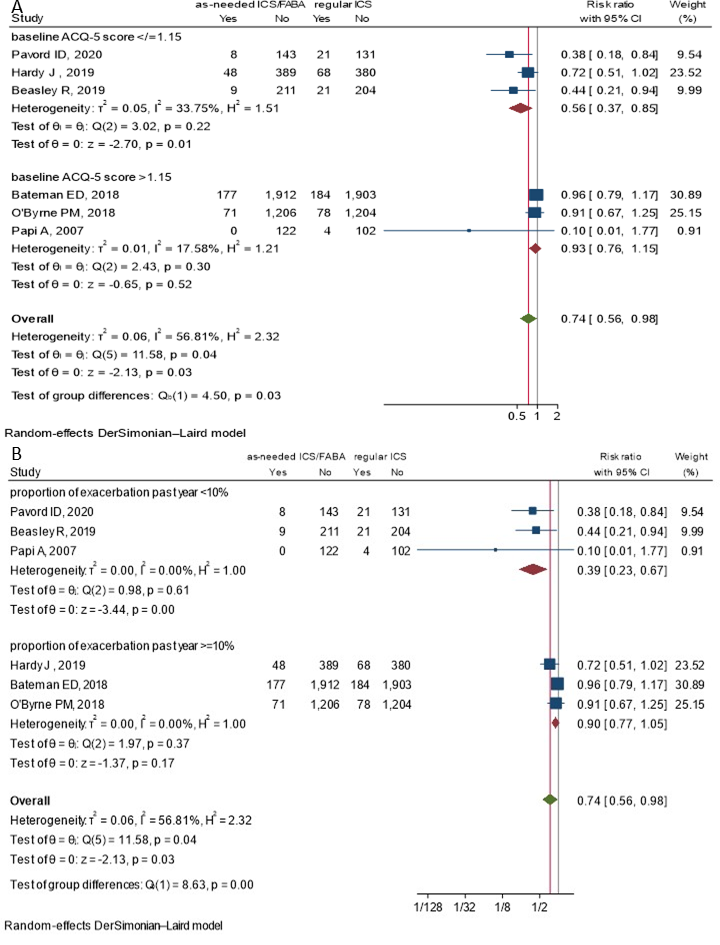


**Fig. S9.** Publication bias assessments for all relative treatment comparisons on severe exacerbation outcome


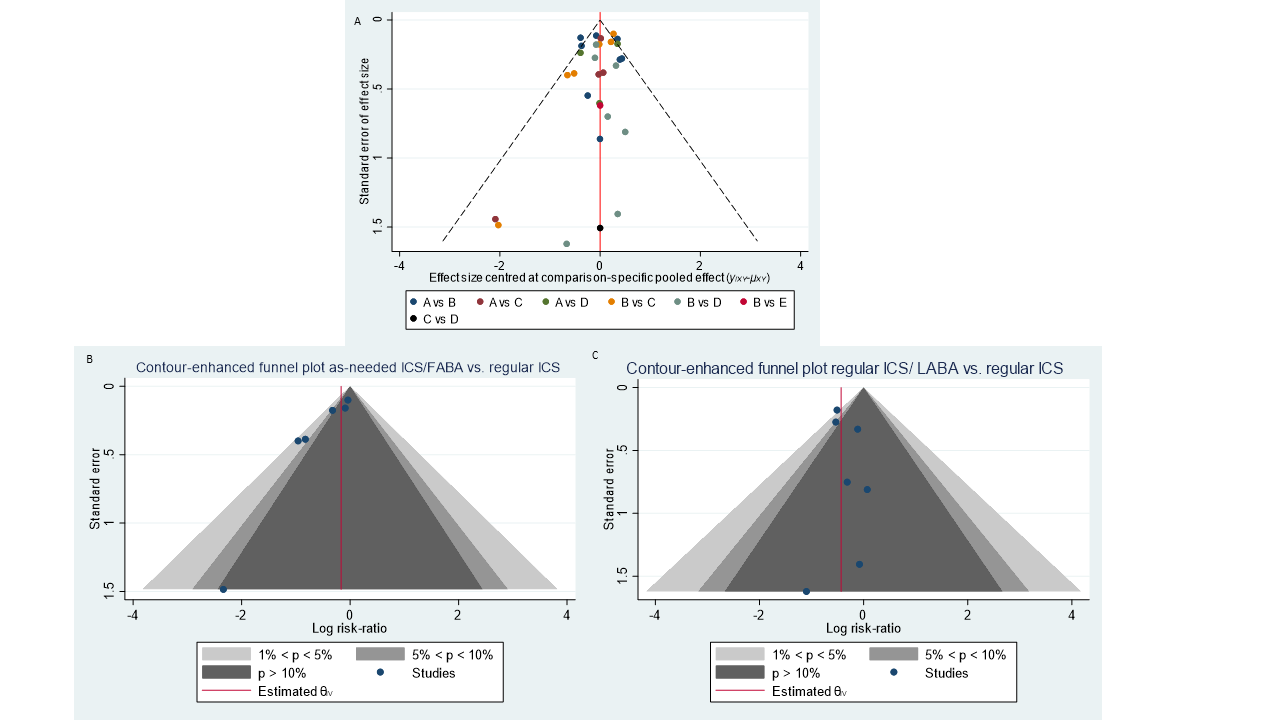


A) Comparison-adjusted funnel plot, A: AN-SABA, B: regular ICS, C: AN-ICS/FABA, D: regular ICS/LABA, E: tiotropium, B) Contour-enhanced funnel plot of AN-ICS/FABA vs. regular ICS, C) Contour-enhanced funnel plot of regular ICS/LABA vs. regular ICS

**C) Asthma symptoms**

*Direct meta-analysis*

**Fig. S10.** Forest plots of asthma symptom outcome (A-C) symptom scale, (D) ACQ-5

*
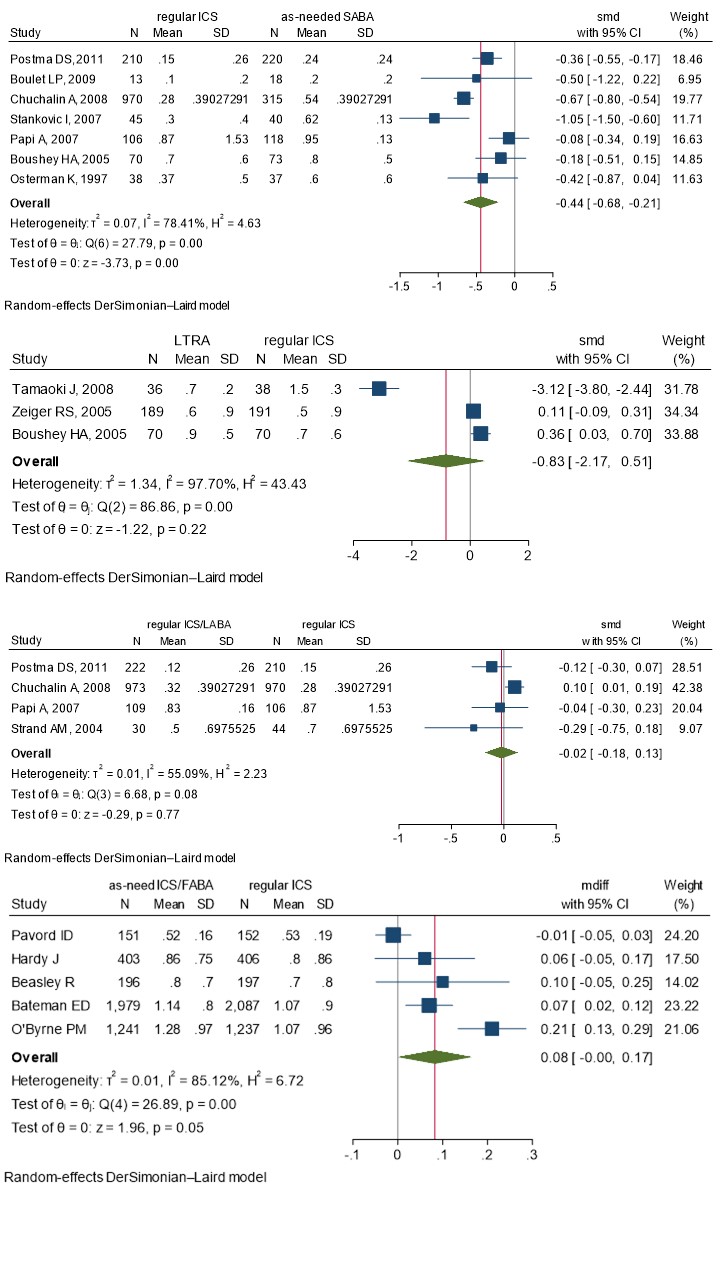
*

A

B

C

D

A) regular ICS vs. AN-SABA, B) LTRA vs. regular ICS, C) regular ICS/LABA vs. regular ICS, and D) AN-ICS/FABA vs. regular ICS

**Fig. S11.** Publication bias assessments for all relative treatment comparisons on asthma symptom outcome


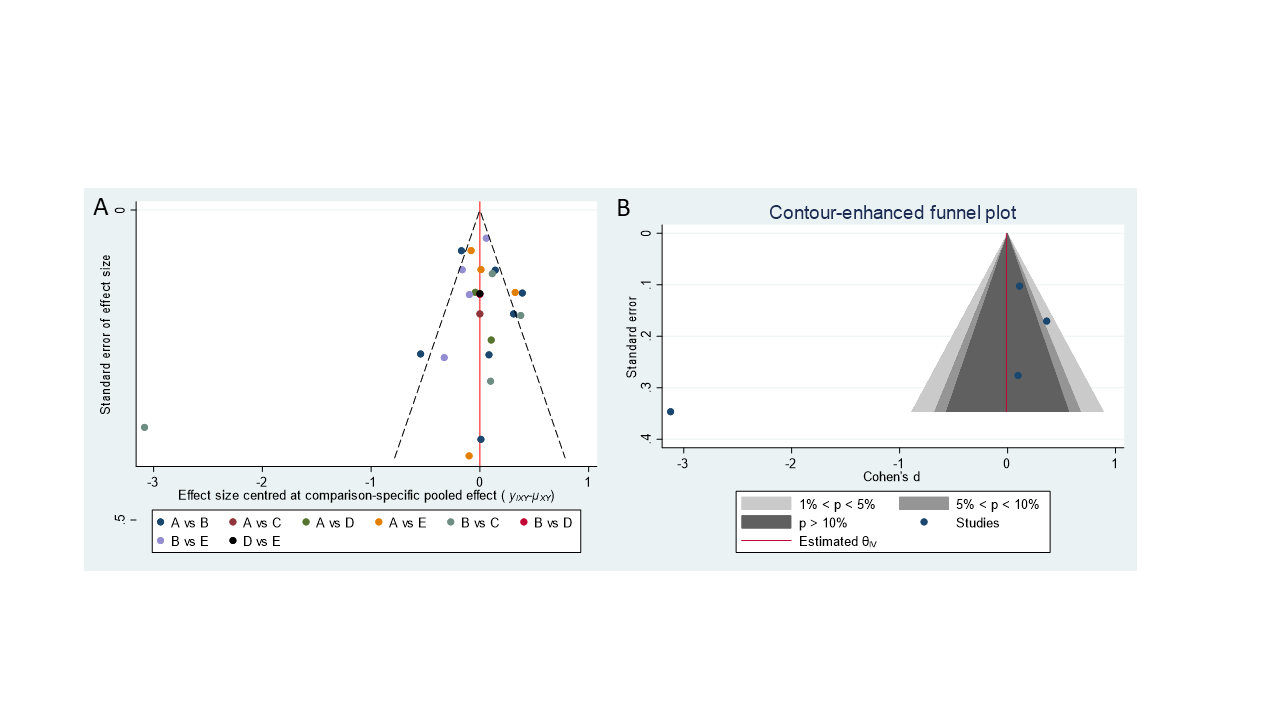
A) Comparison-adjusted funnel plot, A: as-need SABA, B: regular ICS, C: LTRA, D: as-needed ICS/FABA, E: regular ICS/LABA, B) Contour-enhanced funnel plot of LTRA vs. regular ICS.

**D) FEV_1_**

*Direct meta-analysis*

**Fig. S12.** Forest plots of % predicted FEV_1_ outcome


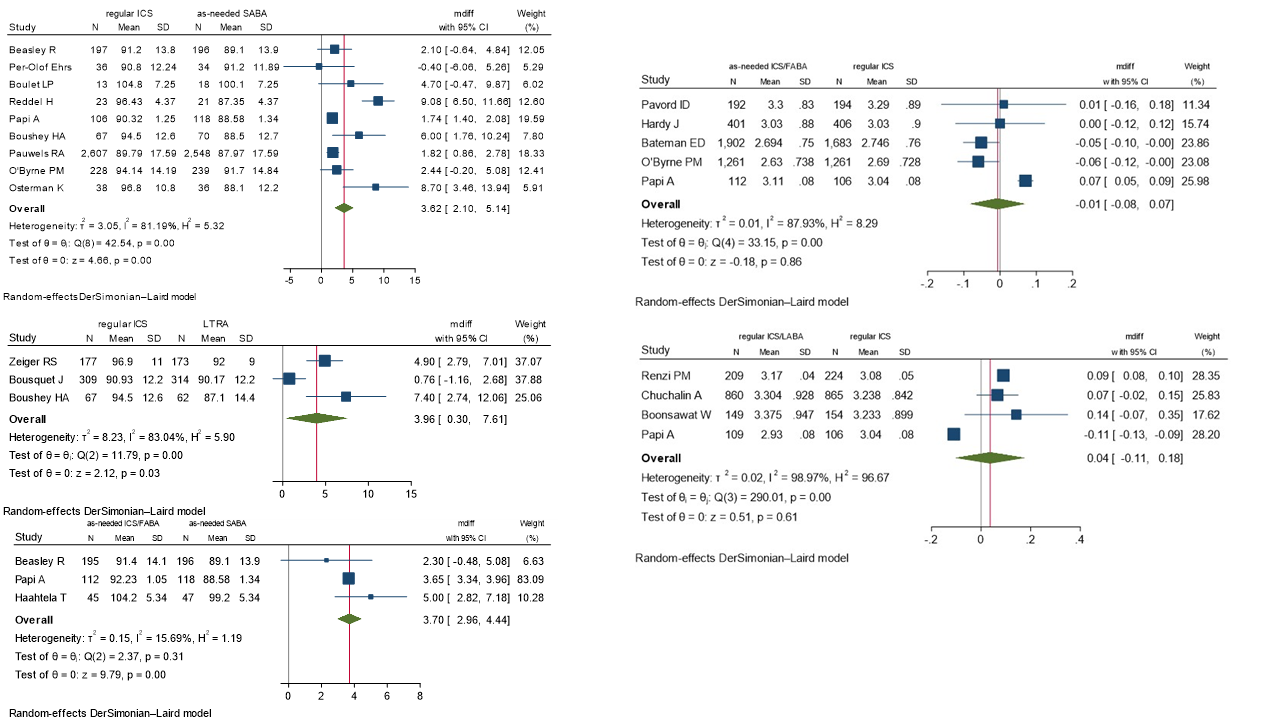


A

C

B


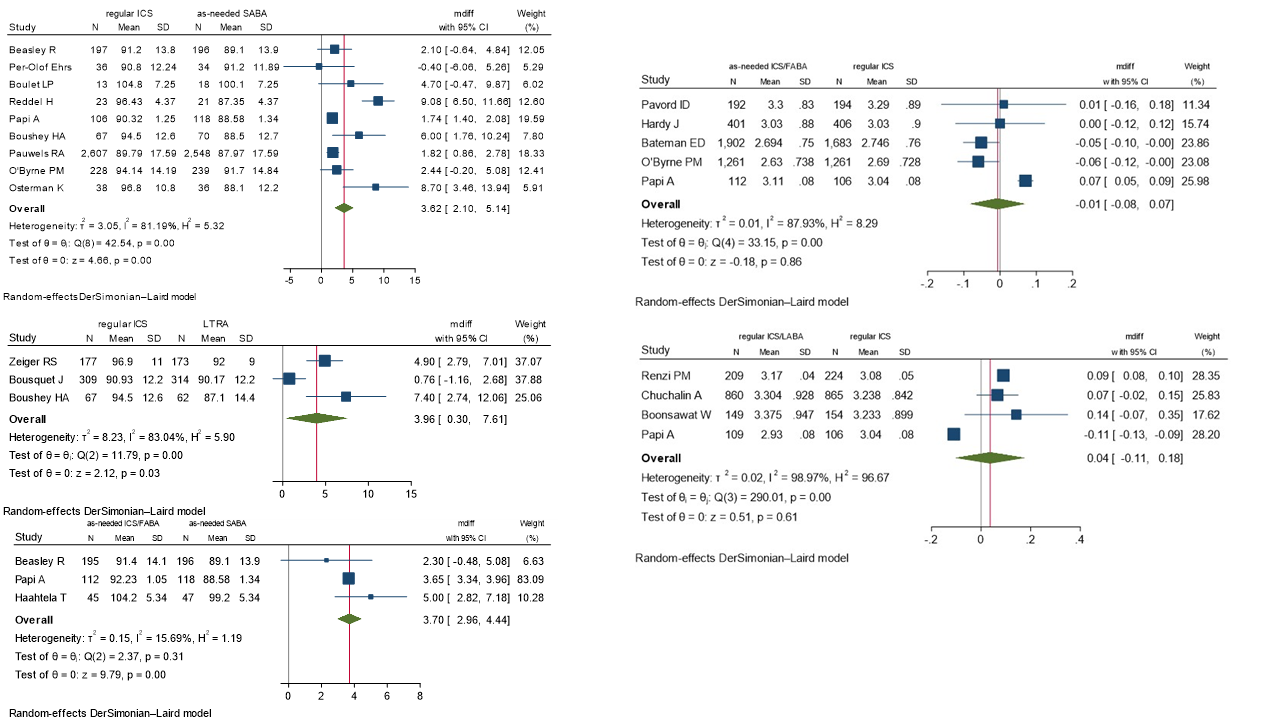


A

B

A) regular ICS vs. AN-SABA, B) LTRA vs. regular ICS, and AN-ICS/FABA vs. AN-SABA

**Forest plots of FEV_1_ (in liter) outcome**


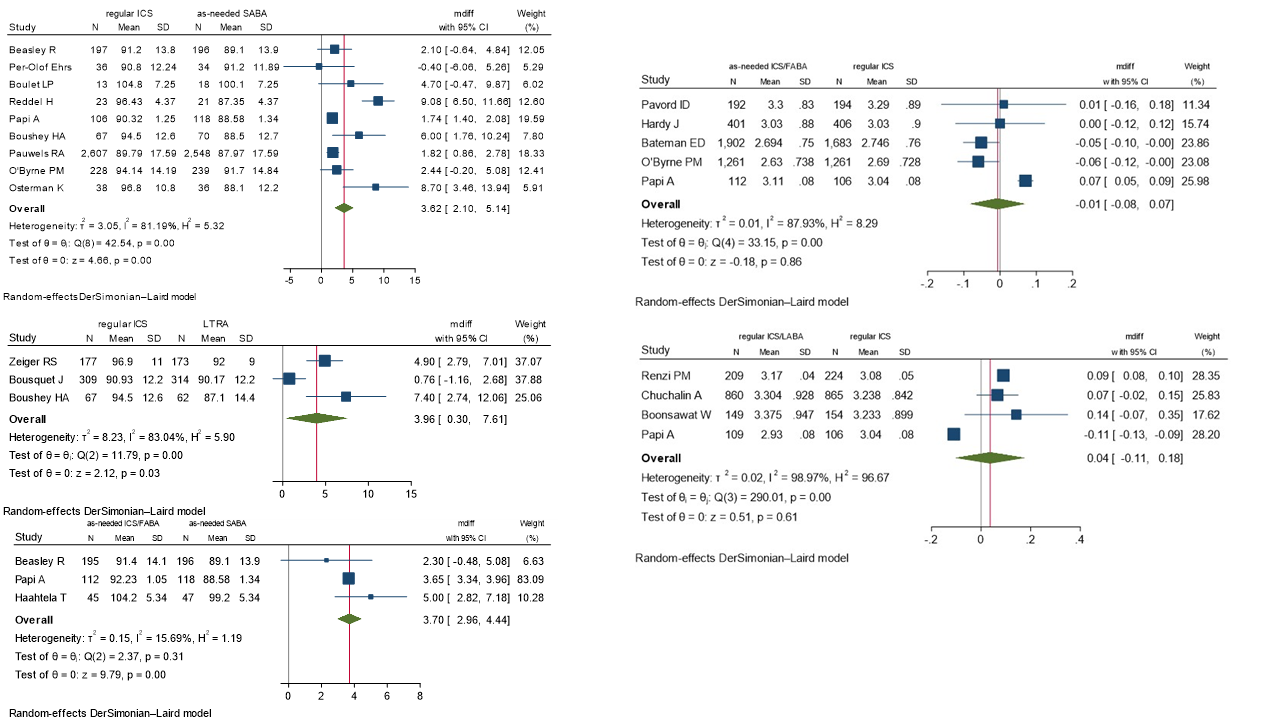


A

B

The high degree of heterogeneity for FEV_1_ outcome in the treatment comparisons between regular ICS vs. AN-SABA and regular ICS vs. AN-ICS/FABA was due to the differences in baseline asthma step and ICS use at baseline. The results of subgroup analyses suggested treatment with regular ICS resulted in a similar change of % predicted FEV_1_ compared to AN-SABA in patients with asthma step 1, but significantly better with regular ICS in patients with asthma step 2 (Figure 6.10). Despite a small magnitude of changes in FEV_1_ (in liter), treatment with AN-ICS/FABA was associated with a lesser degree of increasing FEV_1_ (in liter), compared to regular ICS in patients who did not use ICS at baseline (Figure 6.11A).

A) AN-ICS/FABA vs. regular ICS, and B) regular ICS/LABA vs. regular ICS

The high degree of heterogeneity for FEV_1_ outcome in the treatment comparisons between regular ICS vs. AN-SABA and regular ICS vs. AN-ICS/FABA was due to the differences in baseline asthma step and ICS use at baseline. The results of subgroup analyses suggested treatment with regular ICS resulted in a similar change of % predicted FEV_1_ compared to AN-SABA in patients with asthma step 1, but significantly better with regular ICS in patients with asthma step 2 (Fig. S13). Despite a small magnitude of changes in FEV_1_ (in liter), treatment with AN-ICS/FABA was associated with a lesser degree of increasing FEV_1_ (in liter), compared to regular ICS in patients who did not use ICS at baseline (Fig. S14).

**
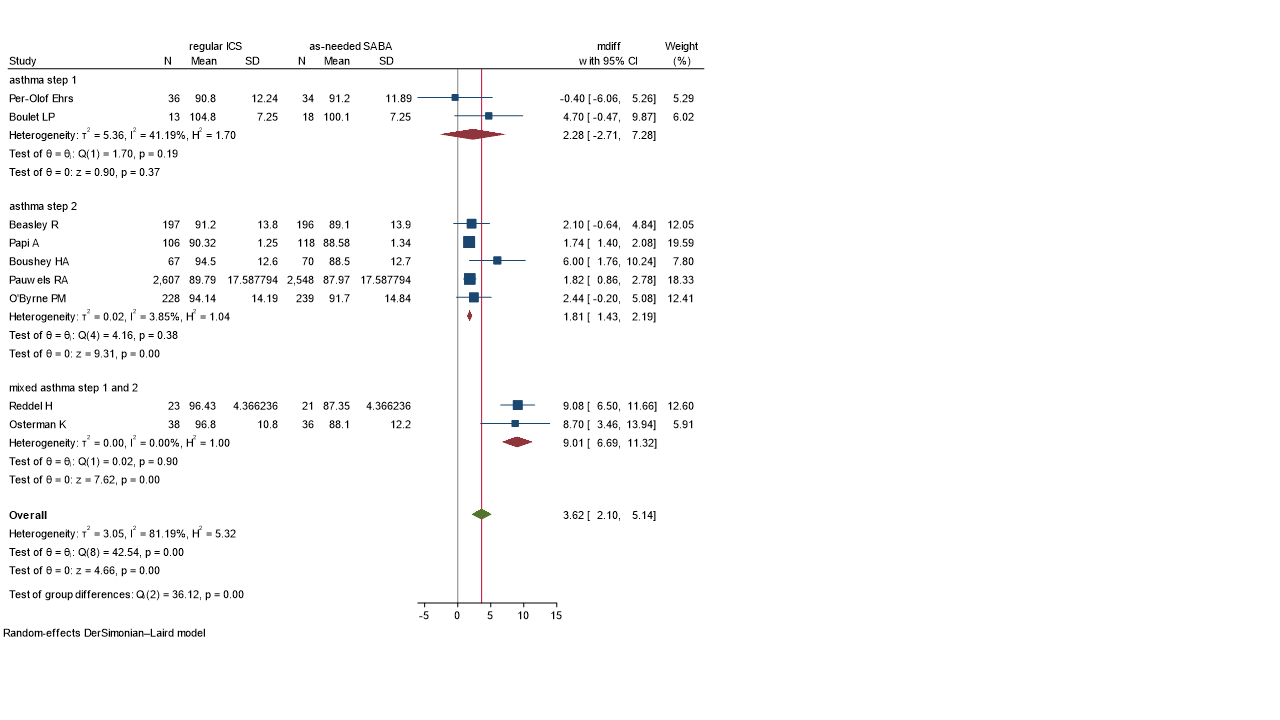
Fig. S13.** Subgroup analysis by asthma step on % predicted FEV_1_ outcome

**Fig. S14.** Subgroup analysis by baseline ICS use on FEV_1_ in liter outcome


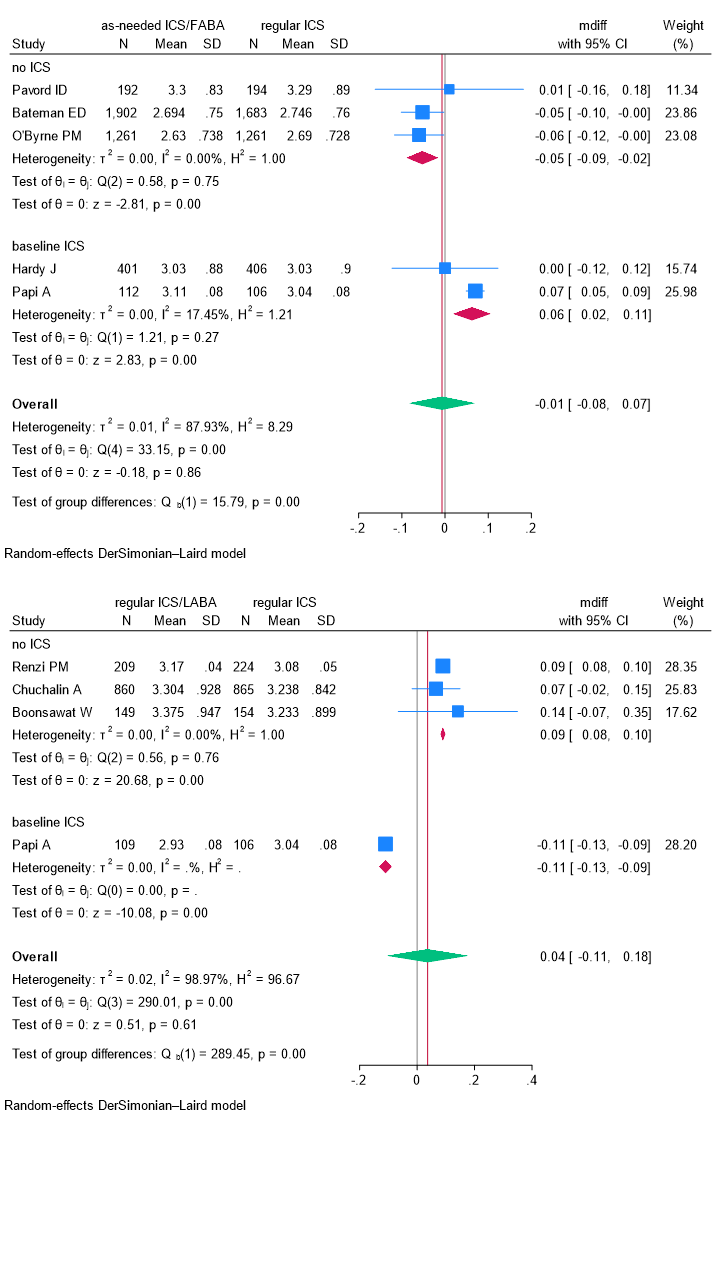


A

B

A) AN-ICS/FABA vs. regular ICS, and B) regular ICS/LABA vs. regular ICS

**Fig. S15.** Publication bias assessments for all relative treatment comparisons on % predicted FEV_1_ outcome

**
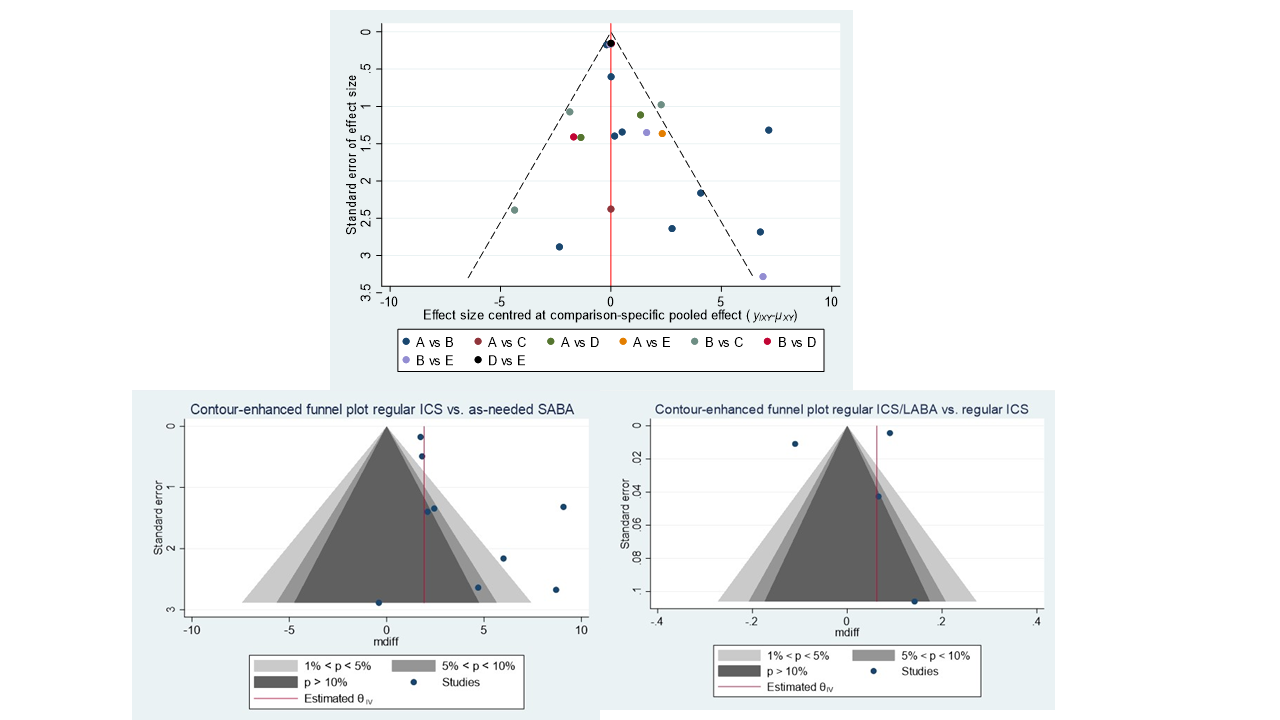
**

A

B

C

A) Comparison-adjusted funnel plot, A: AN-SABA, B: regular ICS, C: LTRA, D: AN-ICS/FABA, E: regular ICS/LABA, B) Contour-enhanced funnel plot of regular ICS vs. AN-SABA, C) Contour-enhanced funnel plot of regular ICS/LABA vs. regular ICS

**E) Asthma-specific quality-of-life**

*Direct meta-analysis*

**Fig. S16.** Forest plots of AQLQ outcome

**
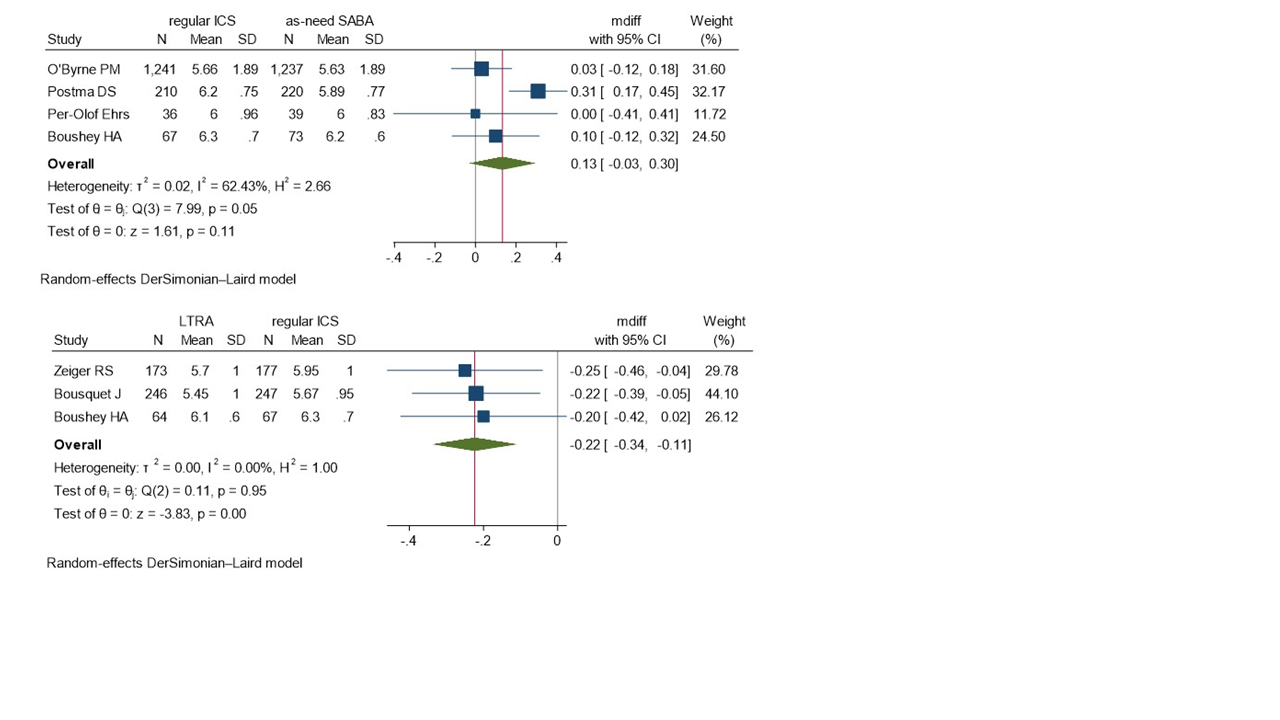
**

A) regular ICS vs. as-needed SABA, and B) LTRA vs. regular ICS

**Figure 5.14.** Subgroup analysis by age group on AQLQ outcome


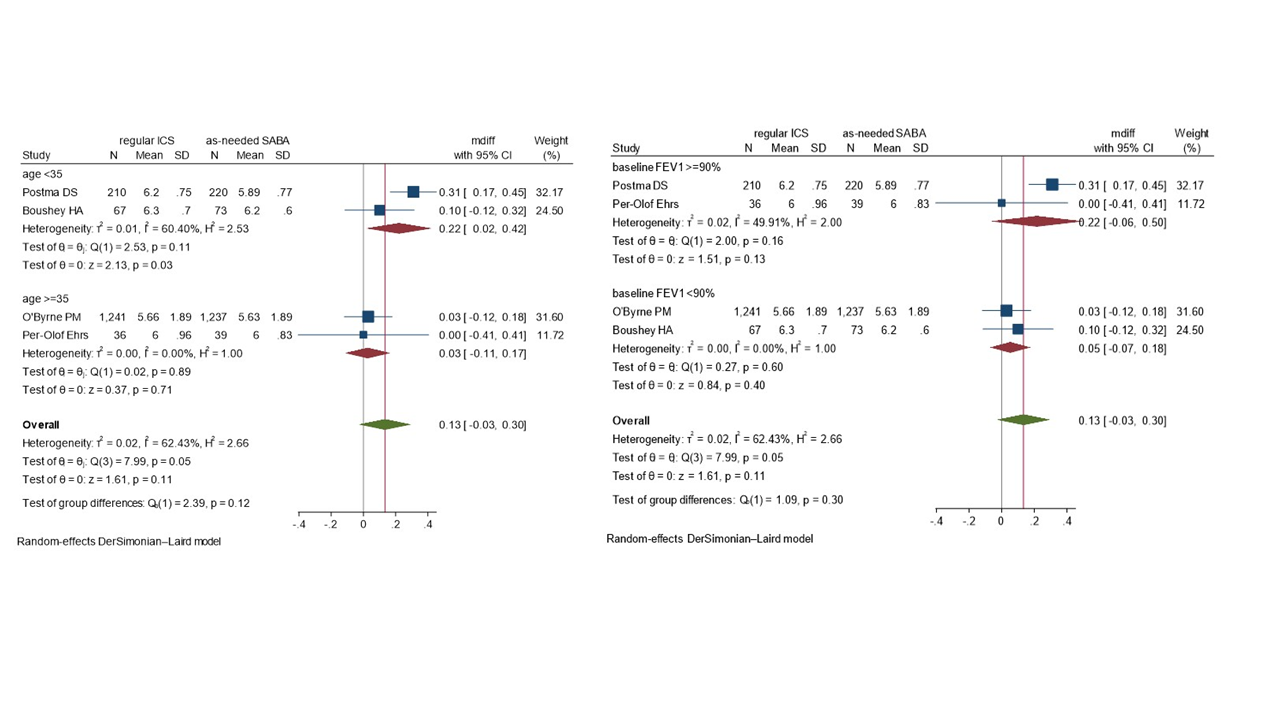
**Fig. S17.** Subgroup analysis by age group on AQLQ outcome

**Fig. S18.** Subgroup analysis by baseline % predicted FEV_1_ on AQLQ outcome


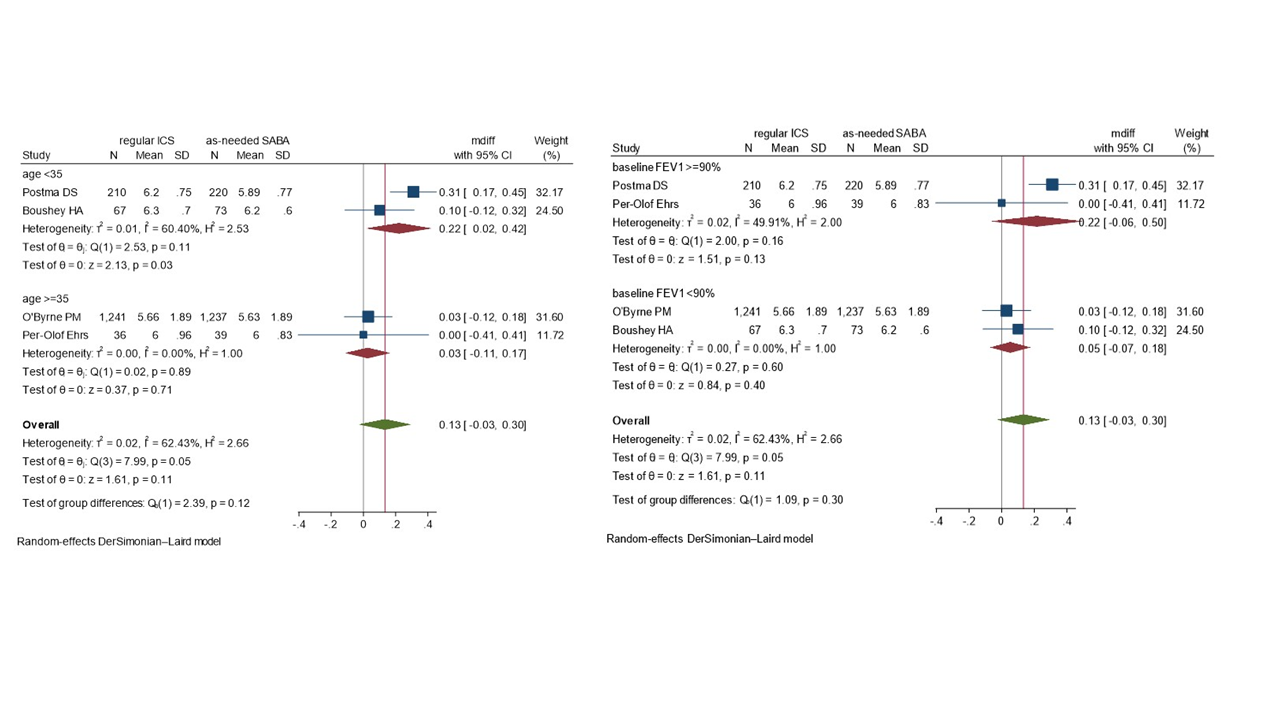


**Fig. S19.** Publication bias assessments for all relative treatment comparisons on AQLQ


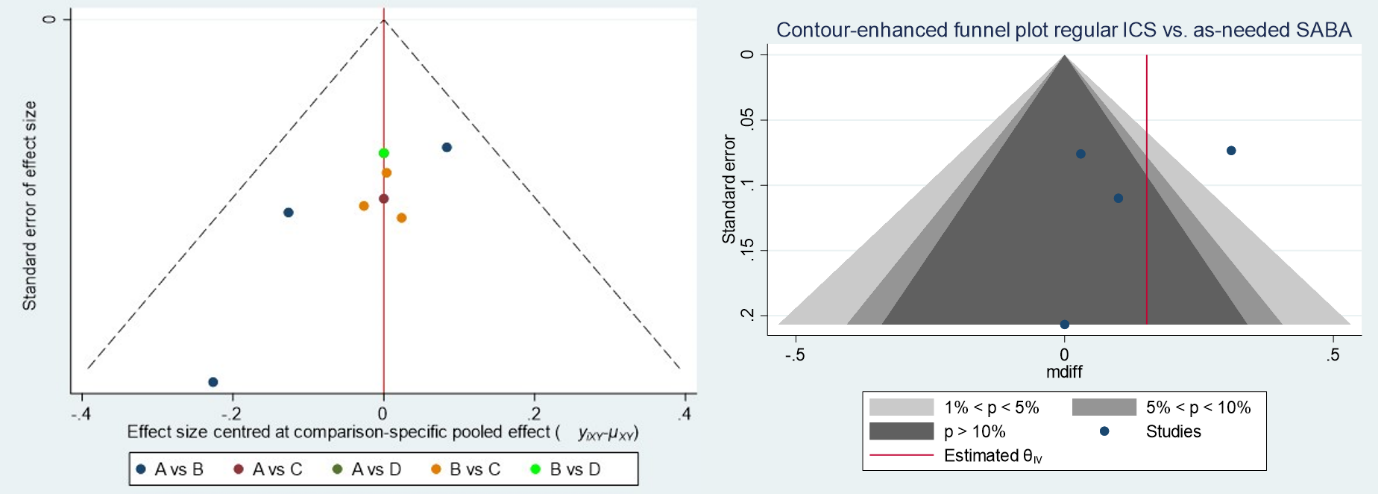


A

B

A) Comparison-adjusted funnel plot, A: AN-SABA, B: regular ICS, C: LTRA, D: regular ICS/LABA, B) Contour-enhanced funnel plot of regular ICS vs. AN-SABA

**F) Severe adverse events**

*Direct meta-analysis*

**Fig. S20.** Forest plots of severe adverse events outcome

**
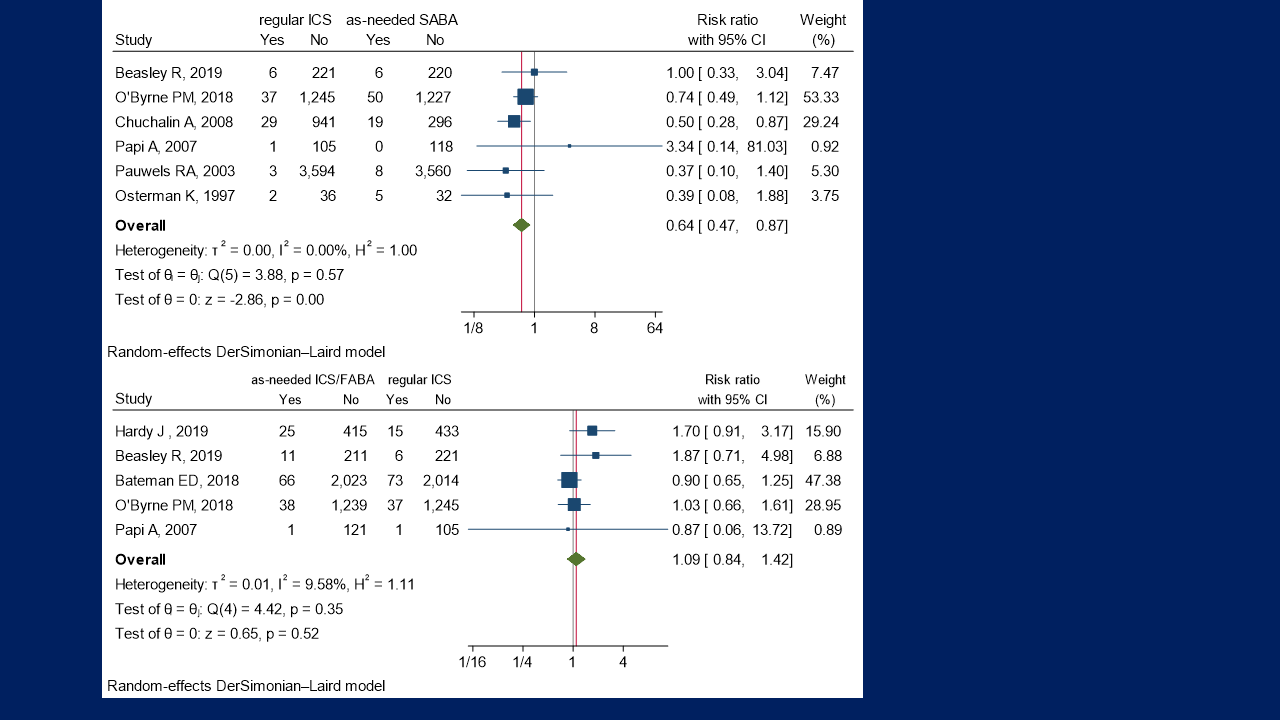
**

A

B

A) regular ICS vs. AN-SABA, and B) AN-ICS/FABA vs. regular ICS
