# Supplementary material for: Regular versus as-needed treatments for mild asthma in children, adolescents, and adults: a systematic review and network meta-analysis
Source: BMC Med. 2025 Jan 21;23:21. doi: 10.1186/s12916-025-03847-z (PMC11752773; doi:10.1186/s12916-025-03847-z)
Supplement: Supplementary file 2 — Additional file 2. The extracted data for analysis. [file 12916_2025_3847_MOESM2_ESM.docx]

**Additional file 2**

**Data extracted from included studies for including in the analyses**

**Table 1** Data extracted for pooling exacerbation outcome in children

| **First author** | **n** | **Treatment compared** | **Code** | **Drug name** | **Dose** | **Frequency** | **No. of event** | **No. of non-event** |
| --- | --- | --- | --- | --- | --- | --- | --- | --- |
| Garcia ML, 2005 | 495 | LTRA | D | montelukast | 5 | 1 | 155 | 327 |
|  | 499 | R-ICS | C | fluticasone | 100 | 2 | 124 | 360 |
| Becker A, 2006 | 120 | LTRA | D | montelukast | 5 | 1 | 42 | 79 |
|  | 119 | R-ICS | C | BDP | 200 | 2 | 28 | 91 |
|  | 121 | AN-SABA | A | SABA |  |  | 30 | 90 |
| Martinez FD, 2011 | 143 | R-ICS | C | BDP | 40 | 2 | 42 | 101 |
|  | 71 | AN-ICS | B | BDP | 80 |  | 36 | 38 |
|  | 74 | AN-SABA | A | albuterol | 180 |  | 25 | 46 |
| Shah MB, 2014 | 30 | LTRA | D | montelukast | 5 | 1 | 15 | 15 |
|  | 30 | R-ICS | C | BUD | 200 | 2 | 19 | 11 |
| Camargos P, 2018 | 94 | R-ICS | C | BDP | 250 | 2 | 10 | 84 |
|  | 94 | AN-ICS | B | BDP | 250 |  | 7 | 87 |
| Sumino K, 2020 | 103 | R-ICS | C | BDP | 40 | 2 | 19 | 84 |
|  | 103 | AN-ICS | B | BDP | 80 |  | 22 | 81 |

Abbreviations R: regular, AN: as-needed, BUD: budesonide, BDP: beclomethasone dipropionate

**Table 2** Data extracted for pooling FEV_1_ outcome in children

| **First author** | **n** | **Treatment compared** | **Code** | **Drug name** | **dose** | **frequency** | **Mean** | **SD** |
| --- | --- | --- | --- | --- | --- | --- | --- | --- |
| Sumino K, 2020 | 82 | R-ICS | C | BDP | 40 | 2 | 95.6 | 13.90 |
|  | 79 | AN-ICS | B | BDP | 80 |  | 92 | 15.50 |
| Camargos P, 2018 | 94 | R-ICS | C | BDP | 250 | 2 | 82.9 | 23.00 |
|  | 94 | AN-ICS | B | BDP | 250 |  | 91.2 | 22.50 |
| Martinez FD, 2011 | 74 | R-ICS | C | BDP | 40 | 2 | 94.5 | 6.94 |
|  | 71 | AN-SABA | B | albuterol | 180 | 0 | 98 | 10.56 |
| Garcia ML, 2005 | 439 | LTRA | D | montelukast | 5 | 1 | 87.2 | 15.70 |
|  | 442 | R-ICS | C | fluticasone | 100 | 2 | 90.4 | 12.10 |
| Arets HGM, 2002 | 5 | R-ICS | C | fluticasone | 125 | 2 | 102.3 | 3.40 |
|  | 5 | AN-SABA | A | SABA | 125 |  | 110.1 | 3.40 |
| Waalkens HJ, 1991 | 12 | R-ICS | C | BUD/terbutaline | 200 | 2 | 93.3 | 12.37 |
|  | 15 | AN-SABA | A | terbutaline | 500 |  | 90.2 | 14.41 |
| Zhang Zen Hua, 2020 | 60 | R-ICS | C | BUD | 200 | 1 | 102.8 | 1.20 |
|  | 60 | AN-ICS | B | BUD | 200 |  | 103.2 | 1.40 |
| Chen YZ, 2006 | 974 | R-ICS | C | BUD | 200 | 1 | 87.98 | 12.90 |
|  | 1000 | AN-SABA | A | SABA | 200 |  | 88.77 | 13.40 |

Abbreviations R: regular, AN: as-needed, BUD: budesonide, BDP: beclomethasone dipropionate

**Table 3** Data for pooling adverse event outcome in children

| **First author** | **n** | **Treatment compared** | **Code** | **Drug name** | **Dose** | **Frequency** | **No. of event** | **No. of non-event** |
| --- | --- | --- | --- | --- | --- | --- | --- | --- |
| Sumino K, 2020 | 103 | AN-ICS | B | BDP | 80 |  | 4 | 99 |
|  | 103 | R-ICS | C | BDP | 40 | 2 | 6 | 97 |
| Shah MB, 2014 | 30 | LTRA | D | montelukast | 5 | 1 | 2 | 28 |
|  | 30 | R-ICS | C | BDP | 200 | 2 | 0 | 30 |
| Visitsunthorn N, 2011 | 15 | AN-SABA | A | SABA |  |  | 0 | 15 |
|  | 14 | LTRA | D | montelukast | 5 | 1 | 0 | 14 |
| Becker A, 2006 | 119 | R-ICS | C | BDP | 200 | 2 | 28 | 91 |
|  | 121 | AN-SABA | A | SABA |  |  | 29 | 92 |
|  | 120 | LTRA | D | montelukast | 5 | 1 | 28 | 92 |
| Garcia ML, 2005 | 499 | R-ICS | C | fluticasone | 100 | 2 | 6 | 493 |
|  | 495 | LTRA | D | montelukast | 5 | 1 | 11 | 484 |

Abbreviations R: regular, AN: as-needed, BDP: beclomethasone dipropionate

**Table 4** Data extracted for pooling exacerbation outcome in adolescents/adults

| **First author** | **n** | **Treatment compared** | **Code** | **Drug name** |  | **dose** | **Frequency** | **No. of event** | **No. of non-event** |
| --- | --- | --- | --- | --- | --- | --- | --- | --- | --- |
| Beasley R, 2019 | 220 | AN ICS/FABA | D | BUD/form |  | 200 |  | 37 | 183 |
|  | 223 | R-ICS | B | BUD |  | 100 | 2 | 32 | 193 |
|  | 225 | AN-SABA | A | albuterol |  | 200 |  | 74 | 149 |
| Lazarus SC, 2019 | 221 | Tiotropium | F | tiotropium |  | 220 | 1 | 5 | 206 |
|  | 221 | R-ICS | B | mometasone |  | 5 | 2 | 3 | 208 |
| Renzi PM, 2010 | 263 | R-ICS/LABA | E | Fluticasone/salm |  | 100 | 2 | 38 | 171 |
|  | 253 | R-ICS | B | Fluticasone |  | 100 | 2 | 59 | 165 |
| Boonsawat W, 2008 | 155 | R-ICS/LABA | E | Fluticasone/salm |  | 100 | 1 | 3 | 146 |
|  | 154 | R-ICS | B | Fluticasone |  | 100 | 1 | 8 | 146 |
|  | 149 | AN-SABA | A | SABA |  | 100 |  | 12 | 143 |
| Papi A, 2007 | 122 | R-ICS/LABA | E | BDP/albuterol |  | 250 | 2 | 11 | 98 |
|  | 109 | AN ICS/SABA | D | BDP/albuterol |  | 250 |  | 6 | 116 |
|  | 118 | R-ICS | B | BDP |  | 100 | 2 | 6 | 100 |
|  | 106 | AN-SABA | A | albuterol |  |  |  | 21 | 97 |
| Zeiger RS, 2005 | 191 | LTRA | C | Montelukast |  | 10 | 1 | 0 | 191 |
|  | 189 | R-ICS | B | Fluticasone |  | 44 | 2 | 0 | . |
| Bousquet J, 2005 | 320 | LTRA | C | Montelukast |  | 100 | 2 | 47 | 231 |
|  | 325 | R-ICS | B | Fluticasone |  | 100 | 2 | 42 | 242 |
| Boushey HA, 2005 | 73 | LTRA | C | zafirlukast |  | 20 | 2 | 3 | 70 |
|  | 76 | R-ICS | B | BUD |  | 200 | 2 | 1 | 75 |
|  | 76 | AN-SABA | A | SABA |  |  |  | 1 | 75 |
| NCT 1316380, 2015 | 309 | Tiotropium | F | tiotropium |  | 5 | 1 | 34 | 275 |
|  | 155 | R-ICS | B | ICS |  |  |  | 22 | 133 |

Abbreviations R: regular, AN: as-needed, BDP: beclomethasone diproprionate, BUD: budesonide

**Table 5** Data extracted for pooling severe exacerbation outcome in adolescents/adults

| **First author** | **n** | **Treatment compared** | **Code** | | **Drug name** | **dose** | **Freq** | **No. of event** | **No. of non-event** |
| --- | --- | --- | --- | --- | --- | --- | --- | --- | --- |
| Pavord ID, 2020 | 49 | AN-SABA | | A | albuterol | 100 |  | 23 | 134 |
|  | 62 | R-ICS | | B | BUD | 200 | 2 | 21 | 131 |
|  | 72 | AN ICS/FABA | | D | BUD/form | 200 |  | 8 | 143 |
| Hardy J, 2019 | 437 | R-ICS | | B | BUD | 200 | 2 | 68 | 380 |
|  | 448 | AN ICS/FABA | | D | BUD/form | 200 |  | 48 | 389 |
| Beasley R, 2019 | 225 | AN-SABA | | A | albuterol | 100 |  | 23 | 200 |
|  | 223 | R-ICS | | B | BUD | 200 | 2 | 21 | 204 |
|  | 220 | AN ICS/FABA | | D | BUD/form | 200 |  | 9 | 211 |
| Bateman ED, 2018 | 2089 | R-ICS | | B | BUD | 200 | 2 | 184 | 1903 |
|  | 2087 | AN ICS/FABA | | D | BUD/form | 200 |  | 177 | 1912 |
| O'Byrne PM, 2018 | 1277 | AN-SABA | | A | terbutaline | 500 |  | 188 | 1089 |
|  | 1277 | R-ICS | | B | BUD | 200 | 2 | 78 | 1204 |
|  | 1282 | AN ICS/FABA | | D | BUD/form | 200 |  | 71 | 1206 |
| Postma DS, 2011 | 210 | AN-SABA | | A | SABA | 100 | 2 | 77 | 143 |
|  | 222 | R-ICS | | B | ciclesonide | 160 | 1 | 67 | 155 |
|  | 220 | R-ICS/LABA | | E | Fluticasone/salm | 100 | 2 | 38 | 172 |
| Renzi PM, 2010 | 253 | R-ICS | | B | Fluticasone | 100 | 2 | 3 | 221 |
|  | 263 | R-ICS/LABA | | E | Fluticasone/salm | 100 | 2 | 3 | 206 |
| Reddel H, 2008 | 21 | AN-SABA | | A | SABA | 125 | 2 | 3 | 18 |
|  | 23 | R-ICS | | B | Fluticasone | 125 | 2 | 2 | 21 |
| Chuchalin A, 2008 | 973 | R-ICS | | B | Fluticasone | 100 | 2 | 19 | 951 |
|  | 970 | R-ICS/LABA | | E | Fluticasone/salm | 100 | 1 | 17 | 956 |
| Papi A, 2007 | 106 | AN-SABA | | A | Albuterol | 100 |  | 10 | 108 |
|  | 118 | R-ICS | | B | BDP | 250 | 2 | 4 | 102 |
|  | 122 | AN ICS/SABA | | D | BDP/albuterol | 250 |  | 0 | 122 |
|  | 109 | R-ICS/LABA | | E | BDP/albuterol | 250 | 2 | 3 | 106 |
| Strand AM, 2004 | 72 | R-ICS | | B | Fluticasone | 100 | 2 | 1 | 71 |
|  | 78 | R-ICS/LABA | | E | Fluticasone/salm | 100 | 2 | 1 | 77 |
| Pauwels RA, 2003 | 2998 | R-ICS | | B | BUD | 400 | 1 | 117 | 2881 |
|  | 2865 | AN-SABA | | A | SABA |  |  | 198 | 2667 |
| O'Byrne PM, 2001 | 239 | AN-SABA | | A | SABA | 100 | 2 | 79 | 158 |
|  | 231 | R-ICS | | B | BUD | 100 | 2 | 32 | 196 |
|  | 228 | R ICS/FABA | | E | BUD/form | 100 | 2 | 19 | 212 |
| NCT 1316380, 2015 | 309 | AN-SABA | | A | SABA |  |  | 4 | 151 |
|  | 155 | Tiotropium | | F | Tiotropium | 5 | 1 | 7 | 302 |
| NCT 455923, 2018 | 50 | R-ICS | | B | Fluticasone | 100 | 2 | 1 | 49 |
|  | 50 | R-ICS/LABA | | E | Fluticasone/salm | 100 | 2 | 0 | 50 |

Abbreviations R: regular, AN: as-needed BDP: beclomethasone diproprionate, BUD: budesonide, Form: formoterol, Salm: salmeterol

**Table 6** Data extracted for pooling asthma symptom score outcome in adolescents/adults

| **First author** | **Year** | **Treatment compared** | **Code** | **ACQ-5 mean** | **ACQ-5 SD** | **Symptom score mean** | **Symptom score SD** |
| --- | --- | --- | --- | --- | --- | --- | --- |
| Pavord ID, 2020 | **n** | R-ICS | B | 0.53 | 0.19 | . | . |
|  | 72 | AN ICS/FABA | D | 0.52 | 0.16 | . | . |
|  | 62 | AN-SABA | A | 0.16 | 0.16 | . | . |
| Hardy J, 2019 | 49 | AN ICS/FABA | D | 0.86 | 0.75 | . | . |
|  | 437 | R-ICS | B | 0.8 | 0.86 | . | . |
| Beasley R, 2019 | 448 | AN ICS/FABA | D | 0.8 | 0.7 | . | . |
|  | 225 | R-ICS | B | 0.7 | 0.8 | . | . |
|  | 220 | AN-SABA | A | 0.9 | 0.9 | . | . |
| Bateman ED, 2018 | 223 | R-ICS | B | 1.07 | 0.9 | . | . |
|  | 2087 | AN ICS/FABA | D | 1.14 | 0.8 | . | . |
| O'Byrne PM, 2018 | 2089 | AN-SABA | A | 1.37 | 0.95 | . | . |
|  | 1277 | AN ICS/FABA | D | 1.28 | 0.97 | . | . |
|  | 1277 | R-ICS | B | 1.07 | 0.96 | . | . |
| Postma DS, 2011 | 1282 | AN-SABA | A | . | . | 0.24 | 0.24 |
|  | 220 | R-ICS/LABA | E | . | . | 0.12 | 0.26 |
|  | 222 | R-ICS | B | . | . | 0.15 | 0.26 |
| Boulet LP, 2009 | 210 | AN-SABA | A | . | . | 0.2 | 0.2 |
|  | 33 | R-ICS | B | . | . | 0.1 | 0.2 |
| Tamaoki J, 2008 | 24 | LTRA | C | . | . | 0.7 | 0.2 |
|  | 36 | R-ICS | B | . | . | 1.5 | 0.3 |
| Chuchalin A, 2008 | 38 | R-ICS/LABA | E | . | . | 0.32 | 0.39 |
|  | 973 | R-ICS | B | . | . | 0.28 | 0.39 |
|  | 970 | AN-SABA | A | . | . | 0.54 | 0.39 |
| Stankovic I, 2007 | 315 | R-ICS | B | . | . | 0.3 | 0.4 |
|  | 40 | AN-SABA | A | . | . | 0.62 | 0.13 |
| Papi A, 2007 | 45 | R-ICS/SABA | E | . | . | 0.83 | 0.16 |
|  | 118 | R-ICS | B | . | . | 0.87 | 1.53 |
|  | 109 | AN-SABA | A | . | . | 0.95 | 0.13 |
|  | 106 | AN-ICS/SABA | D | . | . | 0.62 | 0.12 |
| Haahtela T, 2006 | 122 | AN ICS/FABA | D | . | . | 1.29 | 0.24 |
|  | 45 | AN-SABA | A | . | . | 1.36 | 0.24 |
| Zeiger RS, 2005 | 47 | LTRA | C | . | . | 0.6 | 0.9 |
|  | 191 | R-ICS | B | . | . | 0.5 | 0.9 |
| Boushey HA, 2005 | 189 | LTRA | C | . | . | 0.9 | 0.5 |
|  | 76 | AN-SABA | A | . | . | 0.8 | 0.5 |
|  | 76 | R-ICS | B | . | . | 0.7 | 0.6 |
| Strand AM, 2004 | 73 | R-ICS | B | . | . | 0.7 | 0.7 |
|  | 78 | R-ICS/LABA | E | . | . | 0.5 | 0.7 |
| Osterman K, 1997 | 72 | AN-SABA | A | . | . | 0.6 | 0.6 |
|  | 38 | R-ICS | B | . | . | 0.37 | 0.5 |
| NCT4442559, 2022 | 37 | LTRA | C | . | . | 0.16 | 0.35 |
|  | 29 | R-ICS | B | . | . | 0.13 | 0.27 |

**Table 7** Data extracted for pooling FEV_1_ outcome in adolescents/adults

| **First author** | **n** | **Treatment compared** | **Code** | | **FEV_1_**  **(%pred) mean** | **FEV_1_**  **(%pred) sd** | **FEV_1_**  **(L) mean** | **FEV_1_**  **(L) sd** |
| --- | --- | --- | --- | --- | --- | --- | --- | --- |
| Pavord ID, 2020 | 62 | AN ICS FABA | | D | . | . | 3.3 | 0.83 |
|  | 72 | AN-SABA | | A | . | . | 3.23 | 0.71 |
|  | 49 | R-ICS | | B | . | . | 3.29 | 0.89 |
| Hardy J, 2019 | 437 | R-ICS | | B | . | . | 3.03 | 0.9 |
|  | 448 | AN ICS FABA | | D | . | . | 3.03 | 0.88 |
| Beasley R, 2019 | 225 | R-ICS | | B | 91.2 | 13.8 | . |  |
|  | 220 | AN ICS FABA | | D | 91.4 | 14.1 | . |  |
|  | 223 | AN-SABA | | A | 89.1 | 13.9 | . |  |
| Bateman ED, 2018 | 2089 | R-ICS | | B | . | . | 2.75 | 0.76 |
|  | 2087 | AN ICS FABA | | D | . | . | 2.69 | 0.75 |
| O'Byrne PM, 2018 | 1277 | AN ICS FABA | | D | . | . | 2.63 | 0.74 |
|  | 1277 | AN-SABA | | A | . | . | 2.58 | 0.74 |
|  | 1282 | R-ICS | | B | . | . | 2.69 | 0.73 |
| Renzi PM, 2010 | 253 | R-ICS/LABA | | E | . | . | 3.17 | 0.04 |
|  | 263 | R-ICS | | B | . | . | 3.08 | 0.05 |
| Per-Olof Ehrs, 2010 | 36 | AN-SABA | | A | 91.2 | 11.89 | . |  |
|  | 34 | R-ICS | | B | 90.8 | 12.24 | . |  |
| Hoshino M, 2009 | 13 | R-ICS/LABA | | E | 94.8 | 8.9 | . |  |
|  | 14 | R-ICS | | B | 88.7 | 8.1 | . |  |
| Boulet LP, 2009 | 33 | R-ICS | | B | 104.8 | 7.25 | . |  |
|  | 33 | AN-SABA | | A | 100.1 | 7.25 | . |  |
| Tamaoki J, 2008 | 38 | R-ICS | | B | . | . | 3.69 | 0.21 |
|  | 36 | LTRA | | C | . | . | 3.64 | 0.23 |
| Reddel H, 2008 | 23 | R-ICS | | B | 96.43 | 4.37 |  |  |
|  | 21 | AN-SABA | | A | 87.35 | 4.37 |  |  |
| Chuchalin A, 2008 | 973 | AN-SABA | | A | . | . | . |  |
|  | 315 | R-ICS | | B | . | . | 3.24 | 0.84 |
|  | 970 | R-ICS/LABA | | E | . | . | 3.30 | 0.93 |
| Boonsawat W, 2008 | 154 | R-ICS | | B | . | . | 3.23 | 0.90 |
|  | 155 | AN-SABA | | A | . | . | 3.334 | 0.97 |
|  | 149 | R-ICS/LABA | | E | . | . | 3.37 | 0.95 |
| Stankovic I, 2007 | 40 | R-ICS | | B | . | . | 3.66 | 0.88 |
|  | 45 | AN-SABA | | A | . | . | 3.71 | 0.82 |
| Papi A, 2007 | 106 | R-ICS/SABA | | E | 89.49 | 1.21 | 2.93 | 0.08 |
|  | 109 | AN-SABA | | A | 88.58 | 1.34 | 2.97 | 0.08 |
|  | 118 | AN-ICS/SABA | | D | 92.23 | 1.05 | 3.11 | 0.08 |
|  | 122 | R-ICS | | B | 90.32 | 1.25 | 3.04 | 0.08 |
| Haahtela T, 2006 | 45 | AN ICS/FABA | | D | 104.2 | 5.34 | 3.59 | 0.18 |
|  | 47 | AN-SABA | | A | 99.2 | 5.34 | 3.25 | 0.18 |
| **First author** | **n** | **Treatment compared** | | **Code** | **FEV_1_**  **(%pred) mean** | **FEV_1_**  **(%pred) sd** | **FEV_1_**  **(L) mean** | **FEV_1_**  **(L) sd** |
| Zeiger RS, 2005 | 191 | R-ICS | | B | 96.9 | 11 | . |  |
|  | 191 | LTRA | | C | 92 | 9 | . |  |
| Bousquet J, 2005 | 320 | LTRA | | C | 90.17 | 12.2 | . |  |
|  | 325 | R-ICS | | B | 90.93 | 12.2 | . |  |
| Boushey HA, 2005 | 76 | LTRA | | C | 87.1 | 14.4 | . |  |
|  | 73 | AN-SABA | | A | 88.5 | 12.7 | . |  |
|  | 76 | R-ICS | | B | 94.5 | 12.6 | . |  |
| Strand AM, 2004 | 78 | R-ICS | | B | . | . | . |  |
|  | 72 | R-ICS/LABA | | E | . | . | . |  |
| Pauwels RA, 2003 | 3597 | R-ICS | | B | 89.79 | 17.59 | . |  |
|  | 3597 | AN-SABA | | A | 87.97 | 17.59 | . |  |
| O'Byrne PM, 2001 | 231 | AN-SABA | | A | 91.7 | 14.84 | . |  |
|  | 228 | R-ICS | | B | 94.14 | 14.19 | . |  |
|  | 239 | R-ICS/LABA | | E | 94.97 | 14.74 | . |  |
| Osterman K, 1997 | 37 | R-ICS | | B | 96.8 | 10.8 | 3.42 | 0.66 |
|  | 38 | AN-SABA | | A | 88.1 | 12.2 | 3.2 | 0.77 |

**Table 8** Data extracted for pooling AQLQ outcome in adolescents/adults

| **First author** | **n** | **Treatment compared** | **Code** | **AQLQ m** | **AQLQ sd** |
| --- | --- | --- | --- | --- | --- |
| Postma DS, 2011 | 220 | R-ICS/LABA | E | 6.06 | 0.84 |
|  | 210 | R-ICS | B | 6.2 | 0.75 |
|  | 222 | AN-SABA | A | 5.89 | 0.77 |
| Per-Olof Ehrs, 2010 | 34 | R-ICS | B | 6 | 0.96 |
|  | 36 | AN-SABA | A | 6 | 0.83 |
| Zeiger RS, 2005 | 191 | LTRA | C | 5.7 | 1 |
|  | 191 | R-ICS | B | 5.95 | 1 |
| Bousquet J, 2005 | 325 | LTRA | C | 5.45 | 1 |
|  | 325 | R-ICS | B | 5.67 | 0.95 |
| Boushey HA, 2005 | 73 | LTRA | C | 6.1 | 0.6 |
|  | 76 | R-ICS | B | 6.3 | 0.7 |
|  | 76 | AN-SABA | A | 6.2 | 0.6 |
