# Supplementary material for: Regular versus as-needed treatments for mild asthma in children, adolescents, and adults: a systematic review and network meta-analysis
Source: BMC Med. 2025 Jan 21;23:21. doi: 10.1186/s12916-025-03847-z (PMC11752773; doi:10.1186/s12916-025-03847-z)
Supplement: Supplementary file 3 — Additional file 3. PRISMA Checklist. [file 12916_2025_3847_MOESM3_ESM.docx]

| **Section and Topic** | **Item #** | **Checklist item** | **Location where item is reported** |
| --- | --- | --- | --- |
| **TITLE** | | |  |
| Title | 1 | Identify the report as a systematic review. | Title page  Page 6− Line 91 |
| **ABSTRACT** | | |  |
| Abstract | 2 | See the PRISMA 2020 for Abstracts checklist. | See abstract checklist |
| **INTRODUCTION** | | |  |
| Rationale | 3 | Describe the rationale for the review in the context of existing knowledge. | Pages 6−Lines 74-86 |
| Objectives | 4 | Provide an explicit statement of the objective(s) or question(s) the review addresses. | Page 6−Lines 86-88 |
| **METHODS** | | |  |
| Eligibility criteria | 5 | Specify the inclusion and exclusion criteria for the review and how studies were grouped for the syntheses. | Page 6-7−Lines 95-97 |
| Information sources | 6 | Specify all databases, registers, websites, organisations, reference lists and other sources searched or consulted to identify studies. Specify the date when each source was last searched or consulted. | Page 6−Lines 106-108 |
| Search strategy | 7 | Present the full search strategies for all databases, registers and websites, including any filters and limits used. | Additional file 1 Page 3-7 |
| Selection process | 8 | Specify the methods used to decide whether a study met the inclusion criteria of the review, including how many reviewers screened each record and each report retrieved, whether they worked independently, and if applicable, details of automation tools used in the process. | Page 6−Line 98, Figure 1 |
| Data collection process | 9 | Specify the methods used to collect data from reports, including how many reviewers collected data from each report, whether they worked independently, any processes for obtaining or confirming data from study investigators, and if applicable, details of automation tools used in the process. | Page 8−Lines 132- 133 |
| Data items | 10a | List and define all outcomes for which data were sought. Specify whether all results that were compatible with each outcome domain in each study were sought (e.g. for all measures, time points, analyses), and if not, the methods used to decide which results to collect. | Page 7-8-Lines 119-129 |
|  | 10b | List and define all other variables for which data were sought (e.g. participant and intervention characteristics, funding sources). Describe any assumptions made about any missing or unclear information. | Page 8−Lines 133-141  Additional file 1 Page 8-10 |
| Study risk of bias assessment | 11 | Specify the methods used to assess risk of bias in the included studies, including details of the tool(s) used, how many reviewers assessed each study and whether they worked independently, and if applicable, details of automation tools used in the process. | Pages 8-9−Lines 144-149 |
| Effect measures | 12 | Specify for each outcome the effect measure(s) (e.g. risk ratio, mean difference) used in the synthesis or presentation of results. | Page 9−Lines 151-156  Table 1, Table 2 |
| Synthesis methods | 13a | Describe the processes used to decide which studies were eligible for each synthesis (e.g. tabulating the study intervention characteristics and comparing against the planned groups for each synthesis (item #5)). | Additional file 1 Pages 14-17 |
|  | 13b | Describe any methods required to prepare the data for presentation or synthesis, such as handling of missing summary statistics, or data conversions. | Additional file 1 Page 13  Additional file 2 |
|  | 13c | Describe any methods used to tabulate or visually display results of individual studies and syntheses. | Page 9-Line 157-172 |
|  | 13d | Describe any methods used to synthesize results and provide a rationale for the choice(s). If meta-analysis was performed, describe the model(s), method(s) to identify the presence and extent of statistical heterogeneity, and software package(s) used. | Page 9−Lines 157-165 |
|  | 13e | Describe any methods used to explore possible causes of heterogeneity among study results (e.g. subgroup analysis, meta-regression). | Page 9−Lines 160-165 |
|  | 13f | Describe any sensitivity analyses conducted to assess robustness of the synthesized results. | Page 9−Line 165 |
| Reporting bias assessment | 14 | Describe any methods used to assess risk of bias due to missing results in a synthesis (arising from reporting biases). | Page 9−Lines 166, Page 10−Line 179 |
| Certainty assessment | 15 | Describe any methods used to assess certainty (or confidence) in the body of evidence for an outcome. | Page 10−Lines 183-184 |
| **RESULTS** | | |  |
| Study selection | 16a | Describe the results of the search and selection process, from the number of records identified in the search to the number of studies included in the review, ideally using a flow diagram. | Page 10−Lines 187-197  Figure 1 |
|  | 16b | Cite studies that might appear to meet the inclusion criteria, but which were excluded, and explain why they were excluded. | No such studies |
| Study characteristics | 17 | Cite each included study and present its characteristics. | Additional file 1 Table S5, Page 14-17 |
| Risk of bias in studies | 18 | Present assessments of risk of bias for each included study. | Page 11−Lines 199-202  Additional file 1 Table S3, Page 11-12 |
| Results of individual studies | 19 | For all outcomes, present, for each study: (a) summary statistics for each group (where appropriate) and (b) an effect estimates and its precision (e.g. confidence/credible interval), ideally using structured tables or plots. | Table 1, Table 2  Additional file 1 Page 18-30, 36-54 |
| Results of syntheses | 20a | For each synthesis, briefly summarise the characteristics and risk of bias among contributing studies. | Pages 11-18−Lines 204-373 |
|  | 20b | Present results of all statistical syntheses conducted. If meta-analysis was done, present for each the summary estimate and its precision (e.g. confidence/credible interval) and measures of statistical heterogeneity. If comparing groups, describe the direction of the effect. | Table 1, Table 2  Additional file 1 Page 37, 41-43, 48-50, 52-53 |
|  | 20c | Present results of all investigations of possible causes of heterogeneity among study results. | Page 10−Lines 234-239  Page 15−Lines 296-300  Page 15−Lines 316-319  Page 16−Lines 339-342  Additional file 1 Page 37, 41-43, 48-50, 52-53 |
|  | 20d | Present results of all sensitivity analyses conducted to assess the robustness of the synthesized results. | Page 12−Lines 239-240  Page 15−Lines 301-302  Additional file 1 Page 29 |
| Reporting biases | 21 | Present assessments of risk of bias due to missing results (arising from reporting biases) for each synthesis assessed. | Additional file 1 Pages 38, 44, 46, 51, 53 |
| Certainty of evidence | 22 | Present assessments of certainty (or confidence) in the body of evidence for each outcome assessed. | Table 1, Table 2  Additional file 1 Page 31-35 |
| **DISCUSSION** | | |  |
| Discussion | 23a | Provide a general interpretation of the results in the context of other evidence. | Pages 19-20−Lines 400-440 |
|  | 23b | Discuss any limitations of the evidence included in the review. | Page 21−Lines 466-470 |
|  | 23c | Discuss any limitations of the review processes used. | Page 21-22−Lines 461-480 |
|  | 23d | Discuss implications of the results for practice, policy, and future research. | Page 21−Line 457-460  Page 22−Lines 476-478 |
| **OTHER INFORMATION** | | |  |
| Registration and protocol | 24a | Provide registration information for the review, including register name and registration number, or state that the review was not registered. | Page 6−Lines 91-93 |
|  | 24b | Indicate where the review protocol can be accessed, or state that a protocol was not prepared. | Page 6−Lines 91-93 |
|  | 24c | Describe and explain any amendments to information provided at registration or in the protocol. | Page 6−Lines 91-93 |
| Support | 25 | Describe sources of financial or non-financial support for the review, and the role of the funders or sponsors in the review. | Page 27 |
| Competing interests | 26 | Declare any competing interests of review authors. | Page 27 |
| Availability of data, code and other materials | 27 | Report which of the following are publicly available and where they can be found: template data collection forms; data extracted from included studies; data used for all analyses; analytic code; any other materials used in the review. | Additional file 2 |

*From:*  Page MJ, McKenzie JE, Bossuyt PM, Boutron I, Hoffmann TC, Mulrow CD, et al. The PRISMA 2020 statement: an updated guideline for reporting systematic reviews. BMJ 2021;372:n71. doi: 10.1136/bmj.n71
